# Anatomical-connectivity-guided functional connectivity reveals task-relevant pathways during proactive task-switching via recurrent graph neural networks

**DOI:** 10.1186/s40708-026-00300-6

**Published:** 2026-04-26

**Authors:** Siyu Wang, Atsushi Miyata, Teruhisa Okuya, Hiroto Yanagawa, Ayaka Sakaki, Natsuhiro Ichinose, Takatsune Kumada

**Affiliations:** 1 Graduate School of Informatics, Kyoto University, 36-1 Yoshida-Honmachi, Sakyo-ku, Kyoto, 606-8501 Japan; 2 Panasonic Holdings Corporation, Osaka, Japan; 3 Kyoto Women’s University, Kyoto, Japan

**Keywords:** EEG, Recurrent graph neural network, Task-switching paradigm, Proactive control, Temporal expectation, Functional network, Anatomical network, Cognitive neuroscience

## Abstract

In an increasingly complex society, cognitive flexibility is essential for effective multitasking. We developed a novel approach—Anatomical-Connectivity-Guided Functional Connectivity (ACG-FC)—and applied it to the preparatory phase of a task-cueing paradigm to examine functional coordination mechanisms among brain regions critical for cognitive flexibility during proactive control and temporal expectation. This approach emphasizes the importance of inter-regional collaboration by integrating the cortical structural connectome, which reflects transmission pathways with electroencephalography (EEG) data within recurrent graph neural networks (RGNNs) to evaluate the functional relevance of brain regions and their associated neural connections. In generalizing signal pattern differences across proactive control processes, RGNNs outperformed other symbolic machine learning and deep neural network (DNN) models commonly applied to neurophysiological data, indicating that spatial information from the structural connectome enhances EEG pattern recognition during executive control. Meanwhile, single-population attribution analysis revealed that general proactive task preparation involves functional coordination within three synchronous ACG-FC subnetworks clustered around the frontoparietal "multiple demand" system. This supports multiple late-latency neural priming processes during execution preparation, associated with functionally distinct yet highly complementary subnetworks in the prefrontal, cingulate, and temporal cortices. Furthermore, dual-population attribution analysis indicated that scattered ACG-FC differences specifically underlie distinct modes of proactive control or target anticipation. ACG-FC extends conventional functional connectivity (FC) from signal-dependent pairwise analyses to signal-structure-dependent network analyses by using a group-level anatomical connectome as a structural prior to guide the extraction of functional interactions, offering a new perspective on task relevance between cognitive activities and their neural bases.

**Supplementary Information:**

The online version contains supplementary material available at 10.1186/s40708-026-00300-6.

## Introduction

Cognitive control refers to the capacity of individuals to voluntarily manage their mental processes for optimal allocation of attentional and perceptual resources to select behavioral strategies aligning with internal goals [30, 80]. Proactive control—a prospective mode of cognitive control—entails the preprocessing of relevant information to optimize goal-related performance before the occurrence of anticipated scenarios [13, 27]. Cognitive flexibility influences the extent to which proactive control preemptively updates cognitive states, and directly impacts individual performance in an ever-changing environment [30].

### Proactive control in the cue-to-target interval of task-switching paradigm

To effectively respond to continuously varying task demands, the brain adaptively updates both cognitive representations and neural activity levels [47]. A well-suited experimental paradigm for examining the proactive updating of cognitive states is the cued task-switching [65, 104, 129]; it leverages contextually relevant sensory stimuli (cue) to indicate which stimulus–response (S–R) mapping should be executed when behaviorally relevant task stimuli (target) are presented. To enhance perceptual categorization, the cognitive system should actively engage in proactive control during the cue-to-target interval (CTI) to adjust S–R mapping, preparing for the impending reactive execution [33]. The rule shifting during the CTI exemplifies goal-directed context updating facilitated by proactive control [32], involving multiple execution control processes such as interference suppression, attention allocation, working memory, and decision-making [89].

#### Cognitive demands related to proactive task-switching control

Compared to other goal-directed paradigms, such as the go/no-go or the oddball, the task-switching not only requires proactively inhibiting residual activation from past execution through sustained attention [77, 104], but also necessitates the proactive adjustment of internal representations by retrieving relevant perceptual categorization information from episodic memory with selective attention shortly before reactive execution [46, 65]. Specifically, the post-cue proactive control operates on two hierarchically distinct levels. The low-level sensorimotor control enables preparatory neural activities that potentially prime S–R pathways and motor-related regions according to follow-up task rules for faster automatic reactions and less decision-making [33]. Higher-order episodic control retrieves contextual memory and modulates working memory to align contextual comprehension with current task demands [33], essentially re-encoding instruction sets for low-level sensorimotor processes [65].

#### Neural substrates related to proactive task-switching control

The frontoparietal regions are an essential part of the "cognitive control network," responsible for a broad range of executive functions involved in goal-directed behavior [132]. Recent functional magnetic resonance imaging (fMRI) findings have suggested the existence of a large-scale brain system involved in cognitive control that extends beyond the frontal and parietal cortices. For instance, Cocchi et al. [24] defined two anatomically and functionally segregated brain systems potentially central to cognitive control: the frontoparietal and cingulo-opercular cortices. Owing to more complicated demands, the top-down process in the task-switching CTI involves more diverse and controversial neural structures and mechanisms [6, 60]; it not only requires the recruitment of higher-level cognitive control regions, such as the prefrontal and parietal cortices, for conflict detection [13], inhibitory control [142] and reconstruction of low-level S–R mappings [59], but also entails the interaction between posterior regions—namely, the temporal and frontoparietal cortices—to retrieve, integrate, and represent task-set information from contextual memory [6, 60, 134].

#### Electrophysiological signals related to proactive task-switching control

Using EEG and magnetoencephalography (MEG), several neurophysiological studies thus far have demonstrated that cognitive control is highly associated with low-frequency oscillatory activity, particularly within delta (2–4 Hz) and theta (4–8 Hz) bands [30, 83, 100]. Theta activation is considered to facilitate information integration and enable goal-directed control processes [114], while increased delta activity is linked to execution control in cognitively demanding situations [29, 49]. Compared to other goal-directed paradigms, due to the proactive modulation across a broader range of cognitive processes in task-switching CTIs, post-cue event-related potentials (ERPs) demonstrate greater diversity and higher intensity (as indicated by maximum amplitude or surface Laplacian values) in low-frequency oscillations [6, 39], including the domain-general P3 (350–400 ms post-cue), the switch-related late positive component (LPC, 500–850 ms post-cue), and the temporal expectation-related contingent negative variation (CNV, 900–1200 ms post-cue) [59, 61, 62, 113]. The various ERP components provide complementary cognitive regulatory information for the intricate neural mechanisms underlying proactive task-switching process. The domain-general P3 is a phase-locked neural activity elicited by external stimulus or event presentations, reflecting early perception and attention processes. The task-switching LPC is an endogenously induced, non-phase-locked neural activity whose duration and intensity fluctuate across individuals and trials; it synthesizes electrophysiological signals from multiple cognitive processes, representing long-range neural dynamics associated with proactive control [72]. LPC1 (550–600 ms post-cue) reflects context evaluation and conflict resolution during early CTI, while LPC2 (750–850 ms post-cue) exhibits explicit neural modulation in response to a ‘switch’ cue, which involves retrieving contextual memory and updating rule representation with high-order episodic control [68, 112]. The temporal expectation-related CNV component is a negative slow potential variation linked to anticipation and preparation regarding upcoming events, reflecting a gradual transition from high-level cognitive processing of cues to anticipating and preparing for forthcoming S–R mappings. During the proactive task-switching process, anticipatory preparation involves overcoming interference from irrelevant stimulus features and rule information in target trials. Therefore, the cognitive demands reflected by the CNV include not just target response but also potential inhibitory control.

### Neural dynamics correlates I: cue-locked ERPs during proactive task-switching

Since proactive control of updating perception–action rules is usually completed within 2000 ms, the temporal resolution of fMRI is not adequate for analyzing this instantaneous cognitive process [2]. Consequently, electrophysiological signals with superior temporal resolution are typically employed to capture rapid neural dynamics during CTI. The phase-locked P3 reflects rapid responses to and preprocessing of external task-switching cues and is primarily distributed in scalp topography with the frontal lobe as the center, indicating a neural basis for early S–R mapping updates [6]. Furthermore, the decomposition of cue-locked total EEG power demonstrates that non-phase-locked power effects in the late-latency period largely capture the signal differences between proactive rule-switching and rule-repeating [79]. This suggests that neural dynamics specific to proactive regulation and temporal expectation originate from late-latency endogenous, non-phase-locked EEG activity, as reflected by LPC and CNV [59]. Their variations are not significantly affected by stimulus perception but rather depend on the inter-regional coordination, information exchange, and resource reallocation elicited by the demand of updating S–R mappings [6, 26] LPC is considered a crucial neurophysiological correlate of proactive task-switching, reflecting the blended neural dynamics of multiple higher-order cognitive operations, such as memory retrieval and information integration [30, 86, 115]. However, cognitive control during proactive preparation is manifested more strongly in the temporal coordination of cross-regional neural oscillatory activity [30, 31], and the neural activation patterns reflected by ERP components are insufficient to comprehensively characterize the inter-regional functional coordination mechanisms underlying endogenous modulation.

### Neural dynamics correlates II: low-frequency FCs during proactive task-switching

Low-frequency FC is a key neural dynamics correlate that reflects neural interaction processes during executive control and is well suited to analyzing non-phase-locked power modulation [22, 79]. It is inferred and quantified based on dependencies between neural activity signals across different brain regions, using phase-based metrics such as the phase locking value (PLV) [70], phase lag index (PLI) [120], and pairwise phase consistency (PPC) [133], along with coherence-based metrics including coherence (Coh) [117] and imaginary coherence (ImCoh) [94]. Studies on proactive task-switching have primarily revealed that the switch-related LPC may be modulated in delta and theta bands by the context-updating mechanism, supported by a large-scale neural system composed of functionally distinct frontoparietal and cingulo-opercular networks [7, 40]. Phase-synchronized FC in the theta and delta bands can serve as neural correlates of proactive control during rule switching and repeating [30, 118, 132]. Due to increased cognitive demands, the FC of the frontoparietal and frontotemporal networks in the low-frequency band is anticipated to increase in response to the ‘switch’ cue [79, 139]. Behaviorally, the FC strength between regions such as the left inferior frontal junction (IFJ), bilateral superior posterior parietal regions, left precuneus, bilateral inferior parietal regions, and right middle frontal gyrus is negatively correlated with switch cost, thereby facilitating task-switching [79, 86, 140].

However, FC is based on second-order statistics, such as phase synchronization and coherence, and typically tests differences via linear, condition-wise comparisons. This methodological framework limits its capacity to characterize multi-regional joint participation and higher-order, nonlinear functional interactions. In addition, the unsupervised feature engineering on which FC metrics rely may introduce representation biases that are misaligned with task demands while also compressing the original neural information. More importantly, FC primarily focuses on examining neural interactions among cortical regions during proactive task-switching, rather than identifying the underlying neural substrates [25]. Neuroanatomical studies have revealed that the brain is a complex organ characterized by a physiological network, whose internal connections are primarily established through myelinated axons—namely, white-matter fibers [50]. Neural signal transmission among regions within widely distributed anatomical circuits forms an interconnected network that mediates executive function [125, 139]. Cognitive control depends on these cross-cortical information transmission pathways [37, 78], particularly within the white-matter structures of the frontoparietal and cingulo-frontal networks that are closely associated with voluntary coordination of cognitive resources [16]. Canonical FC methods assume that cortical regions form a discrete, unordered set, where each region is theoretically capable of functionally associating with any other region, resulting in $[n(n-1)/2]$ potential connections among $n$ regions of interest (ROIs) [56]. Across all topologically possible links, functional connections (FCs) constructed by selecting regional pairs that exhibit direct phase synchronization or time-domain correlations in task-state EEG signals do not explicitly reflect the conduction pathways underlying anatomical circuits that mediate neural interactions. Even when a structural connectivity template is applied as a post-hoc mask, the approach essentially preserves the synchronization strength of individual edges, making it difficult to characterize how multiple structural connections jointly contribute, at the network level, to task-related interaction patterns. Therefore, for cognitive processes such as proactive task-switching, which involve multiple concurrent neural activities distributed across widespread brain regions [64, 86, 111], relying solely on data-driven FC coordination patterns may overlook certain neural mechanisms associated with structure–function coupling [22, 126].

### Current research: neural substrates underlying neural dynamics correlates

While low-frequency ERPs and FC provide quantifiable correlates for characterizing the neural activations and cross-regional interactions underlying proactive task-switching control, they cannot explicitly specify the neural substrates on which these dynamics functionally depend. Accordingly, multimodal connectome research has attempted to explicitly incorporate anatomical information to obtain physiologically interpretable characterizations of functional interactions, including using diffusion-weighted structural connectivity as a mask or penalty when estimating FC [54, 98], and leveraging structural templates to improve the stability of directed FC estimation under low signal-to-noise conditions [96]. These approaches typically treat structural information as an estimation-level constraint to infer more reliable and sparser FC patterns.

In contrast, we view group-level structural connectivity as a prior template for organizing cortical source signals during DNN learning, and we use task-condition supervision to quantify the marginal contribution of each connectivity prior to discriminative neural dynamic interactions. This formulation turns the pathway-level support for task-relevant functional interactions into a comparable quantitative index. Following this rationale, the present study proposes a novel connectivity pattern, termed Anatomical-Connectivity-Guided Functional Connectivity (ACG-FC), to investigate the structure–function coupling mechanisms underlying proactive task-switching processes. FC captures functional coordination supported by both direct and indirect anatomical pathways and reveals region pairs showing significant differences in synchronization strength between conditions. In contrast, ACG-FC, guided by a macroscale anatomical prior with relatively reliable connection strengths and using task discrimination as supervised learning, learns interactive representations that can distinguish different proactive control modes and identifies the connective features that contribute most significantly to this discrimination. On this basis, ACG-FC provides structurally guided complementary interpretations for functional interactions or coordination. To this end, we tentatively examined two hypotheses during the signal epochs of proactive task preparation.

First, we posit that a group-level structural connectome can serve as a spatial-organization prior for cortical source-reconstructed EEG, thereby improving DNNs’ ability to identify task-relevant functional patterns. When EEG signals are mapped onto the cortical surface, they are often represented as a “tensor without topological constraints,” which causes inter-regional signals to lose permutation invariance due to the absence of explicit spatial-structural information. Consequently, DNNs may be limited in learning reproducible functional patterns across trials and participants [131, 141]. To address this issue, we introduce a group-level deterministic structural connectivity template to provide a principled way to spatially organize EEG source signals, and we validate—within task-switching CTIs—whether this structural prior enables DNNs to more effectively model EEG signal dynamics. From a modeling perspective, this structural template is equivalent to a form of regularization during feature learning, thereby reducing non-informative aggregation and enhancing the stability of representations across trials and subjects.

Conversely, task-optimized DNNs operating on graph-structured EEG sequences can quantify the informational value of structural-connectivity priors for functional pattern recognition. To this end, we employ model attribution methods—which probe how input features contribute to model performance—to develop attribution processes that evaluate the task-related functional relevance of connective features. We further examine whether these relevance measures can serve as ACG-FC, validating their effectiveness in providing a structural-level complement and extension to ERP and (posteriorly structurally masked) FC analyses.

## Methods

### Overall analytical framework

This study aims to investigate group-level deterministic structural connections that underlie proactive S–R mapping transitions, using task-switching EEG epochs in which distinct patterns of proactive control are reflected in non-phase-locked power modulations. To ensure that subsequent modeling and attribution analyses focus on signal segments containing the most task-relevant information, we first implemented a task-switching paradigm to collect cue-locked EEG data during CTIs. After preprocessing, global field power (GFP) analysis was applied across all electrodes to identify time windows in which neural dynamics elicited by different proactive control modes differed significantly, thereby enabling more precise localization of EEG epochs directly relevant to the research question. Subsequently, using the recognition performance of proactive control modes as an evaluation criterion, we examined the extent to which RGNNs, as models capable of handling graph-structured temporal sequences, could capture and exploit EEG signal dynamics within the topological space defined by the structural connectome, thus laying the foundation for extracting functionally relevant connective features from task-optimized models. To embed EEG dynamics into anatomical topology and achieve spatial alignment across brain regions, EEG signals were reconstructed in the cortical source space using weighted minimum norm estimation (wMNE) [91] combined with dynamic statistical parametric mapping (dSPM) [34], reorganized accordingly, and then embedded into the structural connectome.

Given that brain regions associated with cognitive control are primarily located in the cortex [14], this study adopted the group-level deterministic anatomical connectome proposed by Gong et al. [45], which was inferred from diffusion tensor imaging (DTI) [1] using the Desikan-Killiany atlas (DK atlas) [38], as the graph topological structure to guide the propagation of functional dynamics along interpretable macroscopic anatomical pathways. The performance of RGNNs incorporating this structural prior was compared on cortical EEG signals with that of other models that process time series without a structural prior. To further reverse-infer the neural bases underlying different modes of proactive preparation from task-optimized RGNNs, we developed Attribution Processes I and II (AP I and II) to distribute attribution scores throughout the neural circuit and to identify connectivity features with significant contributions as anatomical connectivity-guided functional connections (ACG-FCs), which characterize functional relevance guided by structural connectivity.

**Fig. 1:**
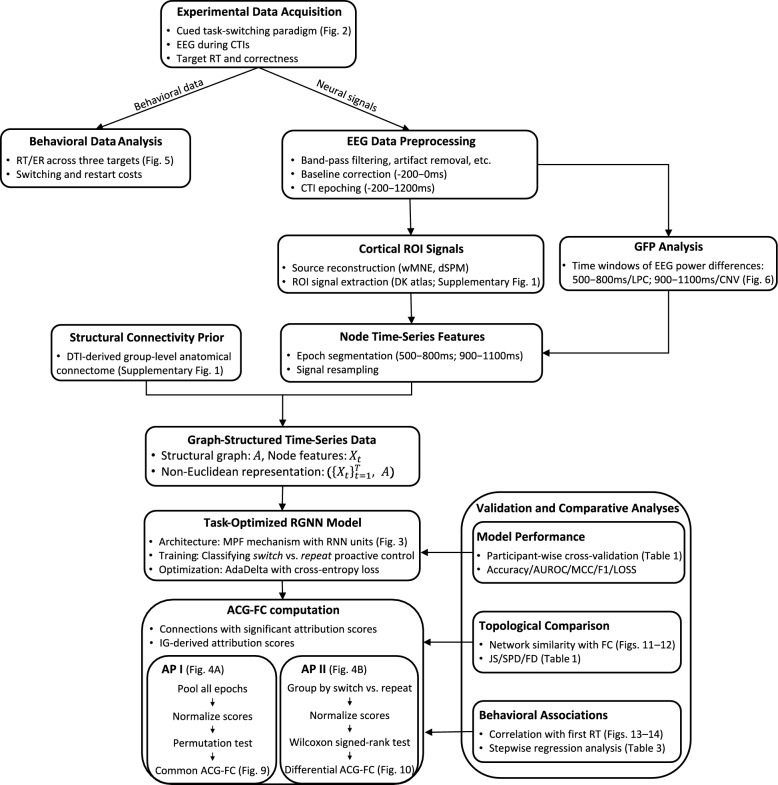
Overview of the ACG-FC Methodological Framework. *Note.* The figure organizes all experiments and analytical procedures employed in this study into a coherent workflow, highlighting their logical relationships

Finally, using reaction times (RTs) from the first target trial as an index of behavioral cost, we evaluated the effectiveness of the ACG-FC approach for studying proactive task-switching mechanisms by examining statistical correlations between significant ACG-FCs during target trials and RTs. We also introduced Anatomical-Connectivity-Masked Functional Connectivity (ACM-FC) as a post-hoc masked baseline and, together with network-topology comparisons, validated the complementary and incremental value of ACG-FCs relative to other FC networks and explored the potential executive functions associated with the identified ACG-FCs. Figure 1 illustrates the overall workflow encompassing all analytical components of the proposed methodological framework. Unless otherwise noted, all *t*-tests reported in this study were paired-sample *t*-tests comparing the same data across the two objects being evaluated.

### Dataset description

#### Participants

Twenty healthy, right-handed, native Japanese-speaking students from Kyoto University, aged 20–25 years (10 females, *M* = 21.45, *SD* = 1.20) participated in this study. They reported no history of neurological or psychiatric disorders and had no visual impairments such as color blindness or dry eye syndrome. Their vision was normal or corrected-to-normal. Informed consent was obtained from all participants. Experimental procedures and behavioral testing were conducted in accordance with the Declaration of Helsinki, with approval from the ethics committee of Panasonic Holdings Corporation.

#### Stimuli and procedure

Participants were seated in an electrically shielded and sound-attenuated room. They placed their hands on a response pad on the front table and directed their gaze toward a 19-inch WLED monitor (1280 × 1024 at 60 Hz) positioned approximately 58 cm away.

Presentation® software (Neurobehavioral Systems Inc., Albany, CA, USA) was used to display stimuli and collect behavioral responses. Both target and cue stimuli were centrally presented against a gray background and horizontally aligned with the line of sight. The target stimuli consisted of four colored shapes with identical areas (blue square, blue circle, red square, and red circle; 1° visual angle), presented with an equal probability in one block. The cue stimuli were two types of gray Gabor patches with either vertical or horizontal gratings (4 cpd frequency, 3.5 cd/m^2^ luminance, 1° visual angle), indicating whether participants should repeat or switch the previous rule. The mapping between grating orientations and instructions was counterbalanced among participants.

**Fig. 2:**
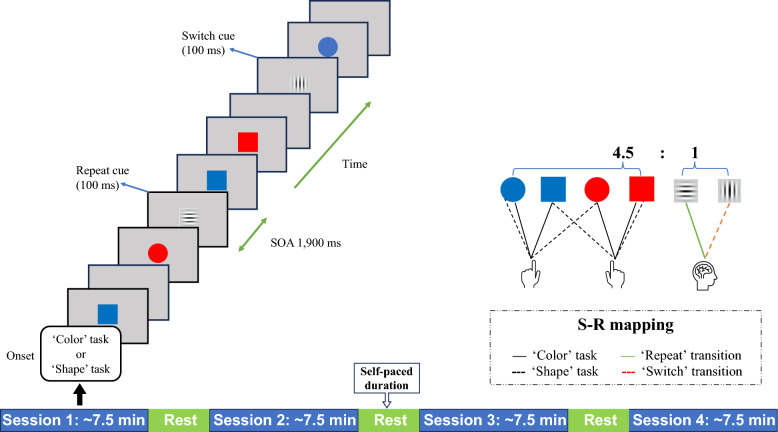
Experimental procedure and S–R mappings. *Note.* The experiment consisted of four sessions, each lasting approximately 7.5 min, with three self-paced rest intervals interspersed throughout. Each session commenced with a prompt specifying whether the initial task pertained to color or shape

The experiment was a variant of the intermittent-instruction paradigm [90, 113]. An example of the task sequence and the correctly active S–R mappings are provided in Fig. 2. The trial sequence was semi-randomly generated, consisting of 20 ‘repeat’ and 20 ‘switch’ cues, along with approximately 184 targets—the expected number under error-free conditions. A varying number of 3–6 target tasks were initially planned and placed between consecutive cues. Each cue was displayed at the center of the screen for 100 ms. After a 1,900 ms stimulus onset asynchrony (SOA) (cf. [41]), a target appeared at the exact center of the screen for 100 ms. Participants were instructed to fixate on the center of the monitor and respond to the target according to a color or shape categorization rule prompted via cues by pressing a key with either their left or right index finger. The instructions provided before each session emphasized the importance of response speed and accuracy in the target tasks.

The next display was presented either when participants responded to the targets or after 1,900 ms had elapsed. When an incorrect response was made or there was no response, the feedback display was presented for 2,000 ms. An additional target trial was then added to help participants adhere to the current S–R mapping rule and mitigate post-error propagation and slowing effects. Each session lasted approximately 35–40 min. Before the experiment, participants completed two practice blocks, each consisting of 48 targets and 10 cues, with a 2,000-ms feedback display following any error or delay.

#### Apparatus and EEG data acquisition

Although a standard 32‑channel EEG system offers moderate spatial resolution relative to high‑density montages, it can achieve source‑localization performance comparable to dense arrays for superficial cortical activity in the low‑frequency range (2–13 Hz) associated with cognitive control processes—such as the parietal delta rhythm during oddball tasks, the frontal alpha rhythm during attention tasks, and the frontal‑midline theta rhythm during executive tasks [35, 58]. The extra electrodes refine localization by only a few millimeters and improve the signal‑to‑noise ratio [58, 144]. Since proactive task-switching primarily involves cortical oscillations in the low‑frequency delta and theta ranges, continuous EEG data were recorded using the BioSemi ActiveTwo system (Amsterdam, NL) from 32 scalp electrodes (International 10–20 System), along with four additional electrodes for cardiac and earlobe monitoring. Data were recorded at a sampling rate of 1,024 Hz, and sensor offsets were maintained below 50 μV.

### Behavioral analysis and data preparation

#### Behavioral analysis

Behavioral analysis focused on RTs and ERs across target trials. The analysis was limited to the first three target trials, as behavioral costs typically reached an asymptote in later trials [90]. RTs were reported only for trials with correct responses, with excessively fast reactions (< 200 ms) being regarded as errors. For EEG analysis, we examined signal epochs from cues followed by three consecutive correct target trials (cf. [29]). Mean RTs and ERs were subjected to a 2 (cue: repeat, switch) × 3 (position: target 1–3) repeated-measures analysis of variance (ANOVA). The Greenhouse–Geisser (GG) correction was applied when the sphericity assumption was violated [130], with non-corrected degrees of freedom reported for readability. The accuracy of distinct cues was compared using a *t*-test. In addition, the differences in mean RTs and ERs between the first and third targets were calculated as the restart cost, whereas the difference between the first targets following ‘switch’ versus ‘repeat’ cues was defined as the switch cost [6].

#### EEG data preprocessing

We employed the neuroelectromagnetic analysis toolkit MNE-Python (v1.3.1, 10.5281/zenodo.592483) for data preprocessing. Initially, the EEG data were subjected to a band-pass filter, restricting the frequency range to 1–50 Hz to eliminate low-frequency drifts and high-frequency noise [79]. To alleviate edge effects, bilateral signal extension with mirror padding was applied. EEG epochs with nonstereotyped artifacts (such as cable movement and swallowing) and residuals exceeding ± 100 μV were excluded manually through visual inspection, whereas epochs with stereotyped artifacts (such as eye blinks, muscle artifacts, and cardiac artifacts) were retained. Independent component analysis (ICA) [9] was applied to identify and separate out ICA components associated with eye blinks and movements, thus reconstructing EEG sequences devoid of eye-related artifacts. Additionally, cardiac-related signals were recorded using two ECG channels, and their spatial signal projectors (SSPs) [128] were computed for cardiac artifact removal. Finally, raw electrode data were referenced against bilateral earlobes and then re-referenced offline to the common average reference across the entire EEG array. The preprocessing code, and the resulting cue-epoch EEG data (in “.fif” format) used for analysis are publicly available at https://osf.io/p2vka/.

#### GFP analysis

EEG signals from correctly responded cue trials were analyzed. In alignment with EEG data processing in CTIs by López et al. [79], the bandpass-filtered signals were segmented into EEG epochs of 1,400 ms (-200–1200 ms relative to cue onset) to adequately encompass potentially endogenous ERP components associated with cognitive control. The signals in the pre-cue period (-200–0 ms) were recruited as a baseline to correct the respective EEG epochs. The decomposition of cue-locked total EEG power revealed that signal differences mainly reflect non-phase-locked power effects in the late-latency window [26]. To further examine time-domain differences in global EEG activity elicited by distinct proactive control modes during the late-latency period, we used GFP to quantify the overall field intensity of cue-locked EEG signals across all electrodes [92], and described group-level results using 95% confidence intervals (CIs) [108]. To identify significant power differences between ‘switch’ and ‘repeat’ proactive control, we conducted *t*-tests on low-frequency GFPs at each time point, applying a significance level of *p* < 0.050 with false discovery rate (FDR) correction [11]. Accordingly, we identified distinct power processes between ‘switch’ and ‘repeat’ cues and detected significant time clusters to serve as masks for extracting EEG signals.

#### Source reconstruction

Likewise, source reconstruction was performed using MNE-python. We selected the ‘ico-5’ level segmentation of the FreeSurfer average cortical surface (fsaverage), comprising 10,242 vertices [28]. An average head model, including the scalp, outer skull, and inner skull layers, was calculated using the built-in symmetric boundary element method (BEM) [42]. To estimate noise levels in the recordings, we extracted signals from the pre-cue baseline phase and calculated the noise covariance matrix using the Ledoit–Wolf shrinkage estimator [73]. To solve the inverse problem, sources were determined by wMNE [91]; then, dSPM normalized the wMNE spatial estimates with the noise covariance to further mitigate depth bias [34]. This wMNE–dSPM pipeline is widely suitable for estimating large-scale FC networks, as it reduces spurious signal correlations induced by volume conduction [52]. Cortical regions serve as executive centers for primary cognitive processes [39]. Therefore, we used the DK atlas to partition the cortex surface into 34 ROIs as listed in Supplementary Table 2, excluding the cerebellum, basal ganglia, and brainstem. For the comparability with other task-switching literature, these regions are additionally mapped onto the Anatomical Automatic Labeling (AAL) atlas [106], the Talairach template [71], and Brodmann areas [82]. The EEG signal series for each ROI were extracted by averaging the dipole signals within that region [136].

#### Anatomical connectome

Cross-regional neural fiber connections serve as pathways to facilitate the efficient transmission of neural signals, which is essential for regulating inter-regional interactions and collaborations for goal-directed behavior [24, 116]. For this study, we considered cortical regions as network nodes and DTI-inferred connections as edges. Since anatomical fibers are susceptible to both genetic and environmental factors, inter-individual variability exists in structural connectivity [21]. Rather than deriving subject-specific structural connections for each participant, we adopted the macroscale structural network constructed by Gong et al. [45], which summarizes a group-level cortical connectome with relatively reliable connectivity across 80 healthy adults, as an anatomical prior (Supplementary Fig. 1C). This structural graph was used to organize the EEG source signals and to support RGNN modeling. Accordingly, the resulting ACG-FC reflects task-related functional connectivity guided by a macroscale structural prior, rather than individual-level anatomical connectivity. As shown in the adjacency matrix in Supplementary Fig. 1B, the nodes in this connective model are linked by 248 undirected edges, each representing an anatomical connection between the volumetric centroids of two brain regions. The anatomical network has a density of approximately 0.11 and exhibits a small-world property, providing insight into how the brain is physiologically organized as an entity through complex structural connections.

The raw EEG data were back-projected into time courses through source reconstruction, preserving low-frequency delta and theta oscillations. To reduce temporal complexity, the EEG signals were resampled to 100 Hz following the Nyquist theorem (the digital sampling rate must be at least twice the highest analog frequency under analysis) [121]. Using a group-level DTI–inferred anatomical connectivity template, we organized ROI-level EEG time series into graph-structured sequences, as detailed in the “Anatomical-Connectivity-Guided Message Passing” subsection.

### ACG-FC

#### Operational definition of ACG-FC

The basic analytical framework of ACG-FC uses a group-level anatomical connectome to provide spatial organization for source-level EEG and employs a task-optimized recurrent graph neural network (RGNN) model to infer the marginal contribution of connective features to functionally relevant pattern recognition. Let the deterministic connectivity template be $G=(V,E)$, with adjacency matrix $A\in {\{0, 1\}}^{N\times N}$, where $E=\{(i,j)\mid {A}_{ij}=1\}$ specifies the set of information-interaction pathways. For each cue-locked epoch $e$, the ROI signals are represented as a node-feature time series:

$$ X^{\left( e \right)} = \left\{ {X_{t}^{\left( e \right)} } \right\}_{t = 1}^{T} ,X_{t}^{\left( e \right)} \in { \mathbb{R}}^{N \times F} . $$

Guided by the topological matrix $A$, an RGNN performs condition classification:

$${f}_{\theta }\left({X}^{(e)},A\right)\to {\widehat{y}}^{(e)},{y}^{(e)}\in \left\{\mathrm{0,1}\right\},$$

where spatial interactions are modeled via information aggregation and propagation over $E$ through MPFs, and temporal dependencies are modeled via recurrent mechanisms.

After optimizing the model, the IG algorithm is applied to compute the attribution score of each structural-prior feature $(i,j)\in E$ for task-related pattern recognition:

$${\alpha }_{ij}^{(e)}={\mathrm{IG}}_{ij}\left({f}_{\theta };{X}^{(e)},A\right).$$

Accordingly, the ACG-FC value is defined as the normalized attribution score:

1 $${v}_{ij}^{(e)}=\mathrm{Norm}\left({\alpha }_{ij}^{(e)}\right)\in [\mathrm{0,1}],(i,j)\in E$$

where $Norm(\cdot )$ denotes normalization of the attribution values across all connective features within each epoch to yield a comparable marginal-contribution scale. In essence, ${v}_{ij}^{(e)}$ characterizes the informational value of the neural dynamic interaction along edge $(i, j)$ for the classification decision.

ACG-FCs are further defined as structural edges whose ACG-FC values are significantly higher than a random baseline (or show significant differences between two conditions) in group-level analyses. Regardless of cue labels, we aggregate ${v}_{ij}^{(e)}$ across epochs and compute the cross-epoch mean for each edge, ${v}_{ij}^{(e)}$. Under non-parametric testing, connections with FDR-corrected *p* < 0.050 are defined as Common ACG-FCs. We split epochs by cue into switch and repeat groups and compute the mean edge values for each group, ${\overline{v} }_{ij}^{\mathrm{sw}}$​ and ${\overline{v} }_{ij}^{rep}$, respectively, and define:

$$\Delta {v}_{ij}={\overline{v} }_{ij}^{\mathrm{sw}}-{\overline{v} }_{ij}^{rep},$$

to indicate the direction of the difference. Under the Wilcoxon signed-rank test, structural edges with FDR-corrected *p* < 0.050 are defined as Differential ACG-FCs, where $\Delta {v}_{ij}>0$ indicates that the connection contributes more to ‘switch’ pattern recognition, and vice versa.

In summary, this study adopts the attribution strength of structural edges in a task-optimized RGNN as an interpretable quantitative index of functional interactions. ACG-FCs thus characterize structurally guided functional pathways that are significant at the group level, enabling the identification of macroscale anatomical-connectivity-guided functional coordination mechanisms during proactive task-switching.

#### RGNN

**Fig. 3:**
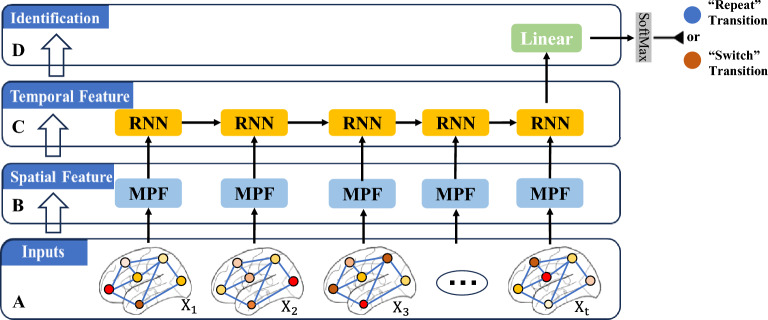
Illustration of modeling RGNN on unstructured EEG signal sequences. *Note.* **A** The schematic illustration of EEG activity variations at cortical nodes. EEG signals fluctuate over time at ROI nodes within a structural network. **B** MPF unit. Leveraging the topological structure provided by the anatomical connectome, the MPF unit applies graph convolutions (e.g., GCNs) via message passing and aggregation to capture spatial dependencies among nodes. **C** RNN unit. The node sequences embedded with spatial features are further processed by RNNs to attain temporal dependencies and refine representations through inter-unit information propagation. **D** Identification module. The probability distribution over categories is obtained by a fully connected layer with SoftMax

Graph neural networks (GNNs) learn node representations and capture relative positional information from graph-structured data by iteratively propagating information through message passing functions (MPFs) [44, 51]. By further integrating recurrent mechanisms (Fig. 3), such as long short-term memory (LSTM) [55] or gated recurrent unit (GRU) [23], RGNNs enable end-to-end representation learning for graph-structured time series. Unlike recurrent neural network (RNN) and convolutional neural network (CNN) frameworks, which convert EEG data into structured tensors [76, 146], RGNNs embed anatomical structural priors to transform EEG sequences into an unstructured graph-based data format. Through information propagation and updating along graph connections, RGNNs adequately capture time-varying inter-regional interactions in non-Euclidean space [137].

In this study, the RGNN uses group-level anatomical connectivity as a spatial organization prior, embedding cortical source EEG into the connectome topology. At each time point, the MPF, guided by structural pathways, performs neighborhood aggregation and applies nonlinear transformations to extract spatial features of multi-regional interactions. A recurrent unit then models the temporal evolution of network states, enabling the characterization of complex neural dynamics induced by proactive control at the level of higher-order, nonlinear spatiotemporal coupling. This modeling strategy is complementary to conventional FC: FC measures are built on phase synchronization or coherence and typically infer condition differences edge by edge via paired tests, making it difficult to express higher-order dependencies that emerge from multi-regional interactions unfolding over time. More importantly, within the RGNN framework, anatomical connectivity is not imposed as a post-hoc mask; rather, it serves as the input topological graph throughout the learning process. This allows the model to convert interaction information carried by anatomically feasible pathways within the cortical network into task-supervised functional relevance, going beyond the pairwise synchrony strength captured by FC, which may not be aligned with task performance.

In the empirical analysis, RGNN variants were employed to identify EEG signal patterns embedded in the structural connectome, including the temporal graph convolutional network (T-GCN) [145], which integrates a graph convolutional network (GCN) [67] with a GRU; EvolveGCN [95], which dynamically updates GCN parameters through RNNs to adaptively capture temporal changes in graph sequences; and DyGGNN [124], which handles dynamic graph-structured data. To examine the effect of group-level structural priors on pattern recognition, a first-order multivariate autoregressive model (MAR(1)) was additionally introduced as a baseline. Conventional machine learning (ML) algorithms, including LightGBM [63] and SVC [53], as well as DNN models commonly used to model multivariate time series—such as 1D-CNNs, RNNs, and TPA-LSTM [138]—were applied to the inversely traced signals without incorporating any structural priors for comparison.

##### Problem definition for cue-locked signal discrimination

To ensure the generalizability of our structure-dependent modeling, we formalized the signal pattern recognition problem during S–R mapping updates. The topological structure of the cortical network is represented as an unweighted graph$G=(V, E)$, where $V=\{{v}_{1},{v}_{2}, \cdots ,{v}_{N}\}$ denotes the node set (each ROI is treated as a node), $N$ is the total number of nodes, and $E$ denotes the edge set (each connection is treated as an edge). The adjacency matrix $A$ encodes all connections between ROIs, with$A\in {\{0, 1\}}^{N\times N}$: an element is marked as 0 to indicate the absence of a connection and 1 to signify its presence. The inversely reconstructed signals are regarded as attribute features of the corresponding ROIs and are denoted as${\mathbf{X}}_{\mathrm{T}}=\left({X}_{\mathrm{t}-\mathrm{p}+1}, \cdots \hspace{0.17em},{X}_{\mathrm{t}-1},{X}_{\mathrm{t}}\right)$, where $p$ represents the self-determined length of the historical time window, and ${X}_{\mathrm{t}}$ captures the regional signal feature across all ROIs at time$t$. The categorical variable is denoted as$Y\in \{\mathrm{0,1}\}$, where 0 and 1 represent repetition and switching of the preceding S–R mapping rule, respectively. The discriminator $H$ categorizes proactive control into either a ‘repeat’ or a ‘switch’ pattern based on the network topology $G$ and the feature matrix${\boldsymbol{X}}$, as formalized:

2 $$\begin{gathered} Y = H\left( {G;\user2{X}} \right) = H\left( {A;X_{{t - p + 1}} , \cdots ,X_{{t - 1}} ,X_{t} } \right) \hfill \\ = H\left( \begin{gathered} MPF\left( {A;X_{{t - p + 1}} } \right), \cdots \,, \hfill \\ MPF\left( {A;X_{{t - 1}} } \right),MPF\left(A;X_t \right) \hfill \\ \end{gathered} \right) \hfill \\ \end{gathered}$$

The schematic modeling process of implementing RGNNs to distinguish proactive perception–action rules underlying EEG signals is illustrated in Fig. 3. The cue-locked signal series was input into MPFs to extract spatial features based on the network’s topological structure and was processed by RNNs to capture dynamic features through information propagation across recurrent units. Ultimately, a fully connected layer with SoftMax activation yielded the probability distribution of the two categories. Therefore, ACG-FC is more directly aligned with a behavior-relevant functional criterion: the importance of a given structural edge is determined by its contribution to the model’s decision via cue-locked spatiotemporal interactions, rather than solely by statistically significant differences in synchronization strength. The Supplementary Materials elaborate on the modeling processes for various RGNN variants in identifying task-switching priming.

##### Anatomical-connectivity-guided message passing

After source reconstruction at the ROI level, EEG activity at each time point is organized as a node-attribute matrix ${X }_{\mathrm{t}}\in {\mathrm{R}}^{\mathrm{N}\times \mathrm{F}}$, representing $F$-dimensional EEG features (e.g., band amplitude or power) for $N$ ROIs at time $t$. Accordingly, a cue-locked epoch is represented as a graph-structured sequence ${\left\{({X}_{t}, A)\right\}}_{t=1}^{T}$. This representation provides the model with an explicit spatial organization prior, enabling temporal modeling under ordered pathway constraints and thereby avoiding the loss of permutation invariance that arises when cortical signals are fed into DNNs merely as topology-free tensors.

At each time step $t$, spatial dependencies among ROIs are modeled via an MPF implemented as graph convolution, which aggregates information from neighboring nodes guided by anatomical connectivity. The MPF implementations for each RGNN variant are described in the Supplementary Materials. We express graph convolution as a general Laplacian-based filtering operation, approximated by a $K$-order Chebyshev polynomial expansion [36]:

$${g}_{\theta }{*}_{G}{X}_{t}\approx \sum_{k=0}^{K} {\theta }_{k}{T}_{k}(\widetilde{L}){X}_{t},$$

where $\widetilde{L}$ denotes the scaled graph Laplacian derived from the adjacency matrix $A$, and ${T}_{k}(\cdot )$ denotes the $k$-th Chebyshev polynomial. This yields the spatially updated node representation:

$${H}_{t}=\sigma \left(\sum_{k=0}^{K} {T}_{k}(\widetilde{L}){X}_{t}{\Theta }_{k}\right),$$

where $\sigma (\cdot )$ is a nonlinear activation function and ${\Theta }_{k}$ are trainable parameters. Because $\widetilde{L}$ is inherited from a DTI-derived structural template, neighbor aggregation of node features is guided by deterministic structural pathways, thereby incorporating the anatomical connectome as a spatial prior into the model.

To capture the temporal evolution of anatomical-connectivity-guided spatial interactions within a signal epoch, we feed the spatially filtered features ${H}_{t}$ into a GRU to update the hidden state:

$${u}_{t}=\sigma \left({W}_{u}\left[{H}_{t},{h}_{t-1}\right]+{b}_{u}\right)$$

$${r}_{t}=\sigma \left({W}_{r}\left[{H}_{t},{h}_{t-1}\right]+{b}_{r}\right)$$

$${\widetilde{h}}_{t}=\mathrm{tanh}\left({W}_{h}\left[{H}_{t},\left({r}_{t}\circ {h}_{t-1}\right)\right]+{b}_{h}\right)$$

$${h}_{t}={u}_{t}\circ {h}_{t-1}+\left(1-{u}_{t}\right)\circ {\widetilde{h}}_{t}$$

where ${u}_{t}$ and ${r}_{t}$ are the update and reset gates, $\circ$ denotes the Hadamard product, and ${\{W}_{*},{b}_{*}\}$ are trainable parameters. The final hidden state ${h}_{T}$ serves as the cue-locked epoch representation learned under the spatial guidance of the structural template and is subsequently used by the output layer for condition discrimination.

##### Model implementation

The CNN- and RNN-based models were implemented in Pytorch (https://pytorch.org/), while RGNNs were deployed with Pytorch Geometric Temporal [110]. All models were optimized using the AdaDelta optimizer with cross-entropy loss [143]. The actual label and the identified probability of belonging to the positive class are herein denoted as ${y}_{i}$ and ${\widehat{p}}_{i}$, respectively, for the $i$ th sample. The loss function for all models consists of two terms, as shown in the following equation: the first term is the cross-entropy loss, which minimizes the discrepancy between actual and predicted labels. The second term is L2 weight decay regularization (λ = 0.0005), used to regulate model complexity. In this context, the training mini-batch size $(m$) is set to 32, *L* represents the number of network layers, and ${W}^{[l]}$ denotes the weight matrix of the $l$ th layer.

3 $$\begin{aligned} loss = & \frac{1}{m}\mathop \sum \limits_{{i = 1}}^{m} \left[ \begin{gathered} - y_{i} {\mathrm{log}}\left( {\hat{p}_{i} } \right) \hfill \\ - \left( {1 - y_{i} } \right){\mathrm{log}}\left( {1 - \hat{p}_{i} } \right) \hfill \\ \end{gathered} \right] \\ & + \frac{\lambda }{{2m}}\mathop \sum \limits_{{l = 1}}^{L} {\mid \mid }W^{{\left[ l \right]}} \left| {} \right|_{2}^{2} \\ \end{aligned} $$

A dropout layer with a rate of 0.2 was applied, and optimal hyperparameters were determined through cross-validation. Machine learning models were deployed using Scikit-Learn [97]. These models followed a two-stage pipeline: EEG-derived features were first extracted using three iterations of the spectrally weighted common spatial patterns (SpecCSP) algorithm, with the filter frequency range set to 2–13 Hz [102], and pattern recognition was then performed in the resulting feature space. The performance and stability of such two-stage approaches are influenced by the feature engineering. In this study, these methods were therefore treated as baselines and contrasted methodologically with end-to-end DNN-based learning. The classification outputs of the SVC and LightGBM models were converted into probabilities using Platt scaling before loss computation. The MAR(1) model is defined as ${X}_{t+1}=A{X}_{t}+{\varepsilon }_{t}$, where $A$ is constrained by the structural template such that only structurally connected ROI pairs participate in parameter learning. Separate MAR(1) models were fitted for each class using the training data, and the noise distribution was estimated from the regression residuals. For the test set, classification and loss estimation were performed by computing the log-likelihood of the signal time series under the MAR model corresponding to each class.

To compare recognition performance across models, we employed five metrics: accuracy, cross-entropy loss (LOSS), F1 score (F1), Matthews correlation coefficient (MCC), and the area under the ROC curve (AUROC). For robust evaluation, these metrics were computed as the mean and standard deviation (*SD*) across all validation sets using tenfold cross-validation. Each fold exclusively contained cue-locked EEG signal epochs from two unique participants, thereby satisfying the independence assumption of the validation sets.

#### Model attribution

RGNN models apply the group-level anatomical network to perform graph convolution, identifying proactive control modes. Next, the contribution of each connective feature to model decision-making was examined through attribution analysis on RGNNs, and the significant features were considered functionally relevant substrates for proactive task-switching.

The present study prioritized the Integrated Gradients (IG) algorithm as the attribution method for assigning attribution scores to connective features, thereby quantifying their functional relevance in task-specific pattern recognition and referring to those with significant attributions as ACG-FCs [93, 122]. The IG method combines the advantages of backpropagation- and perturbation-based approaches [12, 103], can be implemented solely through multiple applications of the standard gradient operator, and satisfies the axioms of sensitivity and implementation invariance [101]. Because RGNNs update node states via MPFs, significant attribution scores on ACG-FCs effectively indicate the contributions of signal interactions mediated by intrinsic connective features to cognitive pattern recognition.

The IG algorithm assigns importance scores—namely, attribution values representing the importance of contributions to model judgments—to individual features by approximating the gradient integrals of model outputs with respect to the inputs along a straight-line path from predefined zero-feature baselines to the actual inputs. For graph inputs, an adjacency matrix ${G}{\prime}$ that is identical to the original graph $G$ except that the connective features of interest are set to zero. In this study, the importance of connective features relative to the baseline is determined by the MPFs in conjunction with the signal input ${\boldsymbol{X}}$. Accordingly, the IG algorithm is applied directly to $MPF(G; {\boldsymbol{X}})$. The integrated gradient for the *i*-th connection of $G$ is defined by Eq. (3). Further details can be found in the Supplementary Materials.

4 $$\begin{gathered} {\mathrm{IntegratedGrads}}_{i} \left( G \right):: \hfill \\ = \Delta MPF_{i} \times \mathop \smallint \limits_{0}^{1} \frac{{\partial H\left( \begin{gathered} MPF\left( {G^{\prime};\user2{X}} \right) \hfill \\ + \alpha \times \Delta MPF \hfill \\ \end{gathered} \right)}}{{\partial MPF\left( {G_{i} ;\user2{X}} \right)}}d\alpha \hfill \\ \end{gathered} $$

where $$ \Delta MPF = MPF\left( {G;X} \right) $$ $$ - MPF\left( {G\prime ;X} \right),\Delta MPF_{i} = $$ $$ MPF\left( {G_{i} ;X} \right) - MPF\left( {G_{i}^{\prime } ;X} \right). $$

**Fig. 4:**
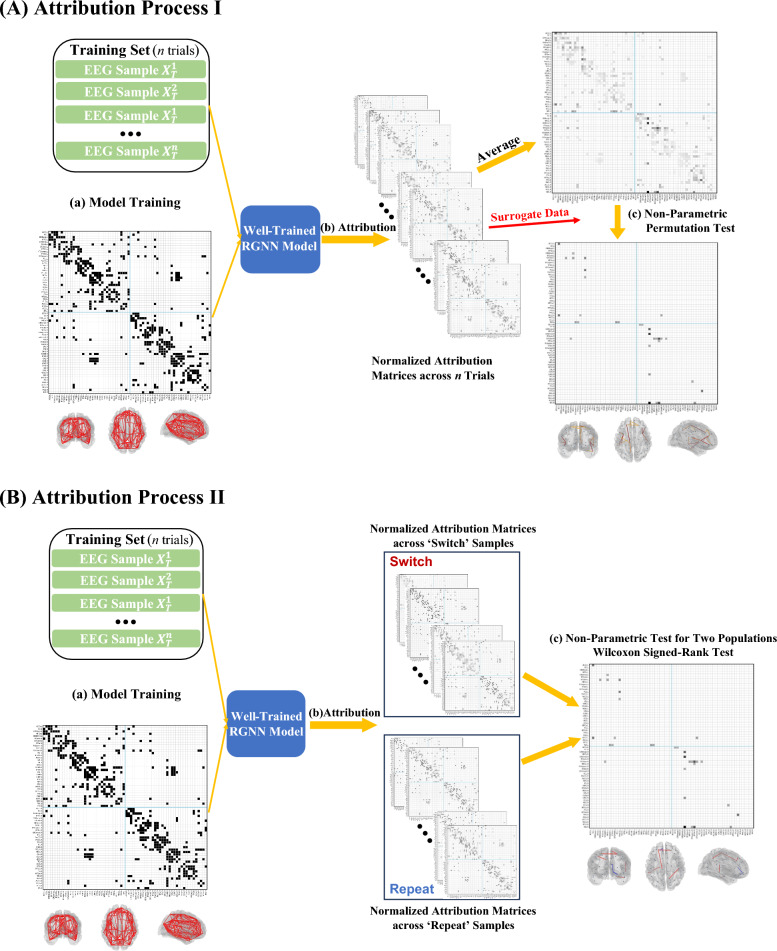
Overview of Attribution Processes for Two Types of ACG-FCs. *Note.* **A** AP I aims to detect Common ACG-FCs during proactive task-switching. Initially, EEG samples ${X}_{T}^{n}$ and a group-level cortical connectome serve as input features for supervised training (**a**). A series of normalized attribution matrices on the connectome matrix is derived from the task-optimized RGNNs for all EEG epochs (**b**). The average ACG-FC value for each connection is determined by the mean attribution scores across all epochs. A non-parametric permutation test is conducted on the surrogate dataset generated by shuffling attribution scores across edges in the anatomical template (**c**). Anatomical connections are considered ACG-FCs if the ACG-FC value attains a non-parametric, FDR-corrected *p* < 0.05. (**B**) AP II aims to identify Differential ACG-FCs between ‘switch’ and ‘repeat’ proactive control. Model training follows the same procedure as in AP I (**a**). EEG epochs are categorized into ‘switch’ and ‘repeat’ populations, from which two series of attribution score matrices are separately generated (**b**). The ACG-FC values for each connection are ascertained by averaging the attribution scores under the respective conditions. A Wilcoxon signed-rank test is employed to assess the differential significance of ACG-FC values between the two conditions, with FDR-corrected non-parametric *p* < 0.050 (**c**). In the illustrative brain representation, red ACG-FCs indicate significantly higher connectivity in the ‘switch’ condition than in the ‘repeat’ condition, whereas blue ACG-FCs indicate the opposite pattern

This study designed two distinct attribution approaches based on the IG algorithm: AP I, which identifies common neural substrates functionally associated with proactive adjustments to S–R mapping, and AP II, which determines differential neural substrates that exhibit significant differences in functional relevance between ‘switch’ and ‘repeat’ proactive preparation. For each cue-locked epoch, AP I applies min–max normalization to the attribution scores of all input edge features, yielding importance scores for proactive control mode recognition, which are referred to as ACG-FC values. Subsequently, all epoch-wise attribution results were aggregated into a single population, irrespective of the cue, to identify significantly relevant connections using a non-parametric permutation test [84], as illustrated in Fig. 4 (AP I). Specifically, the non-parametric permutation test first computed the mean ACG-FC value for each edge across epochs; subsequently, within each epoch, ACG-FC values were randomly reassigned across the set of structural edges defined by the anatomical template to generate surrogate samples. This surrogate data generation for all samples constitutes one permutation. In total, 10,000 permutations were performed to construct a null-hypothesis distribution in which edge contributions arose from random assignment under anatomical constraints. Functionally significant connections were then identified using a non-parametric threshold of p < 0.050 after FDR correction for multiple comparisons.

The other attribution analysis for examining the differential neural substrates between ‘switch’ and ‘repeat’ proactive preparation is illustrated in Fig. 4 (AP II). AP II segregated the total epoch set into ‘switch’ and ‘repeat’ subsets based on cues, then separately performed attribution and normalization on edge features to generate ACG-FC values for respective epoch populations. The Wilcoxon signed-rank test [107] assessed differences in ACG-FC values for each structural connection between ‘switch’ and ‘repeat’ conditions. Specifically, for each connection, ACG-FC values were first aggregated across all epochs under both cue conditions and then randomly shuffled into two surrogate populations of equal size to the original groups. For each structural connection, 10,000 permutations were performed to identify statistically significant differences (FDR-corrected non-parametric *p* < 0.050).

We employed Captum [69] to implement the IG algorithm for RGNNs. Within the macroscale anatomical prior, structural connections that significantly contribute to functional pattern recognition were defined as task-relevant ACG-FCs. By aggregating attribution results across all samples and conducting non-parametric permutation tests based on their average ACG-FC values, the Common ACG-FCs from AP I reflect the functional significance of the structural network in general proactive task preparation. Conversely, the Differential ACG-FCs from AP II highlight differences in functional relevance between ‘switch’ versus ‘repeat’ proactive control.

#### Topological comparison across (ACG-)FC networks

To clarify the complementarity of the proposed ACG-FC method to established FC analysis in terms of anatomical structure when exploring the neural mechanisms of proactive task-switching, we first conducted an in-depth comparison of their structural similarity across cue-locked epochs. These included phase-based connectivity measures (PLV, PPC) and coherence-based measures (Coh, ImCoh). First, these FC metrics were applied to the source-reconstructed signals during all cue-locked epochs, regardless of cue, to identify statistically significant common FCs (FDR-corrected *p* < 0.050). This is analogous to the objective of AP I—unveiling inter-regional functional interactions that generally occur during proactive task-switching (Common ACG-FCs). Subsequently, these metrics were separately applied to ‘repeat’ and ‘switch’ epochs to identify statistically significant differential FCs (FDR-corrected* p* < 0.050), which parallels the objective of AP II—elucidating variations in FC patterns under distinct proactive control modes (Differential ACG-FCs). In addition to visualizing connection patterns from sagittal, coronal, and axial views, we applied quantitative metrics, such as Jaccard similarity (JS), spectral distance (SPD), and Frobenius distance (FD), to assess the topological similarity between ACG-FC-derived networks and those derived from established FC metrics. Furthermore, for each window and edge attribute, we generated 5,000 random networks with the same number of edges as the corresponding ACG-FC network to form the null distribution and then applied non-parametric tests on the SPD metric—given its continuity, high variability, and broad value range—to examine the convergent validity of ACG-FCs with other FCs. We further applied an edge-wise consistency filter to the FC networks using a group-level anatomical connectivity template, retaining only FC edges supported by structural connections. This post-hoc structural-constraint procedure yields ACM-FC, which serves as a baseline for comparison with ACG-FC—where structural priors are injected during learning—within the same structural framework, but without any task-driven parameter learning.

#### Statistical correlation of (ACG-)FCs in relation to behavioral performance

We also sought to compare the statistical correlations of ACG-FCs and FCs with behavioral performance. For common (ACG-)FCs identified in the delta and theta bands during proactive switching control (500–800 ms) and target anticipation (900–1100 ms), we primarily examined both the absolute values of their correlations with the RT of the subsequent first target and the rank-order variation of these correlations. Compared with behavioral accuracy, the RT of the first target exhibits greater trial-level variability and directly reflects the effects of cue-related proactive control and temporal expectation, thereby serving as a behavioral measure to quantify the functional relevance of (ACG-)FCs.

For significant differential (ACG-)FCs within the two latency windows, we likewise focused on their correlations with the first-target RT, particularly the correlation differences between ‘switch’ and ‘repeat’ trials. In addition, differential (ACG-)FCs with higher connectivity in either ‘switch’ or ‘repeat’ trials were entered as variables in stepwise regression models of the first-target RT, in order to validate the overall functional relevance between (ACG-)FCs identified by different metrics and behavioral performance. Both the (ACG-)FC values and the first-target RTs were standardized into z-scores.

## Results

### Behavioral performance

Supplementary Table 1 reports the number of valid trials and ERs for each participant in the cue trials, as well as the RTs, ERs, and their respective differences across positions in the target trials. Despite individual differences, all participants performed efficiently, with 4.5% incorrect target trials (95% CI [3.8, 5.3]) on average.

**Fig. 5:**
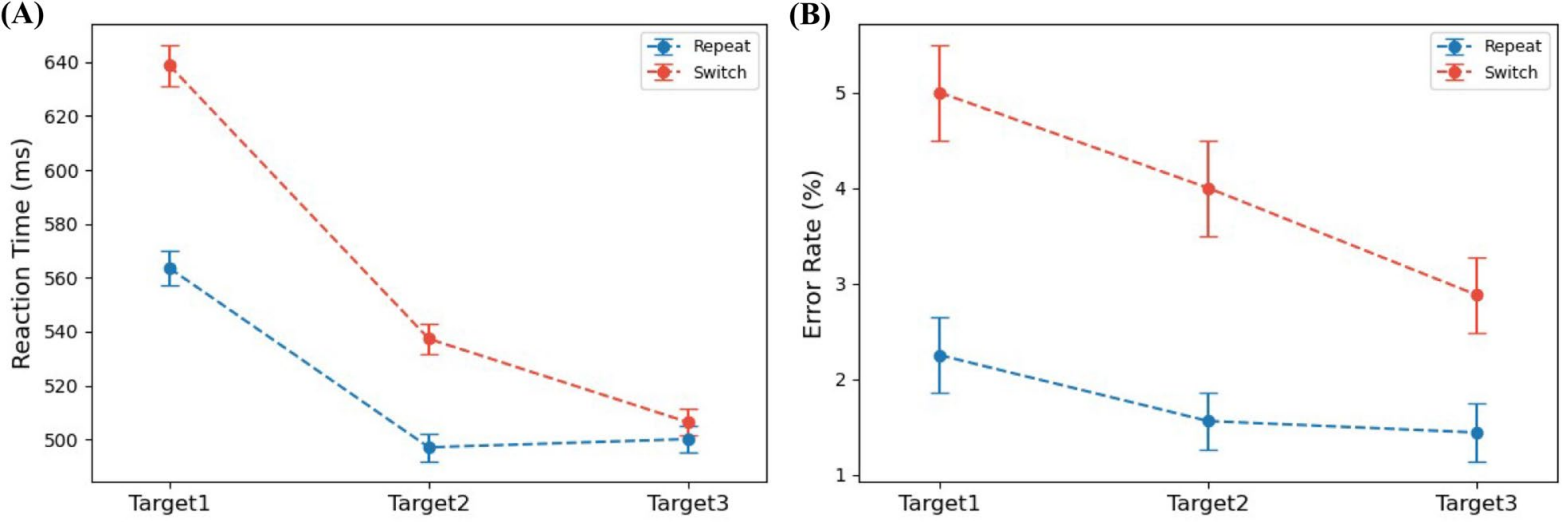
RTs and ERs for Initial Three Targets after Each Cue Type. *Note.* The mean RTs for correct targets (**A**) and the ERs for incorrect targets (**B**), together with their respective standard errors (SEs), are shown as functions of target position following either 'repeat' (blue line) or 'switch' (red line) cues

Figure 5A shows the group-level mean RTs for correct targets at the first three target positions following cues. The main effects of cue and target position were significant (*F*(1, 19) = 54.002, *p* < 0.001, η^2^ = 0.044; *F*(2, 38) = 30.586, GG-corrected *p* < 0.001, GG = 0.558, η^2^ = 0.175, respectively). Additionally, the cue × position interaction was significant (*F*(2, 38) = 13.859, GG-corrected *p* < 0.001, GG = 0.683, η^2^ = 0.023). Specifically, responses to the first post-cue target were markedly slower than those to the third target (repeat: 563.6 ms vs. 500.1 ms, *t*(19) = 4.538, *p* < 0.001; switch: 638.9 ms vs. 506.3 ms, *t*(19) = 5.702, *p* < 0.001), indicating significant restart costs regarding RT under both cue conditions. The restart cost was greater following the ‘switch’ than the ‘repeat’ cue (134.5 ms vs. 63.5 ms, *t*(19) = 4.195, *p* < 0.001). In addition, the RT to the first post-switch target was significantly slower than that to the first post-repeat target (638.9 ms vs. 563.6 ms, *t*(19) = 6.498, *p* < 0.001), revealing a pronounced switch cost in RT.

In terms of accuracy, the cue exhibited a significant main effect (*F*(1, 19) = 57.949, *p* < 0.001, η^2^ = 0.189), confirming ER differences between the two cues (Fig. 5B). The main effect of target position was only modestly significant (*F*(2, 38) = 4.039, GG-corrected *p* = 0.032, GG = 0.867, η^2^ = 0.065), indicating improved accuracy as the target progressed. The cue × position interaction was not significant (*F*(2, 38) = 1.092, GG-corrected *p* = 0.329, GG = 0.701, η^2^ = 0.015). A significant restart cost appeared under ‘switch’ cues, as the ER for the first target was notably higher than that for the third (5.00% vs. 2.88%, *t*(19) = 2.254, *p* = 0.018). In contrast, under ‘repeat’ cues, the restart cost was not significant (2.25% vs. 1.44%, *t*(19) = 1.303, *p* = 0.104), nor was the difference in restart cost between ‘switch’ and ‘repeat’ cues (2.12% vs. 0.81%, *t*(19) = 1.253, *p* = 0.113). Additionally, a significant difference in ER was observed between the first targets following 'switch' and 'repeat' cues (5.00% vs. 2.25%, *t*(19) = 3.297, *p* = 0.004), suggesting pronounced switch costs in ER.

Successful proactive control of S–R mapping is strictly defined as achieving the correct response to a cue only if three consecutive target trials following a preceding cue are answered correctly. This ensures that post-cue epochs used for EEG analysis originate from trials with correctly and proactively updated S–R mapping rules. Each participant maintained at least 80% accuracy in the first three target trials (Supplementary Table 1 for the number of successful cue trials per participant), providing 2,942 epochs (repeat: switch = 1519:1423) for subsequent modeling analysis. Each participant completed at least 64 effective cue trials per cue condition (Supplementary Fig. 3). The accuracy of cue transitions in the 'repeat' condition (*M* = 0.926, *SD* = 0.038) was significantly higher than that in the 'switch' condition (*M* = 0.869, *SD* = 0.084), with a *t*(19) = 4.796, *p* < 0.001.

### Cue-locked GFP difference

**Fig. 6:**
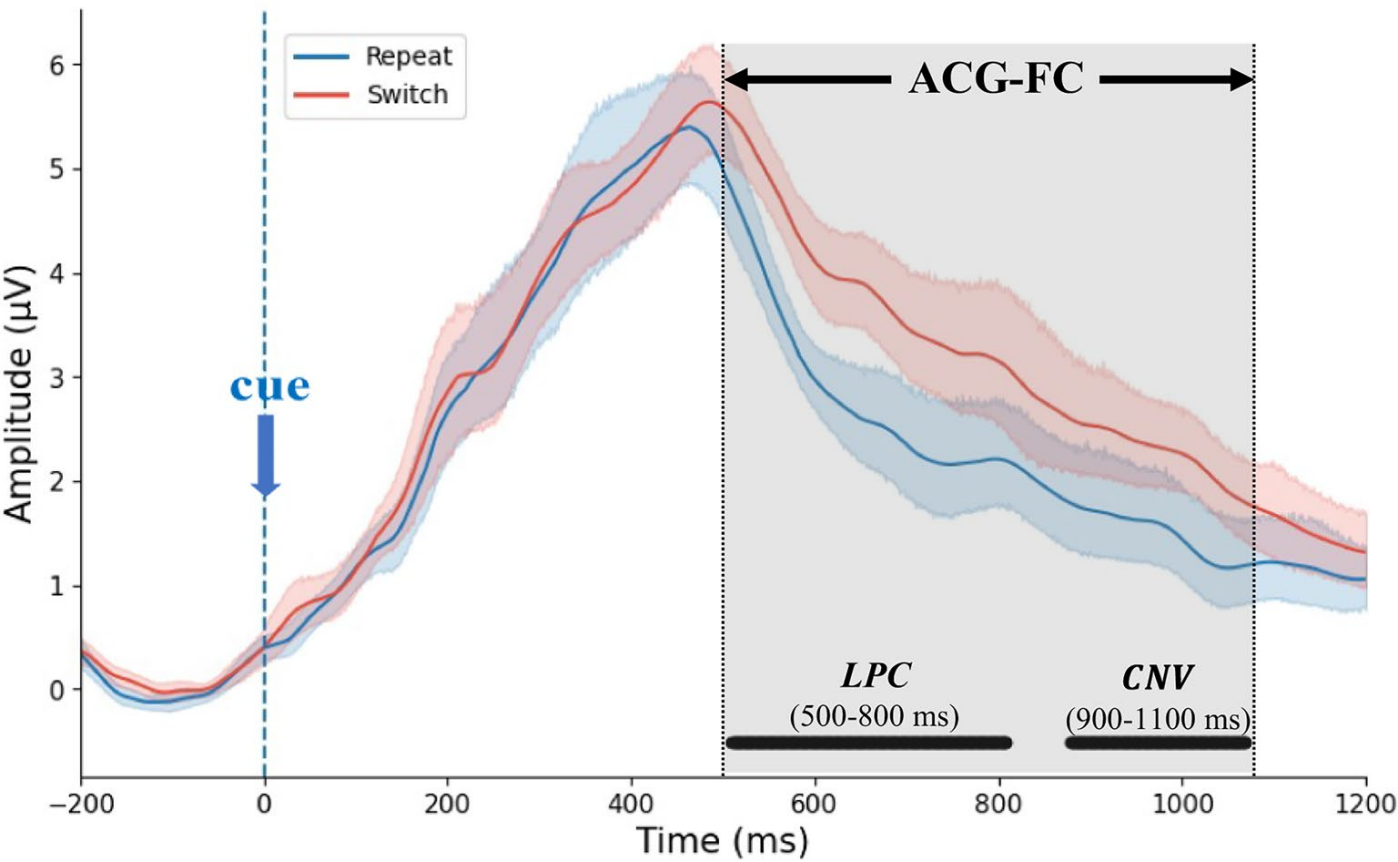
Global field power induced by two transition demands during cue-locked epochs. *Note.* The grand mean GFP curves elicited by the 'switch' (red line) or 'repeat' (blue line) cues are plotted together with 95% CIs. Black dots along the x-axis indicate time points where a statistically significant *t*-test (FDR-corrected *p* < 0.050) was observed, forming two continuous late-latency windows: “500–800 ms” and “900–1100 ms”. The blue vertical dashed line at 0 ms marks the cue onset

This study analyzed total power effects during cue-locked EEG epochs in two transition conditions using GFP analysis. A *t*-test with multiple comparison correction pinpointed two precise task-specific latency windows, 510.74–808.59 ms, and 878.90–1068.35 ms, at a significance level of 0.05. These two continuous late-latency windows corresponded to the switch-related LPC and CNV endogenous components, respectively, capturing global differences in non-phase-locked EEG power induced by distinct proactive task-switching demands. For readability in subsequent comparison and discussion, two windows are herein labeled as “500–800 ms” and “900–1100 ms”, respectively, as indicated in Fig. 6.

### Model performance

**Fig. 7:**
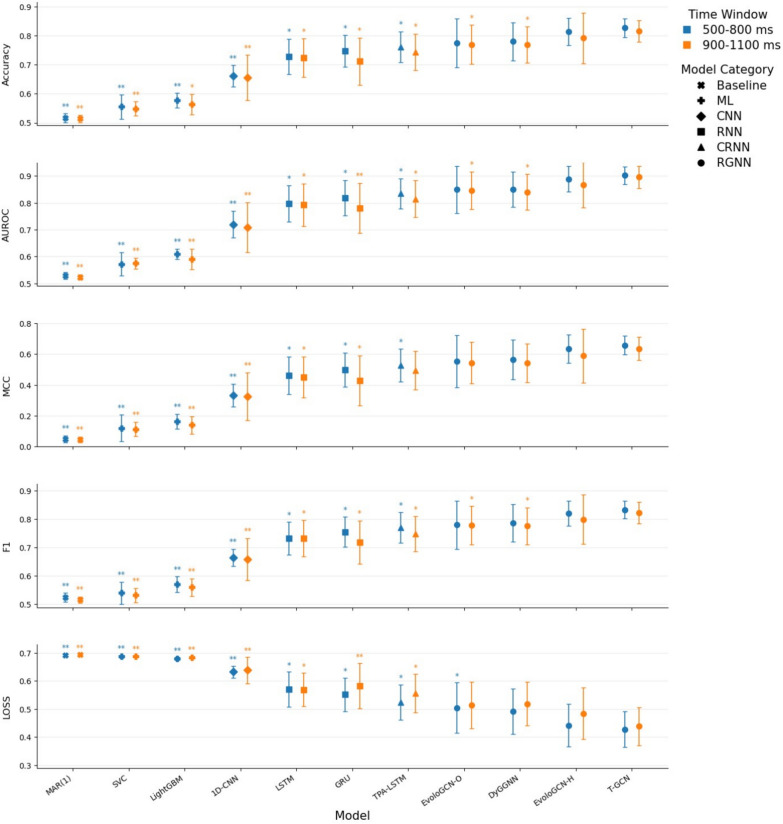
Model performance comparison on the pattern recognition of proactive task-switching*. Note.* Mean (± *SD*) across all validation sets in the cross-validation procedure is shown for each evaluation metric for all models in the 500–800 ms and 900–1100 ms time windows. Marker color indicates the time window, and marker shape indicates the model category. */** indicate that, based on one-sided *t*-tests across cross-validation folds, the performance of the corresponding model on a given evaluation metric was significantly lower than that of T-GCN, with significance levels of *p* < 0.010 and *p* < 0.001, respectively. Detailed numerical results are provided in Supplementary Table 3

Based on behavioral and time-locked EEG analyses, we used low-frequency EEG signals (2–13 Hz) from 2942 cue-locked epochs to train models for classifying proactive control modes within the late-latency windows of 500–800 ms and 900–1100 ms, with cues as ground truth supervision. The model comparison results are presented in Fig. 7.

RGNNs generally outperformed other models across all performance metrics. Within the two latency windows characterized by significant differences in EEG activity, even the lowest-performing RGNN variant, EvolveGCN-O, achieved higher accuracy than the CRNN-based TPA-LSTM by 0.013 and 0.026 on average, and yielded lower validation loss by 0.020 and 0.042, respectively. The T-GCN architecture exhibited the best mean performance across validation sets on all metrics and demonstrated statistically significant advantages, to varying degrees, in recognizing “repeat” and “switch” transition patterns compared with most other models. Within 500–800 ms post-cue, T-GCN achieved a recognition rate of 0.827 and an AUROC of 0.902. Relative to TPA-LSTM—which captures temporal features while extracting spatial features via structural convolution—T-GCN reduced validation loss by approximately 0.097 on average (*t*(9) =  − 4.335, *p* = 0.002) and improved accuracy and AUROC by about 0.065 (*t*(9) = 3.349, *p* = 0.006) and 0.067 (*t*(9) = 3.323, *p* = 0.006), respectively. Within 900–1100 ms, T-GCN reduced validation loss by 0.118 on average (*t*(9) =  − 4.690, *p* = 0.001) and improved accuracy and AUROC by approximately 0.072 (*t*(9) = 2.996, *p* = 0.010) and 0.081 (*t*(9) = 3.260, *p* = 0.007), respectively, relative to TPA-LSTM. Compared with EvolveGCN-O, which emphasizes structured feature evolution, T-GCN increased accuracy by about 0.052 (*t*(9) = 2.243, *p* = 0.032) and 0.046 (*t*(9) = 3.899, *p* = 0.003) across the two windows, respectively.

### Connective feature attribution

The task-optimized T-GCN excelled with superior pattern recognition capability, reliably capturing spatiotemporal dependencies in unstructured signal sequences. The essential connective features for control mode recognition were attributed to ACG-FCs crucial for signal feature interaction. Their contribution was quantified by ACG-FC values derived from the IG algorithm, reflecting their functional relevance to proactive task-switching.

#### ACG-FCs functionally related to proactive task-switching

**Fig. 8:**
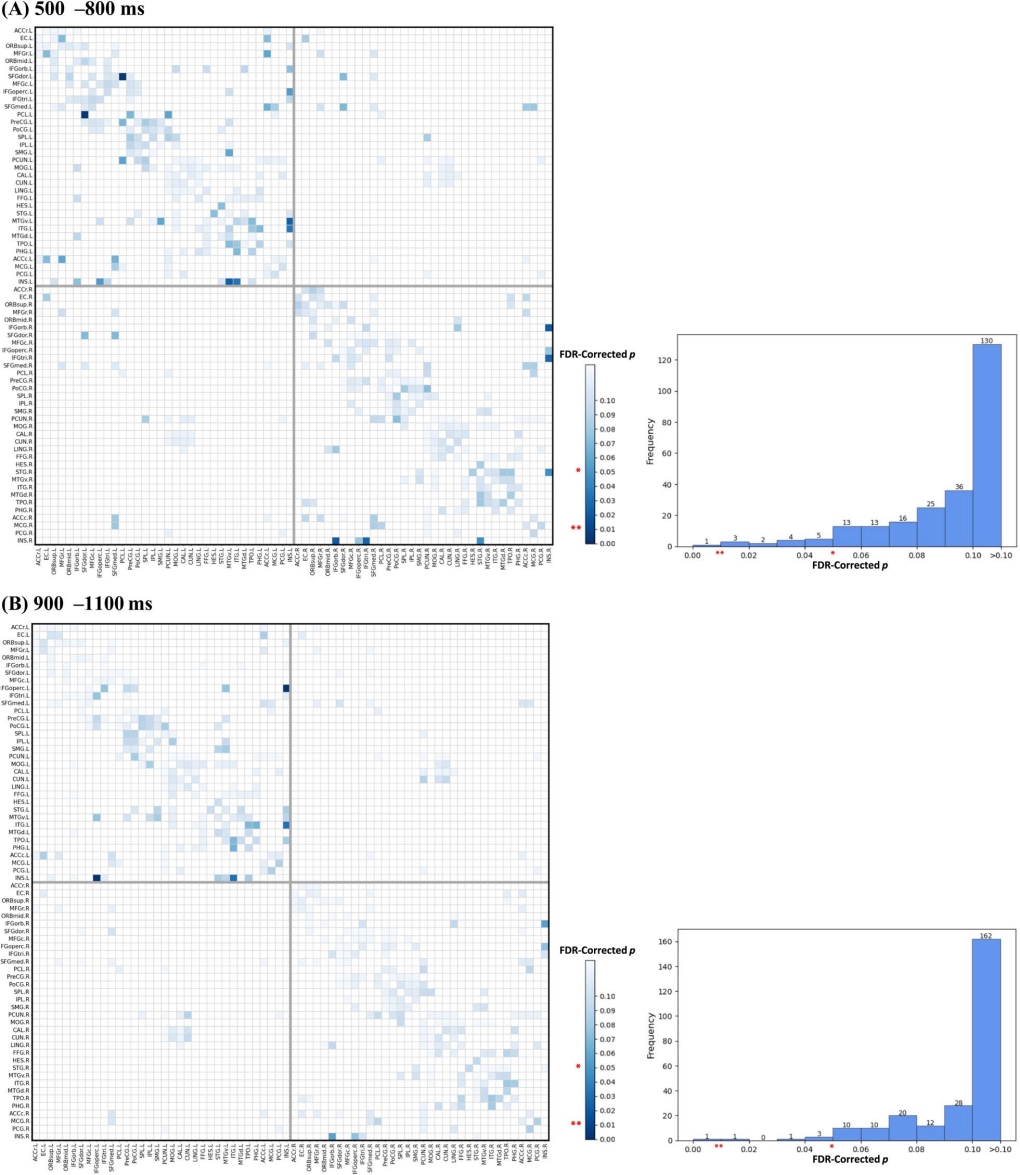
Non-parametric permutation tests for ACG-FC values on the anatomical connectome. *Note.* The two left plots in the panel display non-parametric test matrices for ACG-FC values within two windows: **A** 500–800 ms and **B** 900–1100 ms post-cue. Each matrix entry represents the significance of a given connection’s contribution to the T-GCN in identifying the control mode. Two intersecting gray lines at the center of the matrix delineate the left and right hemispheres, with the upper/left section indicating regions of the left hemisphere and vice versa. The suffix '.L' or '.R' following region names denotes the left and right hemispheres, respectively. The darker blue at the bottom of the color bar indicates higher levels of significance, with ‘*’ and ‘**’ denoting FDR-corrected *p*s < 0.05 and < 0.01, respectively. The two histograms in the right panel, corresponding to the non-parametric test matrices, display the frequency distribution of corrected *p*s across the ACG-FC values of all connections

Structural connections with statistically significant ACG-FC values in the non-parametric permutation test (FDR-corrected *p* < 0.050) were deemed essential in identifying proactive control modes and were termed Common ACG-FCs, as illustrated in the non-parametric test matrix in Fig. 8. The histogram in Fig. 8 depicts the distribution of FDR-corrected *p*s for all ACG-FC values. More Common ACG-FCs were observed in the 500–800 ms window (15) than in the 900–1100 ms window (6). Figure 10C specifically presents the values of these ACG-FCs, whereas Fig. 10A and B visualize their anatomical distribution in the cortex.

Within 500–800 ms post-cue, Common ACG-FCs critical for proactive pattern recognition involved regions and connections within the frontoparietal and temporoparietal lobes, forming three progressively sparser and independent subnetworks. Subnet 1 was mainly located in the frontal lobe and was comprised of eight ACG-FCs among seven regions, including the connections between the left and right dorsolateral superior frontal gyrus (SFGdor), the left medial superior frontal gyrus (SFGmed) and right SFGdor, the left caudal anterior cingulate cortex (ACCc) and left SFGmed, the left rostral middle frontal gyrus (MFGr) and left ACCc, the left paracentral lobule (PCL) and left precentral gyrus (PreCG), the left PCL and left SFGdor, as well as the left precuneus (PCUN) and left PCL. Despite its minimal ACG-FC value within Subnet 1, the ACG-FC between the medial and dorsolateral parts of the SFG served as a bridge between the left and right hemispheres. The ACG-FC that contributed the most to classification was located between the left MFGr and left ACCc in Subnet 1. Subnet 2, situated in the left hemisphere, linked the temporal and parietal lobes via the ACG-FC between the left supramarginal gyrus (SMG) and ventral middle temporal gyrus (MTGv). It consisted of five ACG-FCs connecting six regions, including the connections between the inferior temporal gyrus (ITG) and each of the parahippocampal gyrus (PHG), right opercular inferior frontal gyrus (IFGoperc), and insular cortex (INS), the SMG and MTGv, as well as the MTGv and INS. Among these, 'MTGv.L–INS.L' contributed the most in recognizing the proactive task-switching pattern. Subnet 3, entirely situated in the right frontotemporal lobe, was centered on the right INS, which facilitated connectivity with the right superior temporal gyrus (STG), triangular inferior frontal gyrus (IFGtri), and orbital inferior frontal gyrus (IFGorb) through ACG-FCs. Among these, 'IFGorb.R–INS.R' was the most influential connection for proactive task-switching.

Within 900–1100 ms post-cue, the number of significantly contributing ACG-FCs decreased compared to the preceding window, and were primarily distributed across the left temporoparietal and right frontal lobes; they were organized into two increasingly sparse subnetworks: Subnets 2 and 3, as illustrated in Fig. 10B. Subnet 2 was centered around the left ITG in the left temporoparietal area and consisted of four ACG-FCs across five regions, connecting the left ITG to the left temporal pole (TPO), PHG, and INS. The ACG-FC between the left IFGoperc and INS exhibited the largest ACG-FC value within this subnetwork. Subnet 3 was independently located at the junction of the right frontal and insular lobes, consisting of only two ACG-FCs between the right INS and the opercular and orbital parts of the right IFG. The connection to the orbital part had a higher ACG-FC value.

#### ACG-FCs functionally related to the variability of proactive task-switching

**Fig. 9:**
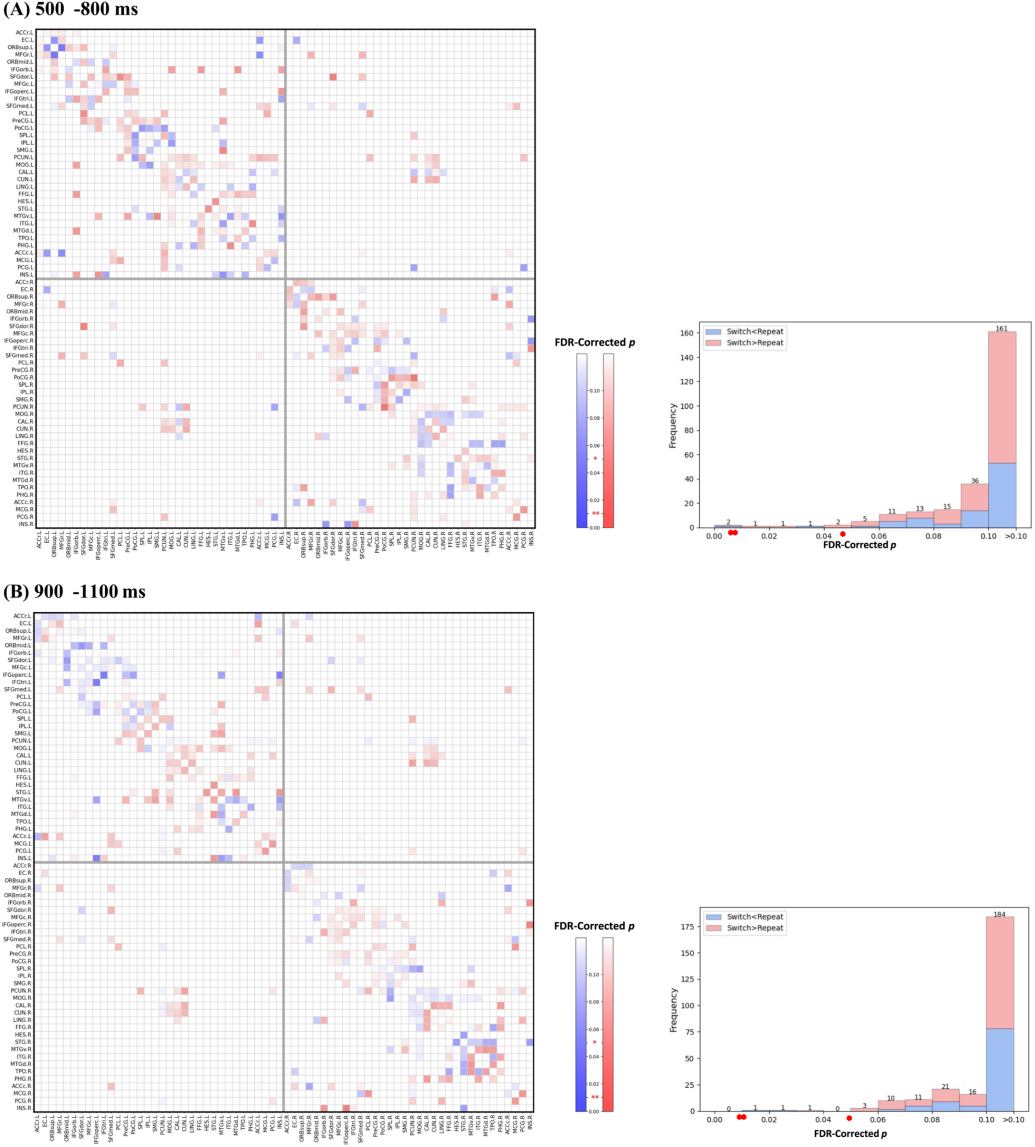
Wilcoxon signed-rank tests for ACG-FC-value differences between ‘switch’ and ‘repeat’ conditions. *Note.* The adjacency matrix displays FDR-corrected non-parametric *p*s for ACG-FC-value differences between two task-switching conditions in two windows: 500–800 ms (**A**) and 900–1100 ms (**B**). Each entry in the adjacency matrix represents the significance level of ACG-FC-value differences for T-GCN identifying 'switch' and 'repeat' signals. Higher ACG-FC values for 'switch' compared to 'repeat' cues are shown in red, while lower values in blue. In the accompanying color bar, darker colors indicate higher levels of statistical significance, with ‘*’ and ‘**’ denoting FDR-corrected *p*s < 0.05 and < 0.01, respectively. Two intersecting gray lines divide the non-parametric test matrix into cerebral hemispheres in the middle: the upper/left part corresponds to the left hemisphere regions, indicated by the suffix '.L,' while the lower/right part corresponds to the right hemisphere regions, indicated by the suffix '.R.' The histogram on the right panel, corresponding to the adjacency matrix, illustrates the distribution of corrected *p*s for ACG-FC-value differences across all connections. Red indicates connections where ACG-FC values are higher in the 'switch' than in the 'repeat' condition, whereas blue represents the opposite

**Fig. 10:**
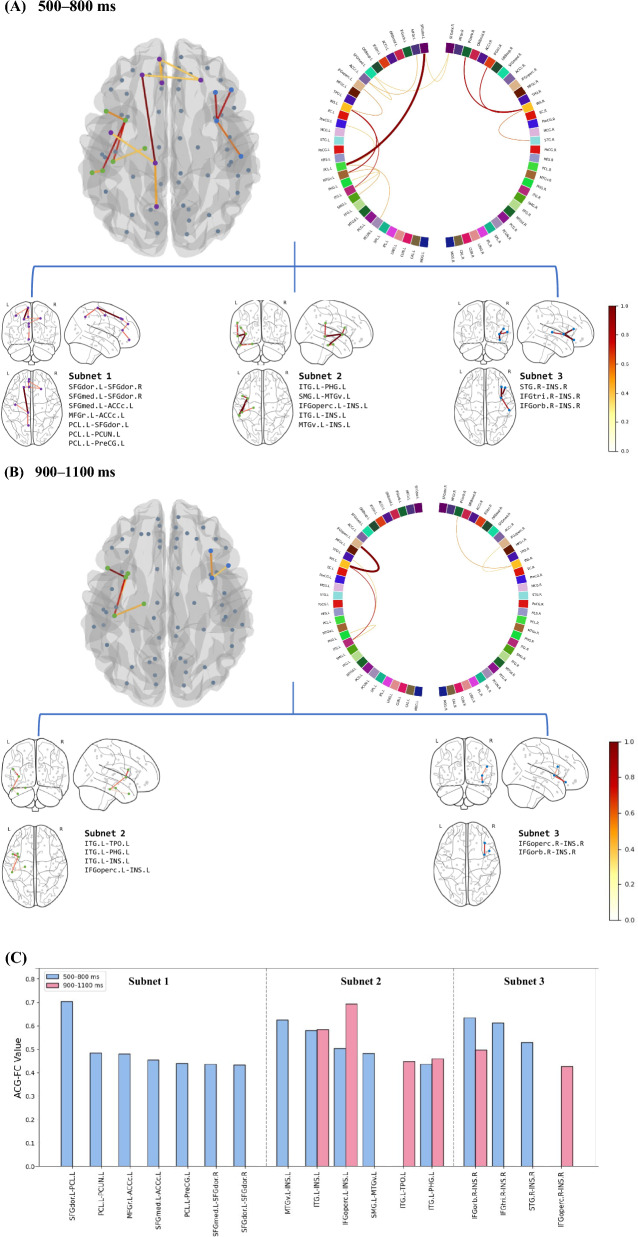
Common ACG-FCs within Two Windows. *Note.* The green dots in the 3D and brain images illustrate the volumetric centers of brain regions. In both anatomical and circular representations, the color depth and line thickness of ACG-FCs are proportional to their values. **A** Within 500–800 ms post-cue, ACG-FCs formed three interconnected subnetworks, distributed across the frontal and bilateral temporoparietal lobes. **B** Within 900–1100 ms post-cue, ACG-FCs formed two independent subnetworks in the left temporoparietal lobe and right frontal lobe. **C** ACG-FCs were grouped according to their affiliated subnetworks and separated by vertical dashed lines, with blue/red bars indicating ACG-FC values at 500–800 ms /900–1100 ms

AP II was utilized to conduct within-population attribution analyses under the 'switch' or 'repeat' condition, aiming to determine connectivity differences between distinct proactive control modes. As exhibited in the non-parametric *p* matrix in Fig. 9, connections with significant ACG-FC-value differences (FDR-corrected *p* < 0.050) were regarded as Differential ACG-FCs, which contributed more to distinguishing one specific proactive pattern from the other. The histograms in Fig. 9 illustrate the distribution of significance levels for ACG-FC-value differences. More Differential ACG-FCs contributed differently to discerning distinct task-switching patterns in the 500–800 ms window than in the 900–1100 ms window, with a 7:3 ratio (Fig. 10).

**Fig. 11:**
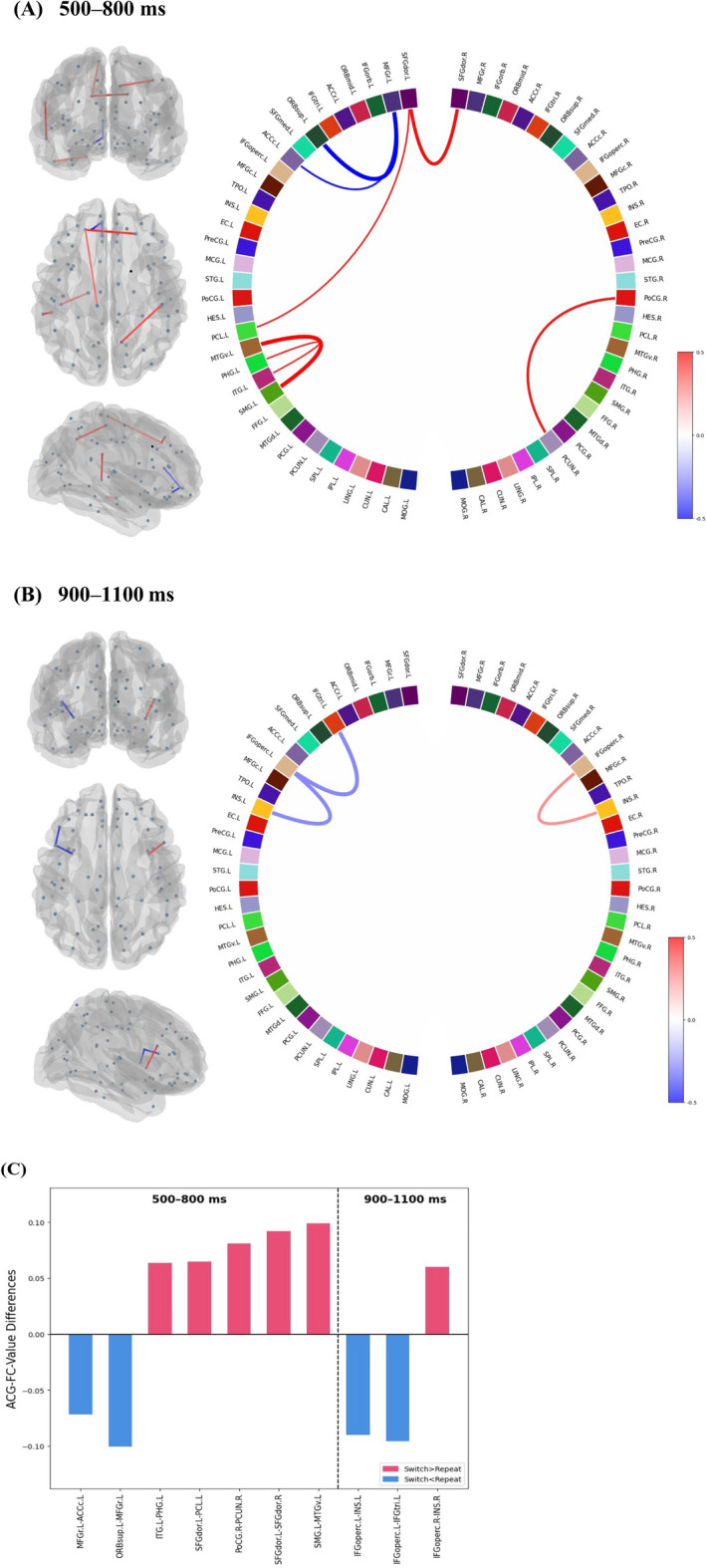
Differential ACG-FCs between ‘switch’ and ‘repeat’ conditions across two post-cue time windows. *Note.* The green dots on the 3D brain images represent the volumetric centers of brain regions. The Differential ACG-FCs between the 'switch' and 'repeat' populations are displayed separately in the anatomical and circular representations. Red denotes greater ACG-FC values for the 'switch' cue relative to the 'repeat' cue, whereas blue indicates the opposite. The color intensity and line thickness of Differential ACG-FCs are proportional to the magnitude of ACG-FC-value differences. **A** During the 500–800 ms window, seven connections in the frontoparietal and temporoparietal lobes were identified as Differential ACG-FCs, as displayed in the left side of the bar chart (**C**). **B** Within the 900–1100 ms post-cue, three connections in the temporoparietal lobes were identified as Differential ACG-FCs, as presented in the right side of the bar chart (**C**). Red indicates connections with greater ACG-FC values in the 'switch' condition, while blue represents the opposite

Figure 11 illustrates ACG-FCs showing functional distinctions between 'switch' and 'repeat' proactive control across two windows. During the 500–800 ms window, higher ACG-FC values for 'switch' proactive control were primarily distributed across the left frontoparietal system, encompassing connections between the left and right SFGdor, the left SFGdor and PCL, as well as the left postcentral gyrus (PoCG) and PCUN. Conversely, higher ACG-FC values for 'repeat' proactive control were primarily located in the left parietal lobe, involving connections between the left orbital superior frontal gyrus (ORBsup) and MFGr, and the left MFGr and ACCc. The positive functional difference ('switch > repeat,’ red edges in Fig. 11A) was highest in the ACG-FC between the left and right SFGdor, reaching 0.101, while the highest negative functional difference (‘repeat > switch,’ blue edges in Fig. 11A) was -0.096 in the ACG-FC between the left ORBsup and MFGr. As time progressed in the 900–1100 ms window, fewer ACG-FCs exhibited functional differences. Only the ACG-FC between the right IFGoperc and INS displayed increased ACG-FC value for 'switch' proactive control. The connections between the left IFGoperc and IFGtri, and the left IFGoperc and MTGv, contributed more to 'repeat' proactive control.

### Topological similarity across (ACG-)FC networks

**Fig. 12:**
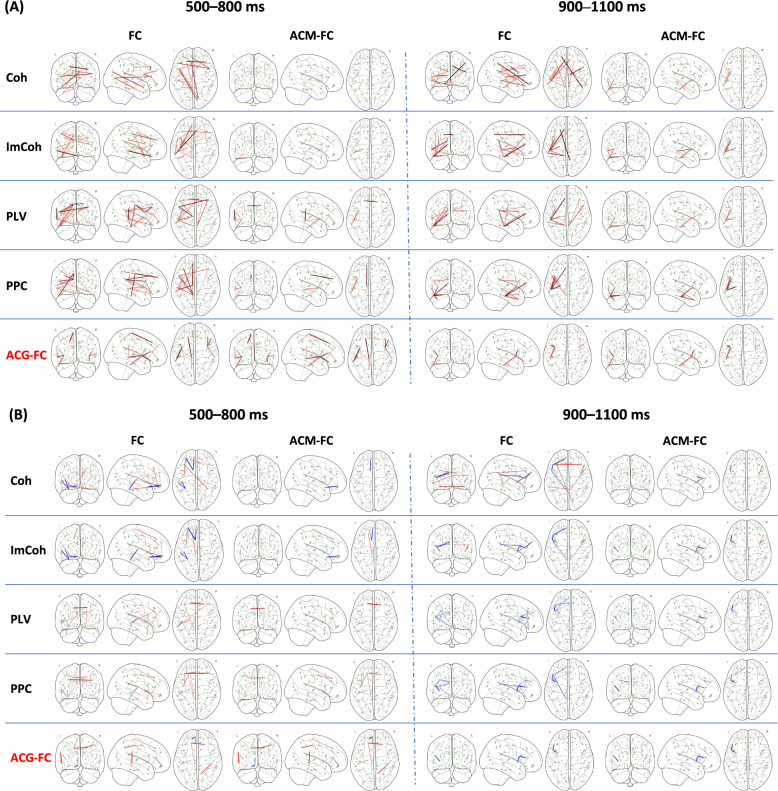
Comparison of (ACG-)FC Network Structures during Proactive Task-Switching. *Note.* Brain images contrast ACG-FC with phase-synchronized (PLV, PPC) and coherence-based (Coh, ImCoh) FC metrics for (**A**) common and (**B**) differential network structures. **A** Common connections are represented by red lines, with variable width and color intensity proportional to their extents of functional relevance during proactive preparation. **B** Red lines denote differential connectivity more functionally associated with the 'switch' condition, while blue lines indicate the reverse pattern

Figure 12 depicts the similarities and differences between the ACG-FC method and FC metrics in identifying common and differential connection structures during proactive task-switching. Overall, FC metrics, particularly Coh and PLV, identified more connections at an FDR-corrected significance level of 0.050. These FCs were distributed in anatomical locations similar to those of ACG-FCs but did not exactly correspond to specific connections. Instead, they tended to cluster and form an interconnected network unlike the discrete subnetworks observed in ACG-FCs. By re-expressing FC networks on the set of structurally reachable edges, we observed that ACG-FCs exhibit high coverage within the same time window: most significant ACM-FCs identified by different FC metrics are either directly included in the ACG-FCs or show consistent patterns at the level of shared key ROIs or nearby anatomical neighborhoods. This indicates that ACG-FCs share a broadly similar topological backbone with structurally reachable, direct inter-regional functional coordination. The list of ACM-FCs is summarized in Supplementary Table 4.

Conventional metrics revealed a greater number of common FCs with pronounced radial connection patterns during the 500–800 ms window compared to the 900–1100 ms window. Similar to Common ACG-FCs, these FCs were predominantly localized in the frontoparietal regions and bilateral frontotemporal junctions within 500–800 ms. However, unlike ACG-FC, which entirely lost prefrontal connections, FC exhibited weakened frontal connections at 900–1100 ms. All FC metrics indicated interhemispheric interactions or parallel processing, except for PPC, which lacked right-hemisphere FCs at 900–1100 ms post-cue. During proactive task-switching, the network structure of ACG-FCs more closely resembled those of phase-synchronized FCs, particularly the PLV metric at 500–800 ms (JS = 0.172, SPD = 0.860, FD = 6.928) and the PPC metric at 900–1100 ms (JS = 0.400, SPD = 0.759, FD = 3.464), as summarized in Table 1.

**Table 1:** Network structure similarity between ACG-FC and FC metrics during proactive task-switching

FC metric and category	500–800 ms						900–1100 ms					
	Common FCs			Differential FCs			Common FCs			Differential FCs		
Coherence-based FC	JS	SPD	FD	JS	SPD	FD	JS	SPD	FD	JS	SPD	FD
Coh	0.000	0.340	8.246	0.031	0.554	7.874	0.059	0.382	5.657	0.000	0.803	6.000
ImCoh	0.056	0.408	8.246	0.071	0.552	7.211	0.143	0.718	4.899	0.154	1.013	4.690
Phase-Based FC												
PLV	0.172	0.860	6.928	0.065	0.535	7.616	0.154	0.843	4.690	0.083	1.158	4.690
PPC	0.097	0.729	7.483	0.148	0.426	6.782	0.400	0.759	3.464	0.091	0.986	4.472

During proactive preparation, the structural similarity between ACG-FC and FC metrics is quantified in the 500–800 ms and 900–1100 ms windows under different analytical objectives

In terms of the differential graph-structure pattern at 500–800 ms, coherence- and phase-based FCs exhibited significant differences. Differential FCs identified by Coh and ImCoh were concentrated in the left prefrontal lobe. The FCs more strongly associated with 'repeat' proactive control were primarily localized within the left temporal and prefrontal areas, partially overlapping with the distribution of corresponding Differential ACG-FCs. By contrast, differential FCs derived from PLV and PPC were primarily distributed within the frontoparietal interconnected structure, linking the symmetric frontal gyri. The FCs more relevant to 'switch' proactive control were exclusively localized within the left temporal lobe, closely aligning with switch-related ACG-FCs. However, the right parietal ACG-FC of 'PoCG–PCUN,' functionally more associated with 'switch' proactive control, was absent across all FC metrics. During the 900–1100 ms window, all FC metrics, including ACG-FC, within the differential graph-structure pattern identified left frontoparietal connections with stronger functional relevance to 'repeat' proactive control, although the specific regional connections were not perfectly aligned. Furthermore, compared to the two phase-synchronized metrics, Coh and ImCoh also revealed more switch-related connections near the right inferior frontal and posterior cingulate regions, partially overlapping with Differential ACG-FCs in the right prefrontal lobe. Topologically, Differential ACG-FCs in the 500–800 ms window showed greater similarity to PPC-measured FCs (JS = 0.148, SPD = 0.426, FD = 6.782), whereas those during the 900–1100 ms window corresponded more closely with ImCoh (JS = 0.154, SPD = 1.013, FD = 4.690).

**Fig. 13:**
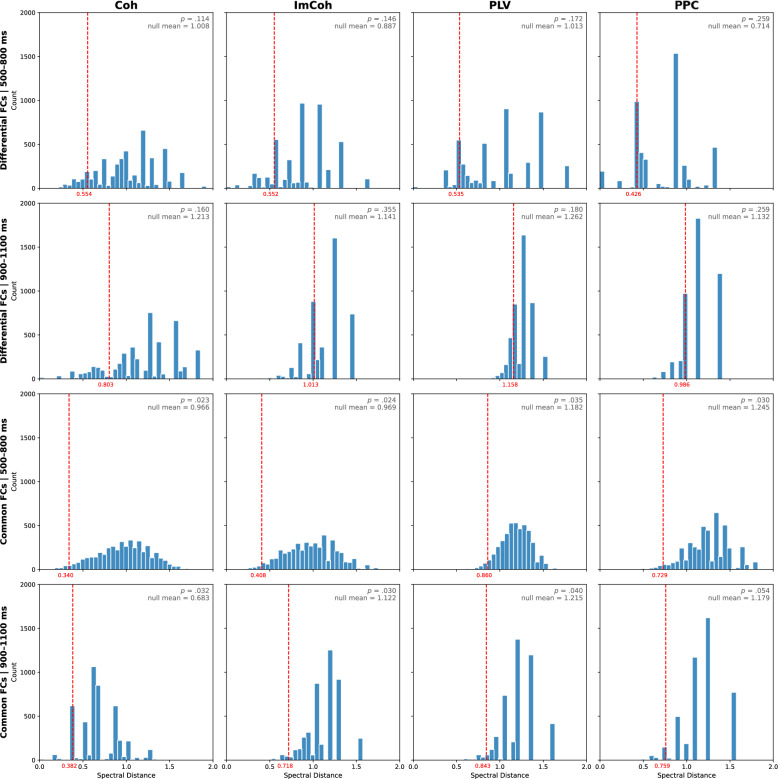
Non-parametric Test of SPDs between ACG-FC and FC Networks Using a Random-Network Null. *Note.* For each temporal window and connectivity type, 5000 surrogate networks were generated, each constrained to have the same number of edges as the corresponding ACG-FC network. Their SPDs to the FC networks were computed to form the null distribution, with its mean shown in the upper-right corner of each subplot. The vertical red line represents the SPD between the ACG-FC and FC networks, and the result of the one-sided non-parametric test against the null distribution is also in the upper-right corner

The statistical significance of the convergence between network structures formed by Differential or Common ACG-FCs and those formed by the corresponding Differential or Common FCs was assessed using SPD, based on a null distribution of SPD values generated from 5,000 random networks containing the same number of edges as the corresponding ACG-FC networks. The results are shown in Fig. 13. Because the number of Differential ACG-FCs was small and spatially dispersed (Fig. 11), the resulting networks were sparse and did not form canonical patterns. Spectral degeneracy across a large set of sparse adjacency matrices with identical edge counts led to identical eigenvalue spectra (i.e., isospectrality), resulting in equidistant distances relative to the target FC networks, as illustrated by the null SPD distributions for Differential FCs in Fig. 13. Similarly, because only six Common ACG-FCs were identified during the 900–1100 ms window, the randomly generated sparse matrices also exhibited spectral degeneracy, producing equidistant SPD distributions with respect to the Common FC networks. Although the prevalence of equidistant SPD values prevented the similarity between the ACG-FC networks and the Differential FC networks from reaching statistical significance under non-parametric testing (p = 0.114–0.355), the observed SPDs nevertheless fell within the lower quartile of the corresponding null distributions and were 8.2–47.2% lower than the null means. Encouragingly, the Common ACG-FC networks exhibited higher structural similarity to the Common FC networks across all FC metrics, reaching marginal-to-significant levels (*p* = 0.030–0.054) and showing SPDs that were 27.3–64.8% lower than the corresponding null means. In contrast, during the 500–800 ms window, 15 Common ACG-FCs were identified (Fig. 10). Under this condition, the null SPD distributions did not exhibit equidistance effects, and the SPDs between the Common ACG-FC network and each Common FC network were statistically significant in one-sided tests (p = 0.023–0.035), with values 30.6–44.0% lower than the corresponding null means.

### Statistical correlation between identified (ACG-)FCs and behavioral performance

**Fig. 14:**
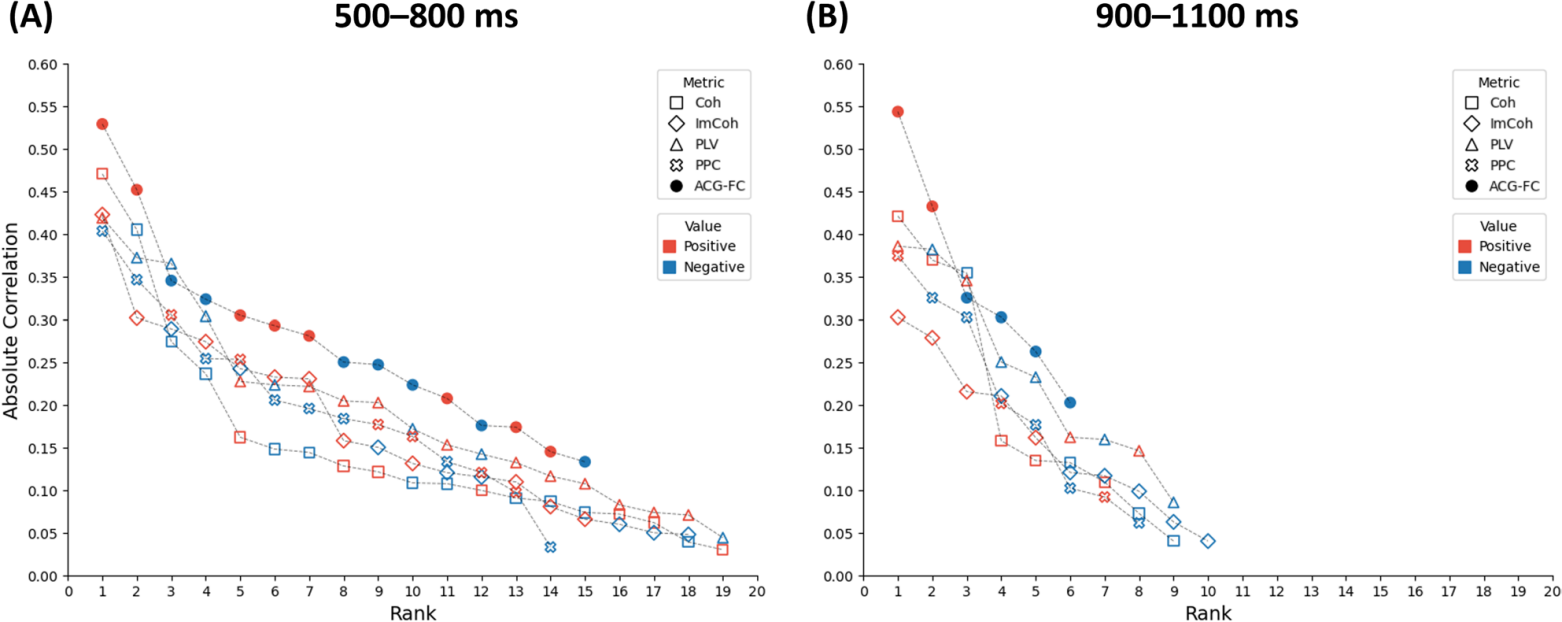
Rank of absolute correlations between common FCs and behavioral RTs across distinct metrics. *Note.* For each (ACG-)FC metric (indicated by different dot shapes), the significant common connections identified in the 500–800 ms (**A**) and 900–1100 ms (**B**) windows are ranked in descending order by the absolute value of the correlation between their connectivity and the first-target RTs. Red/blue denotes positive/negative correlations

The comparison of common and Differential ACG-FCs with existing FC metrics reflected their topological similarity, while their statistical correlations with behavioral performance further highlighted their functional relevance to proactive preparation. As shown in Fig. 14, the absolute correlations of common connections identified by different (ACG-)FC metrics were ranked in descending order. During the 500–800 ms proactive task-switching period, the ranked correlations exhibited a sharp decline until approximately the top 3–6 links, followed by a gradual plateau. By contrast, during the 900–1100 ms target anticipation period, fewer connections exhibited a wider range of correlation, with a consistently steep downward trend from beginning to end. Across nearly all ranks, Common ACG-FCs maintained higher correlations, with the strongest correlations observed for ‘SFGdor.L–PCL.L’ (*r* = 0.529) within 500–800 ms and ‘IFGoperc.L–INS.L’ (*r* = 0.543) within 900–1100 ms. Within 500–800 ms, the lowest-ranked 15th link ‘SFGdor.L–SFGdor.R’ yielded a negative correlation of.202, which corresponded to links ranked 5th–12th in FC metrics. Within 900–1100 ms, the lowest-ranked 6th link ‘IFGoperc.R–INS.R’ had a negative correlation of 0.133, a value comparable to the 1st–3rd ranked links in FC metrics.

**Fig. 15:**
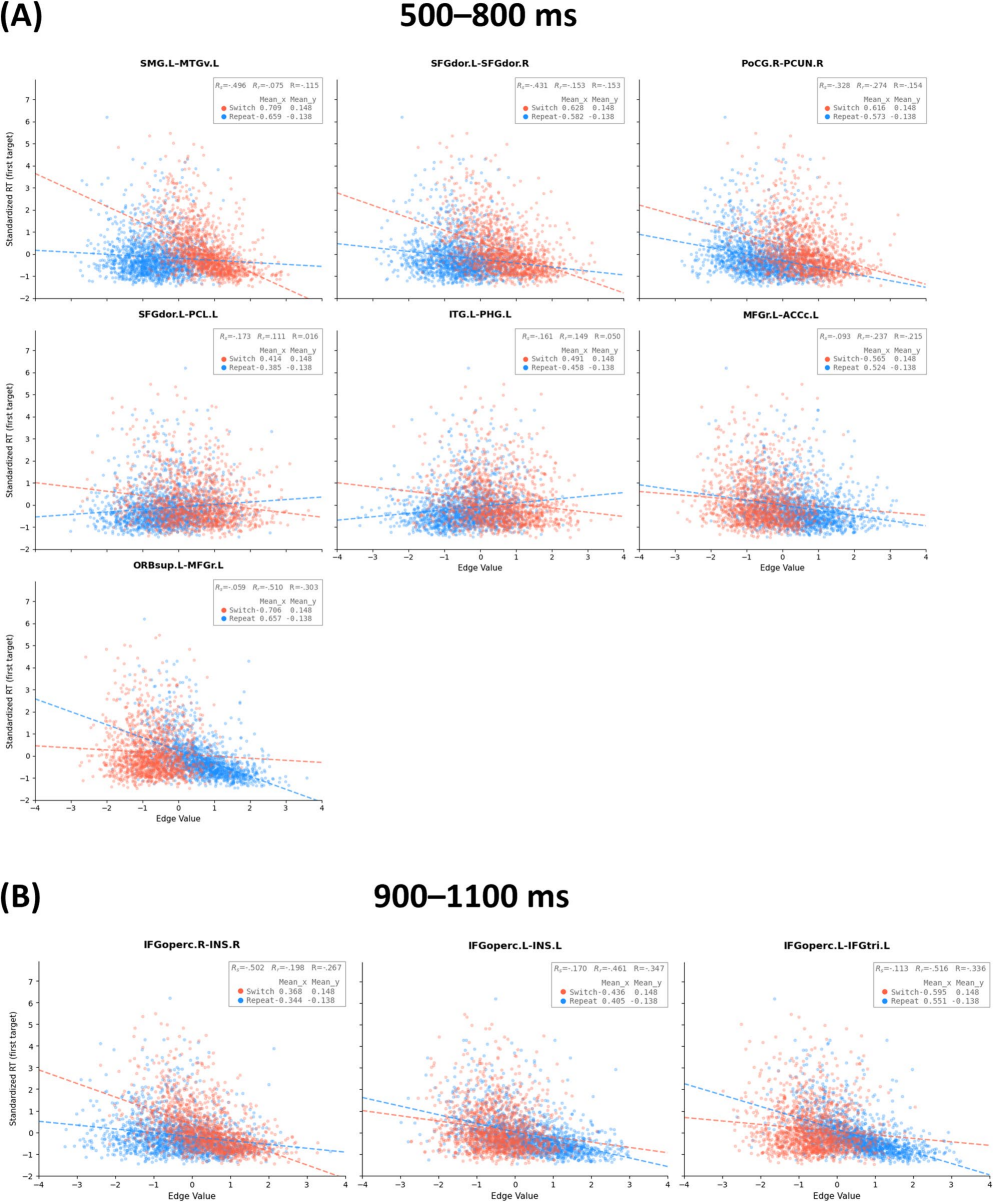
Connectivity differences of differential ACG-FCs between ‘switch’ and ‘repeat’ trials. *Note.* Significant Differential ACG-FCs identified within 500–800 ms (**A**) and 900–1100 ms (**B**) are shown with standardized connectivity for ‘switch’ (blue dots) and ‘repeat’ (red dots) trials, plotted against the standardized RT of the first target (y-axis). Correlations with first-target RT in switch, repeat, and all trials are reported in the upper-right corner of each subplot. The blue and red dashed lines denote the regression fits of connectivity to behavioral performance for trials in the repeat and switch conditions, respectively

Significant Differential ACG-FCs were standardized to trial-wise z-scores, as shown in Fig. 15. Within 500–800 ms, ‘ORBsup.L–MFGr.L’ and ‘MFGr.L–ACCc.L’ exhibited remarkably higher connectivity in ‘repeat’ trials. ‘ORBsup.L–MFGr.L’ during ‘repeat’ trials showed the strongest correlation with standardized RTs of the first target (*r* = –0.510), while ‘SMG.L–MTGv.L’ during ‘switch’ trials demonstrated the highest correlation (*r* = –0.496). Notably, the ACG-FC value of ‘SFGdor.L–PCL.L’ and ‘ITG.L–PHG.L’ in ‘repeat’ trials displayed positive correlations with the first-target RT, opposite to the pattern observed in ‘switch’ trials. Within 900–1100 ms, ‘IFGoperc.L–INS.L’ and ‘IFGoperc.L–IFGtri.L’ showed stronger connectivity in ‘repeat’ trials, with correlations of –0.516 and –0.461 with the first-target RT, respectively, while ‘IFGoperc.R–INS.R’ exhibited greater connectivity in ‘switch’ trials and achieved a negative correlation of –0.502. Results of FC metrics are presented in Supplementary Fig. 4.

To further examine the extent to which (ACG-)FC findings explained variability in task-switching behavioral performance, we conducted stepwise regression analyses using z-scores of differential (ACG-)FCs—taken from all trials of the condition where their connectivity was significantly stronger than the opposite condition—as dependent variables, with the corresponding RTs of the first target as behavioral criteria. Except for PPC and PLV during the 900–1100 ms window of ‘switch’ trials, where no stronger FCs relative to ‘repeat’ trials were detected and thus no models were constructed, results of stepwise regression models based on other FC metrics are shown in Table 2.

**Table 2:** Stepwise regression of differential FCs on behavioral RT in ‘switch’ and ‘repeat’ trials across metrics

Duration	‘Repeat’ trials							‘Switch’ trials						
Metric	Model	Variables	*β*	*p*	SEE	*F*	Adj *R*^*2*^	Model	Variables	*β*	*p*	SEE	*F*	Adj *R*^*2*^
500–800 ms														
Coh	1	EC.L-ORBmid.L	− 0.373	0.000	0.810	378.508	0.197	1	ORBmid.L-INS.L	− 0.335	0.000	1.035	158.889	0.101
	2	EC.L-ORBmid.L	− 0.252	0.000	0.780	264.781	0.239	2	ORBmid.L-INS.L	− 0.290	0.000	0.985	161.619	0.180
		EC.L-MFGr.L	− 0.229	0.000					IFGorb.L-FFG.R	− 0.282	0.000			
								3	ORBmid.L-INS.L	− 0.281	0.000	0.947	155.148	0.242
									IFGorb.L-FFG.R	− 0.261	0.000			
									ORBsup.L-SFGdor.R	− 0.152	0.000			
ImCoh	1	EC.L-ORBmid.L	− 0.379	0.000	0.807	392.016	0.205	1	ORBmid.L-INS.L	− 0.363	0.000	1.028	180.828	0.113
	2	EC.L-ORBmid.L	− 0.297	0.000	0.777	272.521	0.250	2	ORBmid.L-INS.L	− 0.333	0.000	0.972	185.013	0.204
		EC.L-MFGr.L	− 0.142	0.000					SFGdor.L-PCL.L	− 0.262	0.000			
								3	ORBmid.L-INS.L	− 0.231	0.000	0.936	202.935	0.263
									SFGdor.L-PCL.L	− 0.216	0.000			
									IFGorb.L-FFG.R	− 0.077	0.001			
PPC	1	FFG.L-STG.L	− 0.326	0.000	0.844	229.899	0.128	1	MFGc.L-MFGc.R	− 0.554	0.000	0.926	549.332	0.260
	2	FFG.L-STG.L	− 0.328	0.000	0.808	194.485	0.202	2	MFGc.L-MFGc.R	− 0.529	0.000	0.925	277.293	0.282
		HES.L-MTGv.L	− 0.239	0.000					SFGdor.L-SFGdor.R	− 0.052	0.024			
PLV	1	HES.L-MTGv.L	− 0.347	0.000	0.835	268.383	0.147	1	SFGdor.L-SFGdor.R	− 0.504	0.000	0.926	489.253	0.257
	2	HES.L-MTGv.L	− 0.350	0.000	0.797	222.566	0.225							
		FFG.L-HES.L	− 0.136	0.000										
ACG-FC	1	ORBsup.L-MFGr.L	− 0.452	0.000	0.780	527.341	0.260	1	SMG.L-MTGv.L	− 0.531	0.000	0.950	449.005	0.244
	2	ORBsup.L-MFGr.L	− 0.390	0.000	0.770	290.658	0.277	2	SFGdor.L-SFGdor.R	− 0.475	0.000	0.906	268.942	0.317
		MFGr.L-ACCc.L	− 0.133	0.002					SMG.L-MTGv.L	− 0.108	0.000			
900–1100 ms														
Coh	1	IFGtri.L-SFGmed.L	− 0.411	0.000	0.800	425.786	0.216	1	IFGorb.L-IFGorb.R	− 0.372	0.000	1.021	200.692	0.124
	2	IFGtri.L-SFGmed.L	− 0.346	0.000	0.779	266.652	0.259	2	IFGorb.L-IFGorb.R	− 0.313	0.000	0.955	215.588	0.233
		IFGoperc.L-IFGtri.L	− 0.134	0.000					IFGtri.R-INS.R	− 0.283	0.000			
	3	IFGtri.L-SFGmed.L	− 0.312	0.000	0.775	185.678	0.268							
		IFGoperc.L-PCG.L	− 0.128	0.000										
		IFGoperc.L-IFGtri.L	− 0.102	0.000										
ImCoh	1	IFGoperc.L-IFGtri.L	− 0.386	0.000	0.806	399.476	0.206	1	IFGoperc.R-INS.R	− 0.360	0.000	1.027	181.701	0.114
	2	IFGoperc.L-IFGtri.L	− 0.361	0.000	0.763	309.551	0.272	2	IFGoperc.R-INS.R	− 0.363	0.000	0.990	152.045	0.178
		IFGtri.L-SFGmed.L	− 0.121	0.000					IFGtri.R-INS.R	− 0.172	0.000			
	3	IFGoperc.L-IFGtri.L	− 0.322	0.000	0.762	209.499	0.295							
		IFGoperc.L-INS.L	− 0.151	0.000										
		IFGtri.L-SFGmed.L	− 0.064	0.008										
PPC	1	IFGoperc.L-INS.L	− 0.433	0.000	0.792	465.328	0.234							
	2	IFGoperc.L-INS.L	− 0.316	0.000	0.755	334.562	0.306							
		IFGoperc.L-IFGtri.L	− 0.248	0.000										
	3	IFGoperc.L-INS.L	− 0.289	0.000	0.746	239.804	0.322							
		IFGoperc.L-IFGtri.L	− 0.215	0.000										
		MFGc.L-SFGmed.L	− 0.114	0.000										
PLV	1	IFGoperc.L-IFGtri.L	− 0.402	0.000	0.796	445.324	0.226							
	2	IFGoperc.L-IFGtri.L	− 0.368	0.000	0.788	245.304	0.244							
		IFGoperc.L-INS.L	− 0.116	0.000										
	3	IFGoperc.L-IFGtri.L	− 0.352	0.000	0.784	169.440	0.257							
		IFGoperc.L-INS.L	− 0.095	0.000										
		MFGc.L-ACCr.R	− 0.074	0.000										
ACG-FC	1	IFGoperc.L-IFGtri.L	− 0.451	0.000	0.777	541.904	0.266	1	IFGoperc.R-INS.R	− 0.583	0.000	0.944	473.427	0.252
	2	IFGoperc.L-IFGtri.L	− 0.328	0.000	0.733	399.590	0.345							
		IFGoperc.L-INS.L	− 0.218	0.000										

During the 500–800 ms and 900–1100 ms windows, stepwise regression models were fitted separately for ‘switch’ and ‘repeat’ trials, relating the significant differential (ACG-)FCs identified by each metric to the standardized RT of the first target trial (y-axis). Connectivity and behavioral RTs were normalized to z-scores. During the 900–1100 ms window of ‘switch’ trials, no FCs showed higher connectivity than those in ‘repeat’ trials under PPC or PLV; therefore, the models were absent

Within 500–800 ms, the ACG-FC-based regression in the ‘repeat’ condition included two connections (i.e., ‘ORBsup.L–MFGr.L,’ *β* = –0.452, *p* < 0.001 in model 1; and *β* = –0.390, *p* < 0.001 in model 2; and ‘MFGr.L–ACCc.L,’ *β* = –0.133, *p* = 0.002 in model 2), which jointly explained approximately 27.7% of the variability in RTs. In the ‘switch’ condition, one link was included (i.e., ‘SMG.L–MTGv.L,’ *β* = –0.531, *p* < 0.001 in model 1; and *β* = –0.108, *p* < 0.001 in model 2; and ‘SFGdor.L–SFGdor.R,’ *β* = –0.475, *p* < 0.001 in model 2), jointly explaining over 31.7% of the variability in RTs. By contrast, regression models based on other FCs typically included 1–3 differential links, with adjusted *R*^*2*^ of 0.202–0.250 for ‘repeat’ trials and 0.242–0.282 for ‘switch’ trials.

Within 900–1100 ms, the ACG-FC-based regression in the ‘repeat’ condition included two links (i.e., ‘IFGoperc.L–IFGtri.L,’ *β* = –0.451, *p* < 0.001 in model 1; and *β* = –0.328, *p* < 0.001 in model 2; and ‘IFGoperc.L–INS.L,’ *β* = –0.218, *p* < 0.001 in model 2), jointly explaining about 34.5% of the variability in RTs. In the ‘switch’ condition, one link was included (i.e., ‘IFGoperc.R–INS.R,’ *β* = –0.583, *p* < 0.001 in model 1), explaining over 25.2% of the variability in RTs. By contrast, regression models based on other FCs generally included 1–3 differential links, yielding adjusted *R*^*2*^ of 0.257–0.322 for ‘repeat’ trials and 0.178–0.233 for ‘switch’ trials.

## Discussion

Our behavioral analysis results were comparable to those of previous studies using similar task-switching paradigms with EEG measurements: compared to 'repeat' cues, target responses following 'switch' cues exhibited slower RTs and higher ERs, indicating switch costs during reactive execution [6, 29, 86]. These behavioral differences gradually diminished over consecutive trials following the cue. Additionally, the present study identified EEG signal differences elicited by distinct proactive controls and examined their associations with neural interactions to investigate the substrates underlying proactive task adaptation.

### EEG signal analysis

GFP analysis revealed no significant differences in total EEG power effects between 'switch' and 'repeat' cues during the P3 latency window, possibly because neural activity at this stage remains engaged in cue perception rather than in cognitively differentiated higher-order episodic control. In contrast, during the late-latency period including non-phase-locked ERPs, variations in total EEG power decomposition were observed between two task-switching modes. This suggests that proactive control not only simply activates S–R pathways but also involves complex neural activity patterns underlying collaborative interactions. Specifically, the potential differences emerged in two late-latency windows: 500–800 ms and 900–1100 ms, which correspond to endogenous LPC and CNV components, respectively. The low-frequency LPC typically reflects contextual effects associated with the involvement of the frontoparietal cortex in early switch-related preparatory activity [15]. Sustained CNV is associated with anticipation and incorporates switch-related contextual effects as well [43]. These activities in two windows suggest that the proactive update of S–R mapping and anticipation of target tasks occurs as two distinct sequential processes.

### Model performance comparison

Due to the fixed spatial arrangement in structured data, the convolution kernel size determines the neighborhood range and connection pattern through which adjacent signal features engage in information interaction [135], as shown in Supplementary Fig. 1A. Given that the brain is an organ with a complex neurophysiological connectome, in which neural activity across various regions exhibits spatial relationships, determining the optimal ordering of the regional dimension remains challenging. Graph convolution introduces an actual connectome that enables long-distance interactions between distant regions while simultaneously eliminating redundant interactions between neighboring regions. This methodology could be regarded as efficient data regularization via graph convolution within the structural network, serving as a signal filter to reduce redundancy in feature interaction and aggregation during signal extraction. Additionally, due to the graph structure, modeling is independent of specific node arrangements, thereby preserving permutation invariance so that the actual spatial structure, properties, and interactions remain invariant regardless of node arrangement [20].

To obtain structurally relevant functional representations, an alternative pipeline is to first estimate individual-level FC, then sparsify it via an anatomical template, and use the resulting matrix as a high-dimensional feature set for cross-condition classification. This workflow is intuitive, but constructing FC as a feature-engineering step may introduce information loss and variability and can propagate errors to downstream models. In contrast, our approach treats structural connectivity as a pathway prior within an RGNN, guiding graph convolution and feature integration during end-to-end task optimization, so that the model learns discriminative interaction features adaptively from the cortical signals. Accordingly, the structural prior is not a post-hoc mask but an integral part of the learning process and—together with attribution analysis—supports interpretability at the connection level.

By incorporating DTI connections into EEG signals, RGNN compresses both the dynamic signal features of individual regions and inter-regional relationships. The remarkable performance of RGNNs indicates that representing inversely traced EEG signals as graph sequences enriched with spatial information enables superior pattern recognition compared with MAR, ML, and DNN models that process signal sequences without group-level structural priors. The T-GCN model maximized proactive-control-mode recognition rate across all cue-locked epochs (over 0.800), demonstrating its effectiveness in tracking the neural activity trends underlying signals; thus, it was used to infer ACG-FC values for group-level connective features to quantify their functional relevance with proactive task-switching in this study. In the future, if subject-specific connectomes derived from DTI replace the group-level connectome as the model input, the recognition accuracy of RGNN models in distinguishing proactive control types is expected to improve further.

### ACG-FCs in task-switching preparation

To establish a functional correlation with the cognitive activity of interest, data-driven methods, such as FC or psychophysiological interactions (PPI), require additional linear regression modeling, which uses behavioral performance as the evaluative criterion to single out highly correlated significant connections. As their anatomy-guided support and extension, the ACG-FC method determines the functional relevance of neural connections to cognitive activity by evaluating how interactions among node signal-sequence features, constrained by DTI-based structural connectome priors, contribute to functional recognition in RGNNs. Supervised by true cues, node features undergo multiple iterations of message passing and state updates until their hidden states adequately capture critical pathways for signal feature interaction (Fig. 3), thereby presenting nonlinear associations beyond signal synchrony.

This present study proposes two attribution strategies based on the IG algorithm, namely AP I and II, assigning distinct connectivity implications to connective features. AP I attributes samples from both cues collectively, emphasizing Common ACG-FCs crucial for proactive task-switching. In contrast, AP II separates samples from the 'switch' and 'repeat' cues to identify ACG-FCs with significantly different ACG-FC values, considering them as distinctions in the neural mechanism between two proactive control modes.

#### Common ACG-FCs

##### During the 500–800 ms window post-cue

AP I identified multiple ACG-FCs, which significantly contributed to distinguishing proactive control modes within the frontoparietal and temporoparietal cortices. These ACG-FCs form three independent subnetworks (Subnets 1–3 in Fig. 10A). Through inter-regional functional coordination within three complementary ACG-FC subnetworks clustered around the frontoparietal “multiple demand” system, reactive responses characterized by execution inertia are transformed into voluntarily controlled execution preparation. This supports the existence of larger-scale brain systems involved in cognitive control, such as top-down attentional control, task-set maintenance, implementation, and inhibition control [40, 99].

Subnet 1 is mainly situated in the left frontal and parietal lobes but also connects both hemispheres through two ACG-FCs linking the left SFGdor and SFGmed to the right SFGdor. The dorsolateral and medial parts of the SFG, together with the frontopolar MFGr, initiate proactive control in multitasking by selecting, encoding, and maintaining abstract rules [19]. Subnet 1 also integrates regions adjacent to the frontoparietal cortex, including the left PreCG, PCUN, and PCL. These regions have been widely reported to participate in S–R mapping transitions through neural activation and inter-regional interactions to meet task-switching demands [18, 79].

Within Subnet 1, the left SFGdor acts as a hub to link other regions through ACG-FCs, including connections with the left SFGmed, the right SFGdor, and the left PCL. These inter-regional interactions within the PFC primarily support goal-directed behavior, encompassing decision-making, voluntary shifts of attentional focus, and cognitive resource reallocation [8, 66]. The left PCL connects the left PreCG and the left PCUN via ACG-FCs, likely serving voluntary attention control in motor-sensory processing [4] by regulating attention and synchronizing internal intentions with motor responses. Compared with the PLV-based FCs reported during the late latency window [79], the major difference arises from the SFG-centered decision network connecting the ACCc responsible for conflict-resolving and several parietal regions responsible for attentional regulation. Among these, the ‘SFGdor.L–PCL.L’ connection exhibits the highest ACG-FC value within 500–800 ms, highlighting its significance in coordinating frontoparietal executive functions during proactive task-switching. This SFG-centered decision chain—spanning from conflict detection to attentional regulation—represents a key finding relative to conventional FC.

Overall, Subnet 1 in the frontoparietal lobe, as the initiator of proactive control, engages in cue-induced conflict detection and autonomously allocates attentional resources toward endogenous goals to form intentional decisions regarding the maintenance and inhibition of S–R mappings. Overall, Subnet 1, known as the frontoparietal executive network, is responsible for detecting conflicts based on contextual cues and reallocating attentional resources toward goal-relevant information to reset the target task. It extends the attention-modulation FC network found by López et al. [79], which links parietal regions such as the PCUN and PoCG through the PCL in the sensorimotor cortex, toward the prefrontal executive control network (ECN) and alerting network represented by the SFG and ACCc, respectively, which are responsible for decision-making and conflict detection. The ECN and alerting network jointly promote flexible allocation of attention resources, enhancing the processing of endogenous target demands while suppressing distractions from irrelevant information [48]. This extended antagonistic feedforward regulation of attention resources shows strong validity consistency with proactive control [3, 81], supporting selective orientation toward active task sets and inhibition of inactive ones.

Subnet 2 primarily localized in the left temporoparietal and frontotemporal lobes. The temporoparietal lobe facilitates regional interactions supporting proactive S–R mapping transitions through cross-frequency coupling of neural oscillations [79]. Converging evidence suggests that categorical information is represented in the temporal cortex [134]. The ACG-FC of 'ITG.L–PHG.L' may facilitate the continuous encoding of task-relevant visual information in working memory to update S–R mapping. The ACG-FC of 'MTGv.L–SMG.L' may process contextual semantic information underlying cues and encode active target tasks into working memory. These connections also appeared in PLV-based FCs [79]; however, Subnet 2 complementally reveals that the left INS serves as a critical anatomical hub at the temporoparietal junction, mediating functional interactions among the ITG, IFGoperc, MTGv, and sensorimotor regions. During the late task-switching CTI, the INS is thought to facilitate task-set reconfiguration by mediating information exchange among functional regions associated with execution, sensation, and memory [39]. Therefore, within Subnet 2, it may act as a mediator for functional integration, providing neural pathways that coordinate interactions among other regions. Subnet 2 facilitates the representation of recent S–R mappings by integrating concrete information (such as shape and color) into working memory, thereby updating active task-related information.

Subnet 3 is situated in the right frontotemporal region and is nearly symmetrical to Subnet 2 across the cerebral midline. The effect of non-phase-locked EEG power in this area significantly affects the efficiency of proactive S–R mapping switching [86]. Neural activity in the right IFG is closely associated with inhibitory control. Its orbital part contributes to response inhibition of feature-independent visual stimuli [17, 123]. Therefore, the triangular and orbital parts of the right IFG are likely involved in suppressing interference from unrelated features. The connections among the above regions are consistent with PLV-based FCs [79]. However, the right anterior INS was complementally identified by the ACG-FC approach as an anatomical mediator linking other regions. It is considered a pivotal node of the salience network: when contextual conflicts arise, such as internal goal discrepancies or salient external stimuli, it interacts with the inferior frontal cortex to rapidly initiate the frontoparietal executive network for modifying current cognitive states or suppressing irrelevant information [119]. Subnet 3, through its interaction with other temporal regions such as the right STG via the INS neural pathways, likely plays a central role in concretizing inactive S–R mappings through contextual information and effectively suppressing distracting features irrelevant to targets. This process alleviates the working memory load, inhibits interference from irrelevant information in the workflow, enables the brain to terminate obsolete task sets, and allows focus on current task demands.

##### During the 900–1100 ms time window post-cue

In addition to the connectivity analysis of non-phase-locked power modulation of the switch-related LPC, we also performed ACG-FC analysis within the CNV latency window to capture differences in cue-induced EEG power effects. The task-switching CNV reflects the brain’s anticipatory regulation—psychologically anticipating upcoming targets and continuously adapting to new cognitive demands, thereby facilitating the transition from abstract S–R mapping to actual execution. To date, no studies have provided a detailed structure–function analy

sis of this process. The low-frequency neural signals gradually decrease during the 900–1100 ms window, indicating progressively reduced demand for updating the S–R mapping. Within this period, Subnet 1, responsible for initiating proactive control through cognitive resource modulation and intention management, disappears, while Subnets 2 and 3, which execute task-set reconfigurations, remain in the same location but with simplified network structures. This finding indicates that the late CTI no longer engages the PFC’s executive functions, such as goal setting or rule encoding, but instead retains two frontotemporal subnetworks to sustain the lower-level cognitive demands of maintaining resource allocation and task-set reconfiguration.

Subnet 2 is positioned near the left frontotemporal lobe. It likely functions as a successor in the execution function, specifically maintaining the restructured S–R mappings from the previous window. Through PHG-mediated interactions among frontotemporal regions, Subnet 2 ensures that front-end working memory reflects newly activated perception–action rules, harmonizing abstract S–R mappings with executed actions in reactive trials. It no longer needs to interpret or activate rule information; instead, it maintains the current task context and sustains focus on the current endogenous goal, thereby producing a simplified structure.

Subnet 3 is positioned near the right frontal lobe. Inferior frontal regions have been confirmed to involve the executive inhibition of impulsive responses elicited by working memory [88, 119], with the right part playing a decisive role compared to the left [5]. Subnet 3 may inherit and sustain executive functions by inhibiting inactive S–R mappings, minimizing interference from irrelevant features on internal goals and ensuring uninterrupted information flow in working memory as targets approach. As it no longer processes contextual semantics to determine inactive task information, its structure remains simplified.

#### Differential ACG-FCs

AP II attributed the functional differences between the two proactive control modes to structural connections. Attribution analysis was conducted separately for the 'repeat' and 'switch' proactive controls, to assess each connection’s contribution for distinct proactive control modes and define those with significant differences as Differential ACG-FCs. Differential ACG-FCs did not form systematic subnetworks but rather dispersed as local edge differences across the cortex. They represent functional differences in inter-regional interactions between the two proactive task-switching controls. Some are essential for a single mode (functional specificity), while others influence both yet differ significantly in their contribution (functional variability).

##### During the 500–800 ms time window post-cue

The ACG-FCs connecting the left MFGr with the ACCc and ORBsup exhibit greater functional relevance in the 'repeat' proactive control (Fig. 11A), with no significant difference from the regions involved in PLV-based differential FCs [79]. MFGr is responsible for attention regulation, while ORBsup is widely considered relevant to interference inhibition [85]. Given their collective influence on two patterns of proactive control, the synergistic function of this triadic structure, consisting of 'MFGr.L–ACCc.L' and 'MFGr.L–ORBsup.L', likely enhances attention maintenance and restrains attentional shift impulses, both of which are crucial for sustaining cognitive inertia. Since the 'switch' pattern imposes significantly lower demands on maintaining cognitive consistency and inhibiting task-irrelevant information, this structure is more functionally important for 'repeat' proactive control.

More ACG-FCs exhibit stronger functional relevance in proactively switching S–R mappings. Distinct from PLV-based differential FCs [79], the Differential ACG-FCs emphasized the decision-making function of PFC, forming a three-edge structure through the left SFGdor, left PCL and right SFGdor that connects the dorsal frontal cortex with the left posterior parietal cortex. Given that these connections were also identified as Common ACG-FCs for proactive task-switching, this trans-hemispheric structure is likely involved in directing attention toward task-relevant information to establish internal goals. Compared with the 'switch' proactive control that facilitates internal goal transitions [6], the 'repeat' condition imposes a markedly lower cognitive load on these ACG-FCs. As shown in Fig. 15, the first-target RT in 'repeat' trials is even weakly negatively correlated with the ACG-FC of 'SFGdor.L–PCL.L', implying that stronger impulse of 'switch' control may influence subsequent behavioral performance.

Moreover, two independent Differential ACG-FCs, 'ITG.L–PHG.L' and 'SMG.L–MTGv.L,' were present in Subnet 2 of the AP I results, exhibiting greater functional relevance to 'switch' proactive control. These two functional interactions in figure recognition and visual memory were likewise identified by PLV-based FC [79]. The collaborative function of 'ITG.L–PHG.L' and 'SMG.L–MTGv.L' possibly facilitates the integration of target-related multisensory information. Compared with the 'switch' context, the cognitive demands of 'repeat' proactive control for processing new task information and updating perception–action rules in working memory are markedly lower [6]. As shown in Fig. 15, even in 'repeat' trials, first-target RT exhibited a weak negative correlation with the ACG-FC of 'ITG.L–PHG.L', suggesting that a stronger drive to retrieve perception–action rules may influence subsequent behavioral performance.

The results from AP I lacked the Differential ACG-FC of 'PoCG.R–PCUN.R', suggesting its functional relevance specifically in the 'switch' context. This function-specific interaction has also been corroborated by López et al. [79]. During proactive task-switching, the 'PoCG.R–PCUN.R' may redirect attention to sensory information pertinent to the new task-set. As this process does not impose cognitive load in the 'repeat' condition, its influence is specific to the 'switch' proactive control.

##### During the 900–1100 ms time window post-cue

When approaching targets, no Differential ACG-FCs were detected in the frontoparietal cortex; only three Differential ACG-FCs were localized in the bilateral frontotemporal lobes (Fig. 11B).

The connections 'IFGoperc.L–INS.L' and 'IFGoperc.R–INS.R,' belonging to Subnet 2 and 3 respectively, maintain functional relevance in both 'repeat' and 'switch' conditions. Lesions in the right IFG impair the ability to suppress habitual responses or manage unexpected shifts, resulting in increased switch costs [87]. Conversely, left IFG damage causes greater restart costs, implicating difficulties in voluntarily maintaining the representation of internal goals [105]. These findings suggest that the left IFG is predominantly involved in maintaining goal-directed processes within task contexts [127], whereas the right IFG emphasizes inhibition of obsolete task-sets and attenuation of irrelevant perception–action pathways [119, 127]. Thus, during proactive task-switching, the interaction between the ‘IFGoperc.L–INS.L’ interaction during CNV latency primarily sustains current sensorimotor associations and attention focus on the active task-set, whereas the ‘IFGoperc.R–INS.R’ interaction emphasizes ongoing inhibition of reactive interference from competing task-sets, preventing attentional drift toward irrelevant features. The stronger interference induced by previously activated S–R mapping rules in 'switch' than in 'repeat' conditions highlights the cognitive load on 'IFGoperc.R–INS.R. ' In contrast, the 'repeat' proactive control requires minimal effort for suppressing interference from irrelevant task rules; instead, it can allocate more resources toward keeping internal goals and task-sets active in working memory, thus emphasizing the coupling within 'IFGoperc.L–INS.L.'

The Differential ACG-FC of 'IFGoperc.L–IFGtri.L,' which was not identified in AP I, specifically contributes to the 'repeat' condition. The ventrolateral PFC, including inferior frontal regions, preconfigures sensory and motor cortices through the sustained representation of conditional rules in anticipation of the upcoming target [10]. As 'repeat' proactive control demands fewer cognitive resources, the remaining resources can be directed toward anticipating target tasks and internalizing task rules [6]. This mechanism accounts for faster and more accurate reactions in target performance following 'repeat' cues.

### Comparison of ACG-FC and FC

FC metrics hypothetically treat all cortical regions as discrete entities, allowing each region to interact with any other, yielding $n(n-1)/2$ potential FCs for $n$ ROIs. However, within this vast candidate space, relying only on unsupervised phase synchrony or coherence to screen edges pair by pair often fails to map onto the structural basis that supports executive function, and it can also compress coordinated processes spanning regions and time into static pairwise associations. Empirically, coherence-based FCs, essentially evaluating coherence through cross-spectral density between signals [75], and phase-based FCs, evaluating instantaneous phase consistency extracted from Hilbert or wavelet transforms [109], are susceptible to volume conduction and common source effects. For example, within the common FCs during the 500–800 ms window post-cue, canonical metrics produced multiple similar functional interactions across anatomically adjacent cortical areas, such as the bilateral parallel areas (such as 'SFGdor.L–SFGdor.R', 'IFGoperc.L–IFGoperc.R', 'MFGc.R–SFGdor.L'), or multiple adjacent parietal regions connected to the same area (such as 'PCL.L–IFGoperc.L', 'PCL.L–PreCG.L', 'PCL.L–IFGtri.L').

In contrast, the ACG-FC framework incorporates structural connectivity as a prior pathway for information propagation within a RGNN, enabling end-to-end state discrimination learning directly from source signals. This design avoids information loss and task-irrelevant representations introduced by manual feature engineering. Moreover, the nonlinear modeling capacity of graph convolutional layers and recurrent units allows ACG-FC to characterize higher-order, nonlinear cross-regional interactions among multiple brain regions, rather than being constrained to the pairwise linear comparison framework. It complements FC metrics in revealing the neural mechanisms underlying inter-regional interactions. ACG-FCs complementarily identified the anatomical structures contributing most significantly to those potential functional interactions, specifically the prefrontal regulatory pathway ('SFGdor.L–SFGdor.R') and the motor-intention coupling pathway ('PCL.L–PreCG.L'); the former directs the collaborative function of the bilateral hemispheres toward goal-driven decision-making, while the latter directs interactions within parietal regions to align internal intentions with motor priming.

Notably, within the 500–800 ms window, both previous research [79] and the present study (Fig. 12 and Table 2) showed that FC analyses tend to highlight differential connections among sensorimotor regions—such as the fusiform gyrus (FFG), lingual gyrus (LING), and cuneus (CUN) responsible for visual processing, as well as the Heschl gyrus (HES) involved in primary auditory processing—more than the ACG-FC approach does. The activation levels of these visual and auditory regions inevitably increase after different cues, accompanied by differences in interregional connection strength. However, it is difficult to determine whether these unsupervised, signal-similarity-based FC differences originate from proactive control modes. In contrast, the ACG-FC approach generates FCs on the anatomical structure under the supervision of proactive control patterns, thereby effectively avoiding connectivity merely related to primary sensory processing.

Both the common and differential connectivity in FC methods radiated toward the right hemisphere, forming only a few connections (such as the common FCs in Coh, ImCoh, and PLV) or even no connections (such as the common FCs in PPC and the differential FCs in PLV and PPC). Furthermore, in the right hemisphere, ACG-FCs formed a self-organized Subnet 3 via the anatomically identified right INS, providing neural pathways underlying the FCs previously discovered by PLV analysis [79], such as ‘IFGtri.R–INS.R’ and ‘IFGoperc.R–INS.R’. Building on the cooperative interaction patterns identified by FC metrics during proactive task-switching, ACG-FCs emphasize the maintenance of activation in the current task set while emphasizing sustained inhibition of reactive interference from competing task sets. Moreover, ACG-FCs further localized the temporoparietal networks (Subnets 2 and 3 in Fig. 10) as anatomically symmetrical yet functionally complementary circuits responsible for proactive task-switching [79].

At the representational level of structure–function relationships, ACM-FC constrains conventional FC to the set of anatomically reachable connections, thereby providing a structurally grounded depiction of direct inter-regional functional interactions. In contrast, ACG-FC arises from a task-optimized learning process in which structural connectivity guides information propagation within the model and participates in feature integration and discrimination. Consequently, connection importance emphasizes the association between structural pathways and behavioral performance, rather than being determined solely by synchronization strength. The comparison between ACM-FC and ACG-FC provides an intuitive illustration of the procedural difference between using the anatomical template as a post-hoc mask and incorporating it as an internal prior within the model. Empirically, ACG-FC captures most of the direct functional coordination identified by the four FC metrics on anatomically reachable connections; it also includes additional edges that appear to supplement indirect interaction patterns that are difficult to express explicitly using FC alone.

In summary, ACG-FC quantifies the direct influence of signal variability in connective features on pattern recognition to uncover network-wise structure–function associations. Although the ACG-FC and FC networks exhibited overall convergent topological patterns, their specific connections did not correspond in a one-to-one manner. Therefore, the ACG-FC approach does not simply filter plausible connections from FCs using anatomical constraints; instead, it reflects a sparse and task-relevant organizational structure. ACG-FC extends FC—defined as direct functional interactions between regional pairs—to multi-regional functional interactions along cortical structural pathways. In the neural interaction mechanisms of proactive task-switching, ACG-FCs form anatomically dissociable yet functionally complementary frontoparietal and temporoparietal networks, thereby supplementing conventional FC by revealing the anatomical cortical pathways that mediate functional interactions.

### Empirical validation of the ACG-FC approach

The empirical evidence supports the effectiveness of the ACG-FC approach in uncovering the neural mechanisms of proactive task-switching. First, the T-GCN model achieved stable accuracy over 80% in discriminating EEG signal epochs of cue trials, ensuring comprehension of the importance of signal features within neural circuits. Second, ACG-FCs and FCs exhibited convergent validity at the regional level, identifying similar areas such as the frontoparietal and temporoparietal cortices, but diverged at the connection level. ACG-FCs formed sparser networks (15 vs. 19 links at 500–800 ms for PLV; 6 vs. 10 links at 900–1100 ms for ImCoh), suggesting they capture more refined subsets of anatomical pathways underlying the broader functional interactions reflected by FC. Moreover, non-parametric tests with random networks provided evidence for the significance of topological similarity between ACG-FC and FC networks, as their SPDs were generally more than 30% lower than the mean SPD of the null distribution. The differences between ACG-FC and FC approaches were primarily the ability of ACG-FC to clearly map functional relevance onto the frontoparietal “multiple-demand” and cingulo-opercular anatomical structures.

Both common and Differential ACG-FCs also demonstrated stronger associations with behavioral performance. Although linear correlation cannot fully capture the relationship between (ACG-)FC values and behavioral responses, it nonetheless provides evidence supporting the effectiveness of ACG-FC in probing proactive control mechanisms. Common ACG-FCs were fewer in number than the corresponding FCs and showed stronger correlations with the first-target RTs across same-order connections. Under the higher-connectivity condition, Differential ACG-FCs, via stepwise regression, accounted for over 25.2% of the variance in behavioral RT with fewer edge variables. Notably, in the 500–800 ms window, regression models on ‘switch’ trials yielded higher *R*^*2*^ for the first-target RT than those on ‘repeat’ trials, whereas in the 900–1100 ms window the reverse pattern emerged with even larger *R*^*2*^. This may reflect the predominance of proactive task-switching control during the earlier latency window, whereas temporal expectation of targets dominates in the later window, with the first-target RTs—serving as the behavioral criterion—being more strongly influenced by target anticipation.

### Limitations and future directions

The ACG-FC extends existing FC from signal-dependent analysis between pairwise regions to signal-anatomy-dependent network analysis involving multiple regions and structural connections, providing an anatomical extension on studying the neural interaction mechanisms. Although the standard 32‑channel EEG montage already supplies low‑spatial‑resolution cortical dynamics for low‑frequency ACG-FC analyses, future works using 64–128‑channel high‑density EEG sampling could identify connections with greater spatial precision and in deeper anatomical regions.

Moreover, this study adopted a group-level deterministic connectome template as the structural prior primarily for reasons of data availability and interpretability. In the absence of individual diffusion MRI data, this template provides the RGNN with a stable and reproducible macroscale anatomical structure to validate the practical feasibility of a basic analytical framework—namely, learning proactive control patterns from EEG signals organized by structural topology and localizing connective features that contribute significantly to pattern recognition via attribution analysis. Admittedly, a group-level binarized connectome template is a compromise approximation of individual-level connectomes and may reduce sensitivity to weaker connections and inter-individual variability. Future work may benefit from extending this framework to individual-level probabilistic structural connectivity, for example by incorporating a connectivity probability matrix [57] such that the RGNN’s MPF is modulated by edge-wise propagation probabilities. This would generalize the input to “individual EEG + individual probabilistic connectome,” which is expected to further improve model performance and enhance the granularity and validity of ACG-FC.

In the present study, ACG-FC was implemented within a task-supervised setting, in which the functional relevance of each structural pathway was quantified according to its contribution to discriminating experimentally defined proactive control states. Therefore, in its current form, it is not directly applicable to task-free paradigms. However, the graph-learning framework itself is not inherently limited to task-evoked data. If alternative supervisory objectives are introduced, such as self-supervised representation learning, data-driven state clustering, or externally defined phenotypic or group labels, the same structure-guided learning logic may be extendable to task-free paradigms. Future work should therefore examine whether graph-based anatomical priors can support the discovery of anatomically guided functional interactions in resting-state or other task-free neurophysiological data.

Beyond technical and methodological considerations, it is essential to clarify the conceptual role of the ACG-FC approach. ACG-FC is not a direct measurement of the functional effects of individual white-matter tracts; rather, we treat a group-level structural connectivity template as a reproducible spatial prior to compensate for the lack of permutation invariance when source-level EEG is represented as tensors. On this basis, a task-optimized RGNN is used to retrospectively evaluate the information contribution of each edge in the structural prior to functional pattern recognition. Consequently, under the guidance of a macroscale structural prior with relatively reliable connectivity, ACG-FC localizes anatomical pathways that make significant statistical marginal contributions to task-related EEG patterns, providing a structured and interpretable complement to FC, which captures functional interactions mediated through both direct and indirect polysynaptic pathways. This focus enhances anatomical specificity and interpretability. Accordingly, ACG-FC and FC offer distinct yet complementary insights: FC delineates broad patterns of functional coordination across networks, whereas ACG-FC pinpoints the anatomical pathways that contribute significantly to these task-related patterns of functional interaction. Nonetheless, attribution scores quantify the statistical contribution to pattern recognition rather than direct physiological causation. The moderate behavioral correlations (*R*^*2*^ = 0.250–0.350) suggest that connection patterns—whether anatomical (on the cortical surface) or functional—explain only part of the variance in task-switching performance, with substantial variance attributable to other factors beyond connectivity measures. Leunissen et al. [74] demonstrated that decreased task-switching capabilities correlated with lower integrity of subcortical white-matter pathways between the PFC and striatum.

## Conclusion

In this study, we examined functional connectivity potentially involved in proactive control during task-switching by analyzing low-frequency EEG signals recorded in the preparation phase of a task-switching paradigm, under the guidance of cortical anatomical connectivity priors. For the first time, we integrated DTI-derived cortical connections as spatial structures into RGNNs to investigate neural dynamic interactions associated with proactive task-switching. The results indicate that introducing a deterministic anatomical connectivity template as a spatial structural prior and organizing cortical source activity into a graph-structured temporal representation enhance recognition performance in proactive control mode, supporting the view that spatial prior information facilitates DNN-based identification of neural activity patterns. Furthermore, through attribution analysis of the task-optimized model, the marginal contributions of macroscopic structural edges to classification decisions are quantified as ACG-FC values, enabling the identification of pathways (ACG-FCs) that are most critical for discriminating functional patterns. This framework extends functional relationships from pairwise brain regions to functional subnetworks composed of multiple structural pathways, offering an interpretable, structure-informed complement for understanding the functional relevance of proactive task-switching and forming a synergistic perspective with conventional FC.

## Supplementary Information

Supplementary Material 1.

## Data Availability

The datasets generated and analyzed in this study, together with the optimal models and experimental analysis code, are available in the Open Science Framework repository (https://osf.io/p2vka/). Owing to commercial copyright restrictions imposed by Panasonic Holdings Corporation, the raw EEG recordings (in “.bdf” format) from the full experiment are not publicly accessible. However, we instead release pre-processed EEG data (in “.fif” format) from the task-switching cue trials, which supported all modeling and analytical procedures in our research, along with the corresponding data preprocessing scripts. In addition, the down-sampled signal sequences from de-identified cue epochs—employed in the modeling and analysis notebooks—are provided to enable readers to verify the implementation rapidly. A detailed description of the supplementary materials is likewise available at https://osf.io/p2vka/.

## Abbreviations

AAL atlas: Anatomical automatic labeling atlas
ACCc: Anterior cingulate cortex, caudal part
ACG-FC: Anatomical-connectivity-guided functional connectivity
ACG-FCs: Anatomical-connectivity-guided functional connections
ACM-FC: Anatomical-connectivity-masked functional connectivity
ACM-FCs: Anatomical-connectivity-masked functional connections
ANOVA: Analysis of variance
AP I / II: Attribution process I/II
AUROC: Area under the receiver operating characteristic curve
BEM: Boundary element method
CI: Confidence interval
CNN: Convolutional neural network
CNV: Contingent negative variation
Coh: Coherence
CRNN: Convolutional recurrent neural network
CTI: Cue-to-target interval
DK atlas: Desikan–Killiany atlas
DNN: Deep neural network
dSPM: Dynamic statistical parametric mapping
DTI: Diffusion tensor imaging
DyGGNN: Dynamic gated graph neural network
ECG: Electrocardiography
ECN: Execution control network
EEG: Electroencephalography
ER: Error rate
ERP: Event-related potential
EvolveGCN: Evolving graph convolutional network
F1: F1 score (harmonic mean of precision and recall)
FC: Functional connectivity
FCs: Functional connections
FD: Frobenius distance
FDR: False discovery rate
fMRI: Functional magnetic resonance imaging
GCN: Graph convolutional network
GFP: Global field power
GG correction: Greenhouse–Geisser correction
GNN: Graph neural network
GRU: Gated recurrent unit
HES: Heschl gyrus
ICA: Independent component analysis
IFGoperc: Inferior frontal gyrus, opercular part
IFGorb: Inferior frontal gyrus, orbital part
IFGtri: Inferior frontal gyrus, triangular part
IFJ: Inferior frontal junction
IG: Integrated gradient
ImCoh: Imaginary coherence
INS: Insula
ITG: Inferior temporal gyrus
JS: Jaccard similarity
LightGBM: Light gradient boosting machine
LIME: Local interpretable model-agnostic explanation
LOSS: Cross-entropy loss
LPC: Late positive component
LRP: Layer-wise relevance propagation
LSTM: Long short-term memory
MCC: Matthew’s correlation coefficient
MEG: Magnetoencephalography
MFGc: Middle frontal gyrus, caudal part
MFGr: Middle frontal gyrus, rostral part
ML: Machine learning
MPF: Message passing function
MTGv: Middle temporal gyrus, ventral part
ORBsup: Superior frontal gyrus, orbital part
P3: P300 component
PC: Parietal cortex
PCL: Paracentral lobule
PCUN: Precuneus
PFC: Prefrontal cortex
PHG: Parahippocampal gyrus
PLV: Phase locking value
PoCG: Postcentral gyrus
PPC: Pairwise phase consistency
PPI: Psychophysiological interaction
PreCG: Precentral gyrus
RGNN: Recurrent graph neural network
RNN: Recurrent neural network
ROI: Region of interest
RT: Reaction time
SD: Standard deviation
SE: Standard error
SFGdor: Superior frontal gyrus, dorsolateral part
SFGmed: Superior frontal gyrus, medial part
SMG: Supramarginal gyrus
SOA: Stimulus onset asynchrony
SPD: Spectral distance
SpecCSP: Spectrally weighted common spatial patterns
S–R: Stimulus–response
SSP: Spatial signal projector
STG: Superior temporal gyrus
SVC: Support vector classifier
T-GCN: Temporal graph convolutional network
TPA-LSTM: Temporal pattern attention-based LSTM
TPO: Temporal pole
wMNE: Weighted minimum norm estimation
