# Supplementary material for: Anatomical-connectivity-guided functional connectivity reveals task-relevant pathways during proactive task-switching via recurrent graph neural networks
Source: Brain Inform. 2026 Apr 26;13(1):20. doi: 10.1186/s40708-026-00300-6 (PMC13237348; doi:10.1186/s40708-026-00300-6)
Supplement: Supplementary file 1 — Supplementary Material 1. [file 40708_2026_300_MOESM1_ESM.docx]

***Model Performance Evaluation***

The classification performance was evaluated by metrics including accuracy (values range [0, 1]), F1 score (F1, values range [0, 1]), and Matthews correlation coefficient (MCC, values range [-1, 1]). Our samples are balanced in two categories (“repeat” vs. “switch”). Given the confusion matrix of the classification result, these metrics are defined as below:

$$ACC= \frac{TP+TN}{TP+TN+FP+FN}$$

$$F1=\frac{2TP}{TP+FN+TP+FP}$$

$$MCC=\frac{TP*TN-FP*FN}{\sqrt{(TP+FP)(FN+TP)(FN+TN)(FP+TN)}}$$

whereTP refers to True Positives, which are the correctly identified positive samples; FP is False Positives, representing incorrectly identified positive samples; FN stands for False Negatives, meaning the incorrectly identified negative samples; and TN represents True Negatives, correctly identified negative samples.

In addition to metrics noted above, based on the category probability distribution output by the binary classifiers, we used cross-entropy loss (LOSS) and area under the ROC curve (AUROC, values range [0.5, 1]) in test set to evaluate the model performance of identifying cue stimuli. The ROC curve is a plot of the true positive rate (TPR) versus the false positive rate (FPR) at various threshold settings. The area under the ROC curve, known as AUROC, can be regarded as the model's overall ability to correctly identify positive instances and avoid false alarms, regardless of the threshold.

$$LOSS= \frac{-\sum_{i} (y_{i}\log\left( p_{i} \right)+(1-y_{i})log(1-p_{i}))}{N}$$

where $y_{i}$ and $p_{i}$ represent the real cue stimulus and identified probability in the positive class for the $i\mathrm{th}$ sample respectively. $N$ is the number of samples.

Specifically, the ACC is used to assess the recognition accuracy: the higher the value, the better the recognition performance. The LOSS evaluates recognition errors: the lower the value, the better the model performance. F1 and MCC measure the consistency between identified labels and ground truth: the larger the value, the better the identification effect. The AUROC gauges the overall discriminative ability across two categories: the higher the value, the better the classification performance.

***“Integrated Gradients” Attribution Algorithm***

Formally, suppose we have a deep network $H:X\to\left[ 0, 1 \right]$ that outputs the probability $p=H\left( x \right)$for an input $x\in X.$After thresholding, the binary prediction is $\hat{y}\left( x \right)=argmax\left\{ 1-H\left( x \right), H\left( x \right) \right\}\in\left\{ 0, 1 \right\}$. Within the Integrated Gradients (IG) framework, let $x\in X$ be the current input and $x^{'}\in X$ the baseline input. For image networks, $x^{'}$ could be a black image; For text sequence networks, $x^{'}$ could be the zero embedding vector; For graph neural networks, the input consists of node features $x\in R^{m}$ and a graph topology $G\in R^{n\times n}$. In this case, the baseline graph $G^{'}\in R^{n\times n}$ is an adjacency matrix in which the connective features of interest have been set to zero. The object on which we calculate integrated gradients can be regarded as the message passing function (MPF) inside RGNNs. The pattern probability generated by the deep graph neural network is hereby defined as $p:=H\circ MPF:R^{n\times n}\times R^{m}\to\left[ 0, 1 \right], (G,x\boldsymbol{)⟼}p(G,x\boldsymbol{)}$.

We consider the straight-line path from the baseline $MPF(G^{'};x)$ to the input $MPF(G;x)$ and accumulate the gradients along this path. Specifically, the integrated gradient of the 𝑖-th dimension with respect to the input graph is $G$ defined as

$\text{IntegratedGrads}_{i}(G)::=\Delta{MPF}_{i}\times\int_{0}^{1} \frac{\partial H(MPF(G^{'};x)+\alpha\times\Delta MPF)}{\partial MPF\left( G_{i};x \right)}d\alpha$ (1)

Where $\Delta MPF=MPF(G;x)-MPF\left( G^{'};x \right),\Delta MPF_{i}=MPF\left( G_{i};x \right)-MPF\left( G_{i}^{'};x \right).$

***RGNN Variants for Discerning Task-Switching Preparation***

***Temporal Graph Convolutional Network***

The T-GCN consists of two pivotal components: the GCN and the GRU. The GCN is utilized to capture the spatial features of the topological structure in the cortical network, incorporating multiple stacked layers of spectral convolution to perform the neighborhood aggregation (Hamilton et al., 2018). At the time point *t*, the $l$-th convolution receives the adjacency matrix $\boldsymbol{A}$ and the node feature matrix $\boldsymbol{H}_{t}^{(l)}$ as inputs. The GCN grasps spatial relationships among the nodes by their first-order neighborhood and updates the feature matrix to $\boldsymbol{H}_{t}^{(l+1)}$ as the output using a weight matrix $\boldsymbol{W}_{t}^{(l)}$. Mathematically, the process of temporal graph convolution can be expressed as:

$\boldsymbol{H}_{t}^{(l+1)}=GCONV(\boldsymbol{A},\boldsymbol{H}_{t}^{\left( l \right)}, \boldsymbol{W}_{t}^{(l)})=\boldsymbol{\sigma}(\hat{\boldsymbol{A}}\boldsymbol{H}_{t}^{\left( l \right)}\boldsymbol{W}_{t}^{(l)})$(2)

where $\hat{A}$is the normalization of $\tilde{A}$ defined as $\hat{A}=\tilde{D}^{-\frac{1}{2}}\tilde{A}\tilde{D}^{-\frac{1}{2}}$, $\tilde{A}=A+I_{N}$ is the adjacency matrix with added self-connections ($I_{N}$ is the identity matrix), $\tilde{D}=\sum_{j} \tilde{A}_{ij}$ is the degree matrix. The initial embedding matrix comes from the inversely traced signals, represented as $H_{t}^{(0)}=X_{t}$. $\sigma$ represents an activation function, such as ReLU or Sigmoid, which are commonly used in the activation layers of neural networks. The state from the final hidden layer $L$, $H_{t}^{(L)}\in R^{N\times M}$, where $M$ is the number of hidden units in this last hidden layer, denotes the ultimate high-order representations of nodes from initial signals.

The operation process for a T-GCN Unit is expressed as the Equation 3. $GCONV(A,X_{t}, W_{t})$ herein represents the graph convolution process, essentially indicating the point-wise multiplication. $z_{t}$ and $r_{t}$ are the update and reset gates, respectively, while $h_{t}$ represents the output at time $t$. The symbol $b$ stands for the biases in the training process, and $\odot$ denotes the Hadamard product.

$${\boldsymbol{function} h}_{t}= TGCU(A, X_{t},W_{t})$$

$$H_{t}^{(1)}=GCONV(A,X_{t}, W_{t})$$

$$z_{t}=\sigma\left( W_{z}\left[ H_{t}^{(1)},h_{t-1} \right]+b_{z} \right)$$

$$r_{t}=\sigma(W_{r}[H_{t}^{(1)},h_{t-1}]+b_{r})$$

$$c_{t}=tanh(W_{c}[H_{t}^{(1)},(r_{t}\odot h_{t-1})]+b_{c})$$

$$h_{t}=z_{t}\odot h_{t-1}+(1-z_{t})\odot c_{t}$$

$\boldsymbol{end function}$(3)

***Evolving Graph Convolutional Network***

The EvolveGCN, another RGNN model capable of processing graph sequence, implements a learnable graph convolution mechanism through its architecture (Pareja et al., 2020). This mechanism continuously updates the weight matrix $W_{t}^{(l)}$ in GCN based on current and historical information to reflect the variations in the attributes of graph nodes over time. Depending on how the weight matrix evolves, the EvolveGCN unit is developed into two distinct versions: EvolveGCU-H and EvolveGCU-O. The weight $W_{t}^{(l)}$ is regarded as the hidden state in the -H version, while as inputs/outputs in the -O version:

$$\boldsymbol{function}[H_{t}^{\left( 1 \right)},W_{t}]=\mathrm{EGCU}-O(A,X_{t},X_{t-1},W_{t-1})$$

$$W_{t}=\mathrm{LSTM}( X_{t-1},W_{t-1})$$

$$H_{t}^{(1)}=\mathrm{GCONV}(A_{t},X_{t},W_{t})$$

$\boldsymbol{end function}$(4)

$$\boldsymbol{function}[H_{t}^{(1)},W_{t}]=\mathrm{EGCU}-H(A,X_{t},W_{t-1})$$

$$W_{t}=\mathrm{GRU}(X_{t},W_{t-1})$$

$$H_{t}^{(1)}=\mathrm{GCONV}(A_{t},X_{t},W_{t})$$

$\boldsymbol{end function}$(5)

The implementation of the -H version requires two extensions on the standard GRU:

1. Expanding the input and hidden state of the GRU from vectors to matrices, as illustrated by the formula below. The parameter matrices of the expanded GRU, $Z_{t}$, $R_{t}$ and $C_{t}$, correspond to the update gate, reset gate, and candidate hidden state at time $t$, respectively.

$$\boldsymbol{function} W_{t}=g(X_{t}^{*},W_{t-1})$$

$$Z_{t}=\sigma(W_{Z}X_{t}^{*}+U_{Z}W_{t-1}+B_{Z})$$

$$R_{t}=\sigma(W_{R}X_{t}^{*}+U_{R}W_{t-1}+B_{R})$$

$$C_{t}=\tanh(W_{C}X_{t}^{*}+U_{C}(R_{t}\odot W_{t-1})+B_{C})$$

$$W_{t}=(1-Z_{t})\odot W_{t-1}+Z_{t}\odot C_{t}$$

$\boldsymbol{end function}$(6)

1. The second extension is that the number of columns in the feature input of the GRU must match that of the hidden state. To achieve this, a summarization strategy is adopted to introduce a parameter vector $p$ independent of the time index $t$ to calculate the weights for all rows in the matrix $X_{t}$ and singles out the top $k$ weighted rows as the output $X_{t}^{*}$, as formalized in the following pseudocode.

$$\boldsymbol{function} X_{t}^{*}=summarize(X_{t},k)$$

$$y_{t}=X_{t}p/\parallel p\parallel$$

$$i_{t}=\text{top-indices}(y_{t},k)$$

$$X_{t}^{*}=[X_{t}\odot\tanh(y_{t})]_{i_{t}}$$

$\boldsymbol{end function}$(7)

Given the above functions $g$ and $summarize$, the complete recurrent architecture of the -H version can now be specified:

$$W_{t}=\mathrm{GRU}(X_{t},W_{t-1})$$

$:=g(summarize(X_{t},\#col(W_{t-1}))^{T},W_{t-1})$ (8)

where $\#col$ indicates the number of columns in the matrix, and the superscript $T$ denotes matrix transpose. It summarizes the embedding matrix $X_{t}$ into a matrix with appropriate dimensions and then evolves the weight matrix from the last state $W_{t-1}$ into the current state $W_{t}$.

Implementing the -O version simply involves a straightforward extension of the standard LSTM from its vector-based format to a matrix-based one. The parameter matrices of the extended LSTM, $F_{t}$, $I_{t}$, $C_{t}$ and $O_{t}$, correspond to the forget gate, input gate, candidate memory, and output gate at time $t$, respectively. The following is the pseudocode for a complete recurrent architecture:

$$\boldsymbol{function} W_{t}=\text{LSTM}(W_{t-1}, X_{t-1})$$

$$F_{t}=\sigma(W_{F}W_{t-1}+U_{F}X_{t-1}+B_{F})$$

$$I_{t}=\sigma\left( W_{I}W_{t-1}+U_{I}X_{t-1}+B_{I} \right)$$

$$O_{t}=\sigma(W_{O}W_{t-1}+U_{O}X_{t-1}+B_{O})$$

$$\tilde{C}_{t}=\tanh(W_{C}W_{t-1}+U_{C}X_{t-1}+B_{C})$$

$$C_{t}=F_{t}\odot C_{t-1}+I_{t}\odot\tilde{C}_{t}$$

$$W_{t}=O_{t}\odot\tanh(C_{t})$$

$\boldsymbol{end function}$(9)

***Dynamic Gated Graph Neural Network***

The Gated Graph Neural Network (GGNN; Taheri & Berger-Wolf, 2019) utilizes message passing in the spatial domain to signify node embeddings of graph-structured data. Similar to GCN, GGNN propagates messages from a node to all its neighboring nodes to update the hidden states of all reachable nodes. At the time point $t$, its message passing also involves incorporating information from previous propagation iterations into the current hidden state $H_{t}^{\left( l \right)}$ to update it to the next $H_{t}^{\left( l+1 \right)}$, calculated as follows:

$$\boldsymbol{function} H_{t}^{(l+1)}=\mathrm{GGNN}(A,H_{t}^{\left( l \right)})$$

$$\begin{matrix} Z_{t} & = & \sigma(W_{z}(AH_{t}^{\left( l \right)}+B)+U_{z}H_{t}^{\left( l \right)}) \end{matrix}$$

$$R_{t}=\sigma\left( W_{R}(AH_{t}^{\left( l \right)}+B)+U_{R}H_{t}^{\left( l \right)} \right)$$

$$C_{t}=\tanh(W(AH_{t}^{\left( l \right)}+B)+U_{C}(R_{t}\odot H_{t}^{\left( l \right)}))$$

$$H_{t}^{(l+1)}=(1-Z_{t})\odot H_{t}^{\left( l \right)}+Z_{t}\odot C_{t}$$

$\boldsymbol{end function}$(10)

Similarly, the input and hidden state of the GRU are also expanded to matrices, as well as the introduced bias matrix $B$. The $W$ and $U$ are parameter matrices. The symbol $\sigma$ denotes the sigmoid activation function to ensure outputs ranging within (0, 1) to obtain the probability distribution. Furthermore, the introduction of the hyperbolic function $tanh$ adds essential non-linearity, improving the model expression ability. The average pooling of a series of node representations $H_{t}^{\left( l \right)}$ at each time point $t$, is fed into the LSTM unit to form the DyGGNN model capable of capturing long-term dependencies of the graph sequences, as illustrated in the formula below.

$h_{t}=LSTM(Avg(H_{t}^{\left( l \right)}), h_{t-1})$ (11)

With the current graph embedding $Avg(H_{t}^{(l)})$ and its last historical state $h_{t-1}$, DyGGNN employs the LSTM to project the dynamic embedding $G_{t-1}$ pertaining to past graph information into the current hidden representation within the designated observation window.

***Network Structure Similarity***

The similarity between network structures was evaluated using three metrics: Jaccard similarity, spectral distance, and Frobenius distance. These metrics assess the commonalities and differences between network structures from distinct perspectives. The definitions and purposes of each metric are detailed as follows:

**Jaccard Similarity**. Jaccard similarity measures the proportion of common edges to the union of edges between two functional connectivity networks. This metric captures the degree of overlap in network connections and is defined as:

$$J(A,B)=\frac{\left| E_{A}\cap E_{B} \right|}{\left| E_{A}\cup E_{B} \right|}$$

where $E_{A}\mathrm{and}E_{B}$ represent the edge sets of networks $A$ and $B$, respectively. The value of $J (A,B)$ ranges between 0 and 1, with higher values indicating greater similarity in edge composition.

**Spectral Distance.** Spectral distance focuses on comparing the spectral properties of networks, such as eigenvalues or the Laplacian spectrum. It evaluates the similarity of networks in the frequency domain, reflecting their dynamic characteristics. This metric is defined as:

$$S\left( A,B \right)= \left\| \lambda_{A}-\lambda_{B} \right\|_{2}$$

where $\lambda_{A}$ and $\lambda_{B}$ denote the eigenvalue vectors of networks $A$ and $B$ respectively. This metric is particularly useful for identifying associations in network topology dynamics.

**Frobenius Distance.** Frobenius distance quantifies the overall structural differences between two networks by comparing their adjacency matrices. It is defined as:

$$D_{F}\left( A,B \right)=\parallel A-B\parallel_{F}$$

where$A$ and $B$ are the adjacency matrices of networks A and B, and $\parallel\cdot\parallel_{F}$ denotes the Frobenius norm. A smaller Frobenius distance indicates higher similarity in the global structure of the networks.

Specifically, Jaccard similarity captures the similarity in edge composition, where a higher value indicates more shared connections. The spectral similarity emphasizes the alignment of spectral properties, reflecting the consistency in dynamic topological features. Frobenius distance quantifies the differences in the overall structure by measuring discrepancies in the adjacency matrices, with smaller values indicating closer structural resemblance.

**Supplementary Figure 1**

*Average Cortical* *Anatomical Network Constructed via DTI Tractography*


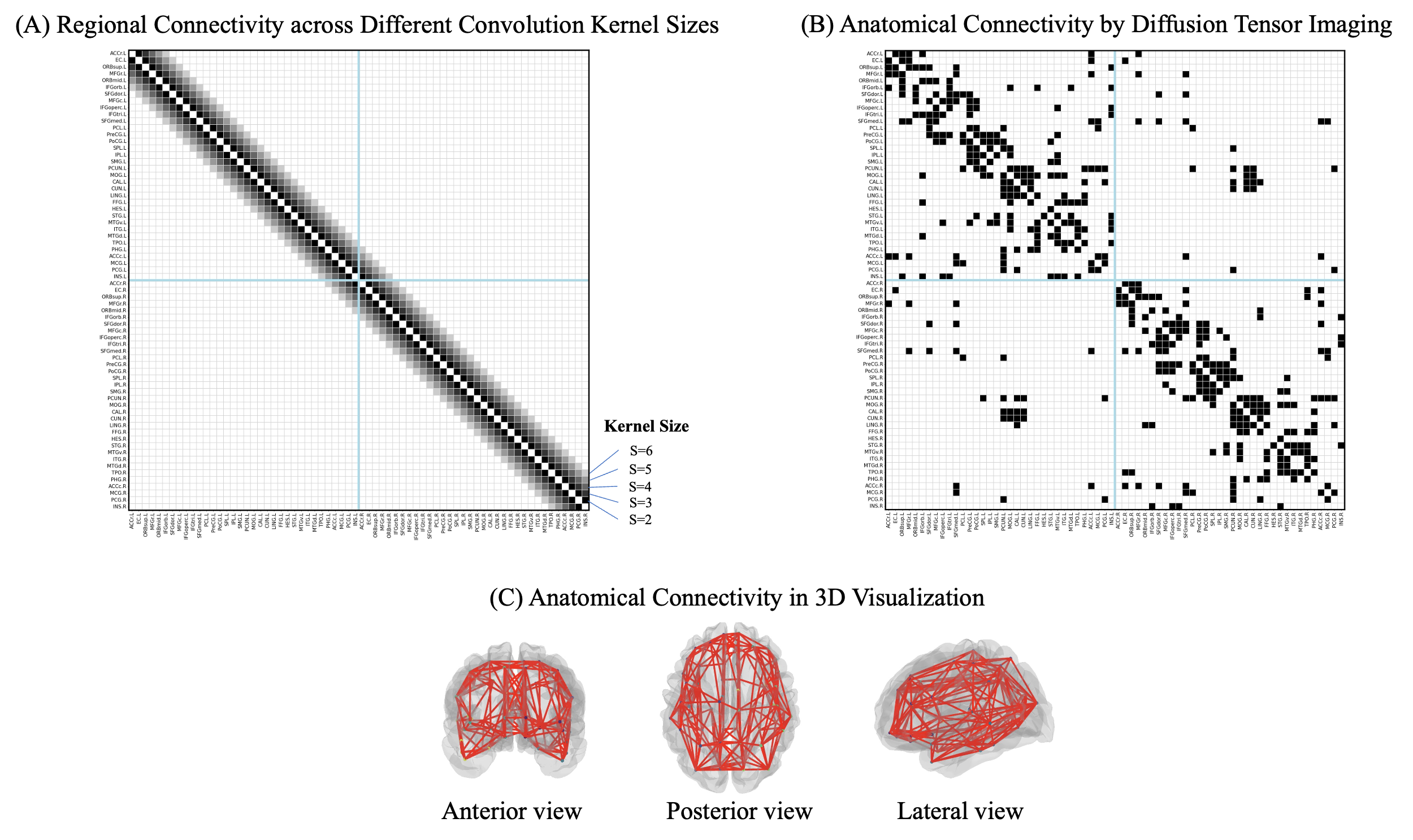


*Note*. (A) Binary heatmap of regional connections with various sizes of convolution kernels. Both the horizontal and vertical axes represent the total number of brain regions in the DK atlas, with hemisphere abbreviations (R: right; L: left). When processing multi-dimensional time series using 1D-CNNs, the spatial scale of the convolutional kernel determines regional adjacency connectivity. In the adjacency matrix, region pairs with connections are represented in black. (B) Binary heatmap of structural connections among brain regions. In the adjacency matrix of the average cortical network, black entries indicate regional pairs with connections. (C) 3D visualization of structural connectivity obtained through DTI tractography. This visualization showcases the connectivity network from multiple perspectives (anterior, superior, lateral). The volume of each ROI is concentrated at its centroid, with red-colored nerve fiber connections.

**Supplementary Figure 2**

*Trial Number of Successful Responses to Cues across Participants*


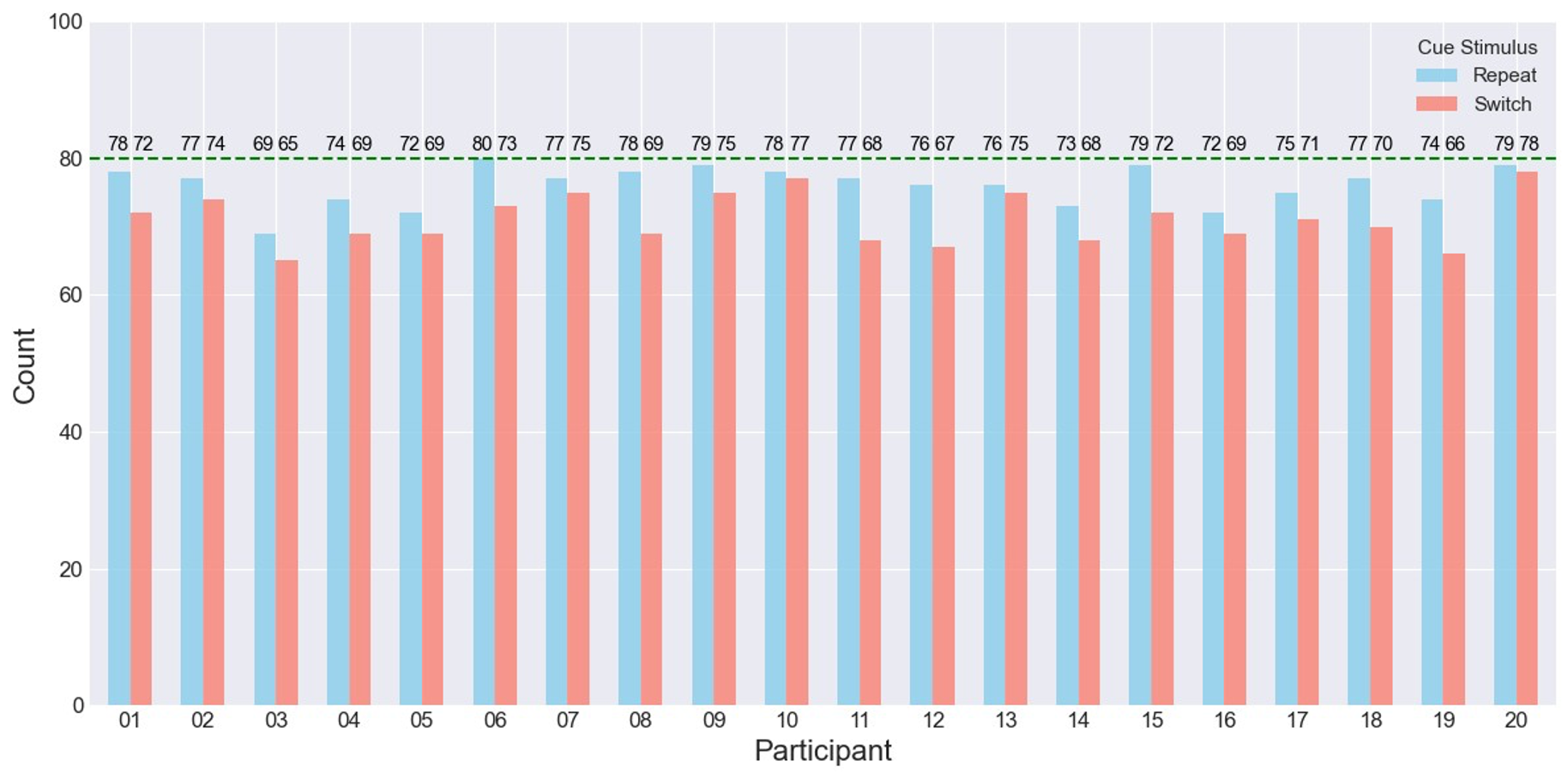


*Note.* The trial number of effectively updating the S-R mapping is presented at participant level, with blue and red bars representing the repeat and switch scenarios, respectively. The green horizontal dashed line signifies the total number of either 'repeat' or 'switch' cues, set at 80.

**Supplementary Figure 3**

*Connectivity Differences of Differential FCs Between Switch and Repeat Trials Across Distinct Metrics*


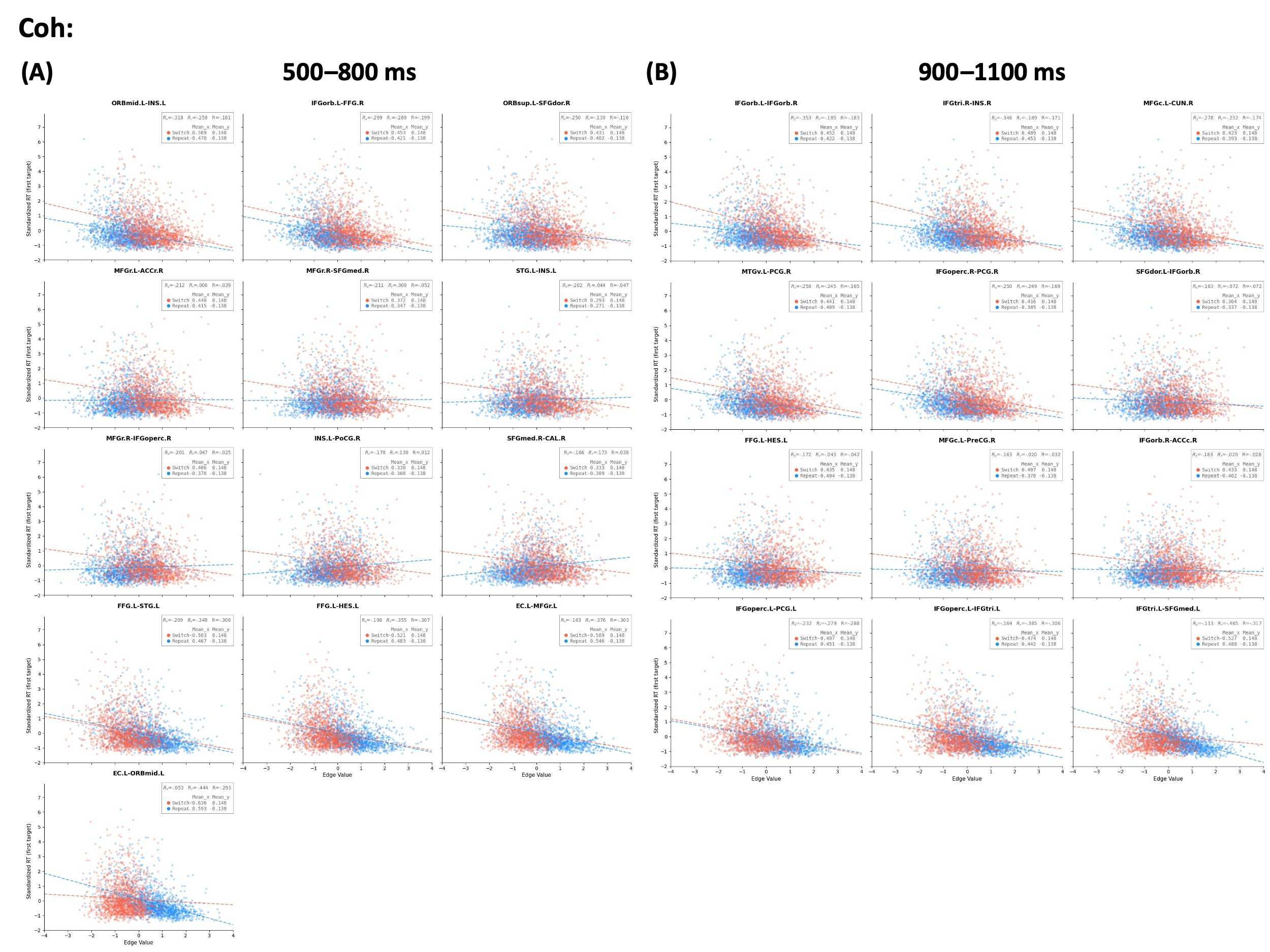


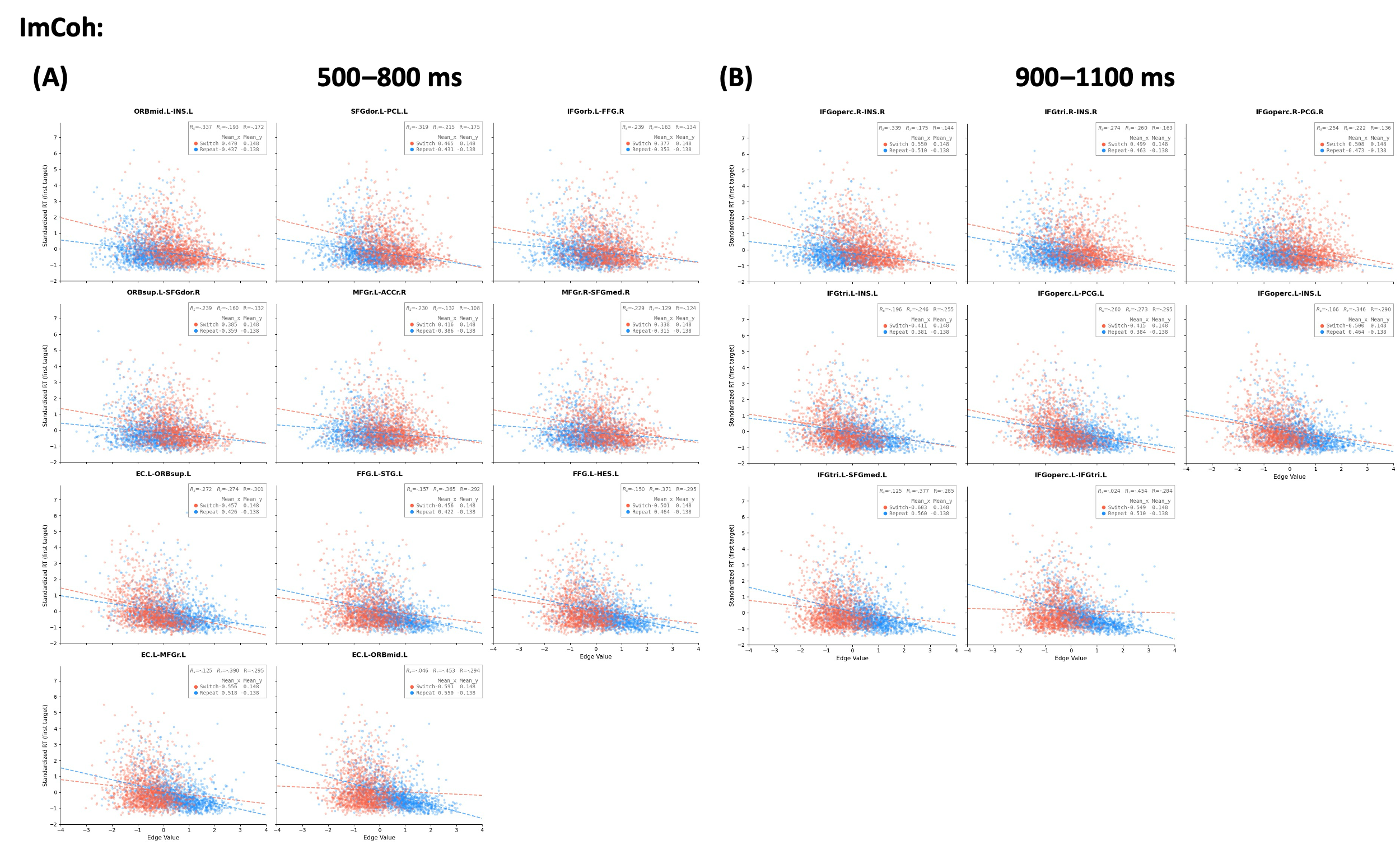


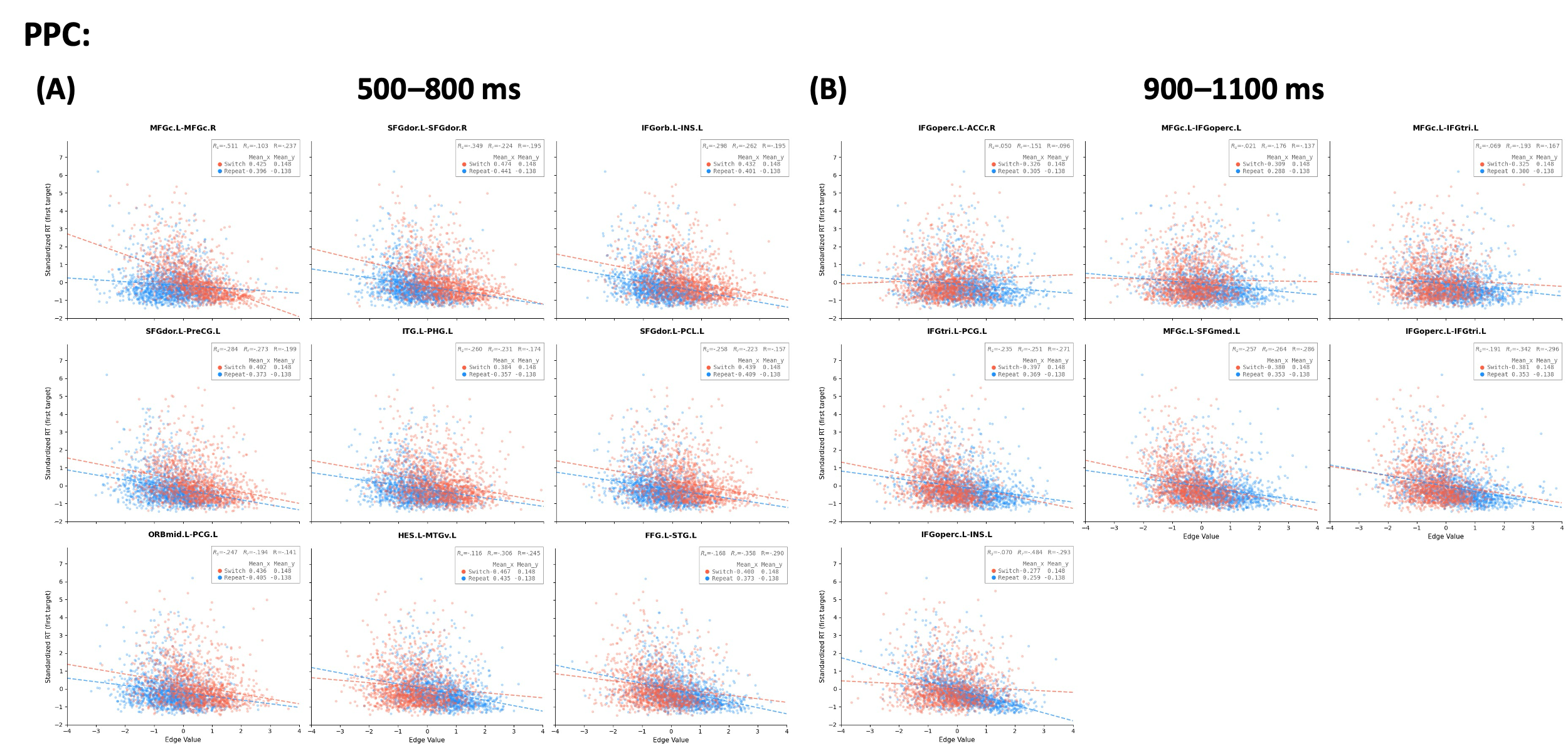


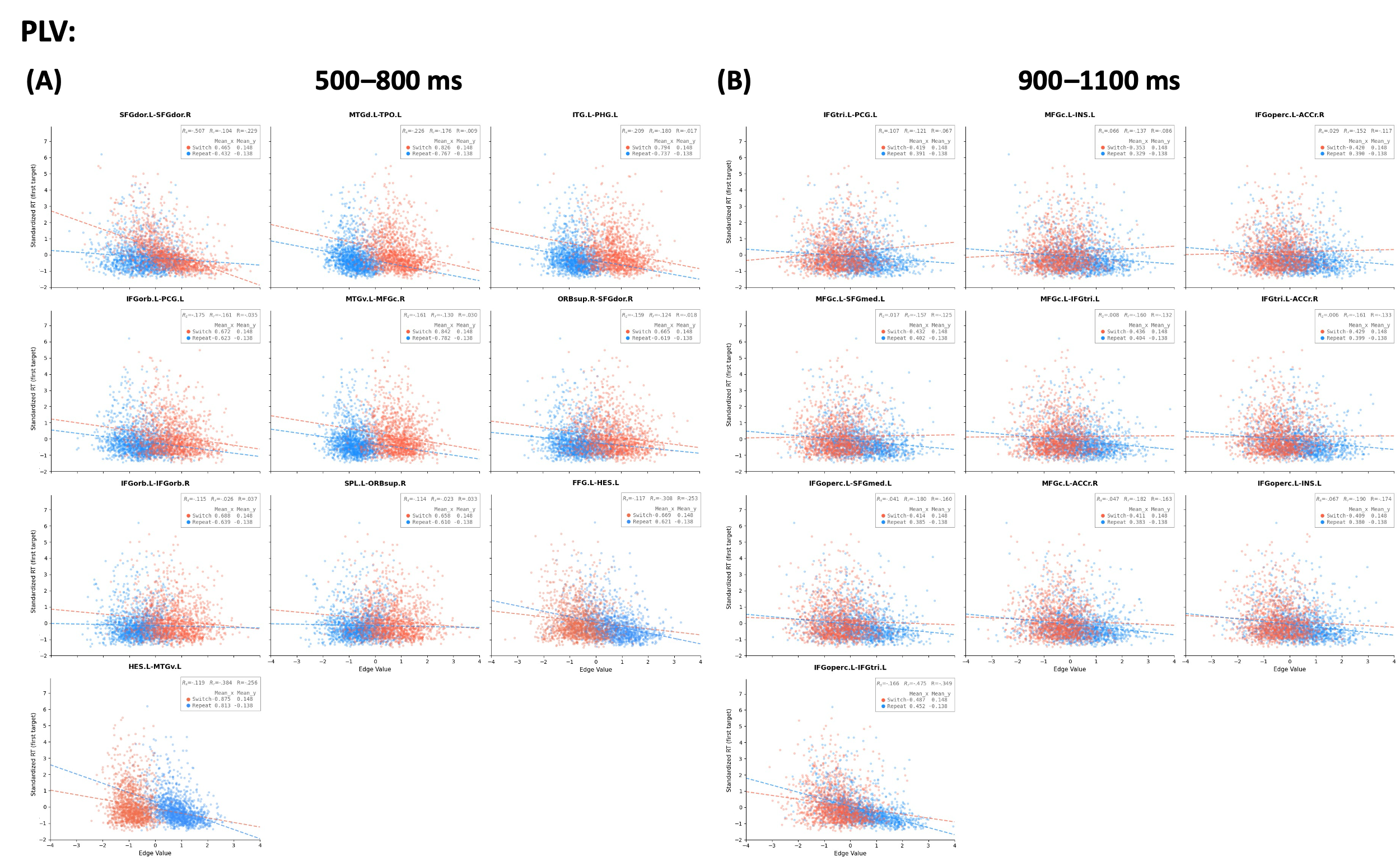


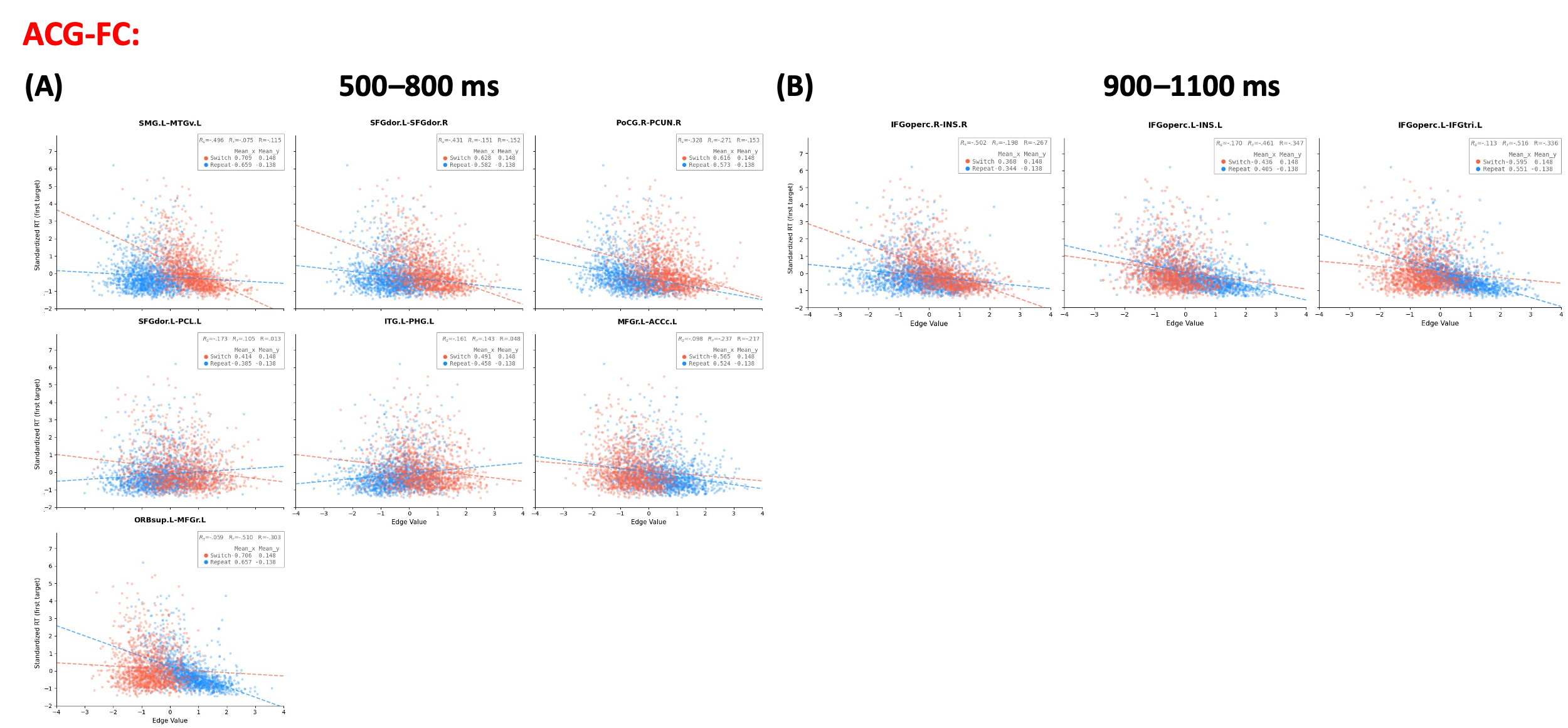


*Note*: For each connectivity metric, significantly differential FCs observed at 500–800 ms (A) and 900–1100 ms (B) after switch (blue dots) and repeat (red dots) cues are plotted against the reaction time (RT) of the first target across trials. The RTs of the first target were standardized at the trial level and transformed into z-scores to represent behavioral performance on the y-axis. Likewise, each functional connectivity value was standardized and converted into z-scores to illustrate its correlation with behavioral performance. The correlation coefficients for switch and repeat trials, as well as overall, are displayed in the upper right corner of each subplot, along with the means of connectivity values and RTs. In the subplots, blue and red lines depict the fitted relationships between connectivity and behavioral performance for trials in the repeat and switch conditions, respectively.

**Supplementary Table 1**

*Reaction Times, Error Rates, and Cue-Trial Counts for Each Subject in Repeat vs. Switch Conditions*

|  | **Reaction Time (ms) ± SE** | | | | | | | | |  | **Error Rate (%) ± SE** | | | | | | | | | | |  | | **Valid Cue Trials** | | | |
| --- | --- | --- | --- | --- | --- | --- | --- | --- | --- | --- | --- | --- | --- | --- | --- | --- | --- | --- | --- | --- | --- | --- | --- | --- | --- | --- | --- |
|  | **Repeat** | | | |  | **Switch** | | | |  | **Repeat** | | | | |  | **Switch** | | | | | |  | | **Repeat** | **Switch** | **Sum** |
|  | **Target 1** | **Target 2** | **Target 3** | **T1 - T3** |  | **Target 1** | **Target 2** | **Target 3** | **T1 - T3** |  | **Target 1** | **Target 2** | **Target 3** | **T1 - T3** | **Cue** |  | **Target 1** | **Target 2** | **Target 3** | **T1 - T3** | **Cue** | |  | |  |  |  |
| **Subject 01** | 603.6±25.2 | 499.7±17.0 | 487.1±15.8 | 116.5±28.9 |  | 627.3±27.6 | 560.5±22.8 | 539.7±19.2 | 87.6±31.3 |  | 0.00±0.0 | 1.25±1.2 | 1.25±1.2 | -1.25±1.2 | 2.50±1.7 |  | 1.25±1.2 | 6.25±2.7 | 2.50±1.7 | -1.25±2.2 | 10.00±3.4 | |  | | 78 | 72 | 150 |
| **Subject 02** | 506.5±20.8 | 467.7±13.5 | 477.9±14.3 | 28.5±24.6 |  | 577.3±26.7 | 543.0±21.0 | 492.3±20.1 | 85.0±36.1 |  | 1.25±1.2 | 1.25±1.2 | 1.25±1.2 | 0.00±1.8 | 3.75±2.1 |  | 2.50±1.7 | 2.50±1.7 | 2.50±1.7 | 0.00±2.5 | 7.50±2.9 | |  | | 77 | 74 | 151 |
| **Subject 03** | 434.4±10.2 | 460.8±22.1 | 450.3±10.2 | -15.9±14.8 |  | 449.8±14.9 | 475.4±12.8 | 458.7±21.0 | -8.9±24.2 |  | 11.25±3.5 | 2.50±1.7 | 1.25±1.2 | 10.00±3.8 | 13.75±3.9 |  | 8.75±3.2 | 7.50±2.9 | 2.50±1.7 | 6.25±3.7 | 18.75±4.4 | |  | | 69 | 65 | 134 |
| **Subject 04** | 393.1±15.8 | 374.4± 9.5 | 370.9± 6.4 | 22.2±16.5 |  | 394.5±14.5 | 410.4±14.3 | 370.1± 5.8 | 24.4±15.5 |  | 2.50±1.7 | 1.25±1.2 | 3.75±2.1 | -1.25±2.8 | 7.50±2.9 |  | 2.50±1.7 | 3.75±2.1 | 8.75±3.2 | -6.25±3.7 | 13.75±3.9 | |  | | 74 | 69 | 143 |
| **Subject 05** | 588.8±25.1 | 485.4±23.2 | 496.2±23.4 | 92.6±29.9 |  | 633.9±28.2 | 526.3±25.6 | 519.0±24.6 | 114.9±36.3 |  | 5.00±2.4 | 2.50±1.7 | 2.50±1.7 | 2.50±3.1 | 10.00±3.4 |  | 6.25±2.7 | 5.00±2.4 | 2.50±1.7 | 3.75±3.3 | 13.75±3.9 | |  | | 72 | 69 | 141 |
| **Subject 06** | 468.6±12.9 | 444.3± 8.5 | 474.6±12.5 | -6.0±17.9 |  | 527.1±21.8 | 482.9±16.9 | 517.5±25.5 | 9.6±32.6 |  | 0.00±0.0 | 0.00±0.0 | 0.00±0.0 | 0.00±0.0 | 0.00±0.0 |  | 1.25±1.2 | 3.75±2.1 | 3.75±2.1 | -2.50±2.5 | 8.75±3.2 | |  | | 80 | 73 | 153 |
| **Subject 07** | 571.4±22.5 | 552.6±27.2 | 564.4±25.5 | 7.0±29.0 |  | 670.8±34.5 | 545.7±24.3 | 563.1±28.3 | 107.7±43.7 |  | 1.25±1.2 | 2.50±1.7 | 0.00±0.0 | 1.25±1.2 | 3.75±2.1 |  | 2.50±1.7 | 3.75±2.1 | 0.00±0.0 | 2.50±1.8 | 6.25±2.7 | |  | | 77 | 75 | 152 |
| **Subject 08** | 421.1±13.7 | 437.5±12.7 | 451.2±14.1 | -30.1±16.4 |  | 449.6±17.1 | 472.7±20.7 | 444.4±19.0 | 5.2±25.9 |  | 2.50±1.7 | 0.00±0.0 | 0.00±0.0 | 2.50±1.8 | 2.50±1.7 |  | 1.25±1.2 | 8.75±3.2 | 3.75±2.1 | -2.50±2.5 | 13.75±3.9 | |  | | 78 | 69 | 147 |
| **Subject 09** | 435.9±10.6 | 417.8± 9.3 | 413.5±10.6 | 22.4±13.5 |  | 533.1±31.4 | 456.5±12.8 | 433.3±10.6 | 99.8±31.0 |  | 0.00±0.0 | 0.00±0.0 | 1.25±1.2 | -1.25±1.2 | 1.25±1.2 |  | 1.25±1.2 | 2.50±1.7 | 2.50±1.7 | -1.25±2.2 | 6.25±2.7 | |  | | 79 | 75 | 154 |
| **Subject 10** | 528.8±15.7 | 481.9±11.1 | 486.7±10.7 | 42.1±17.9 |  | 564.7±16.1 | 500.7±11.7 | 509.5±13.8 | 55.1±18.0 |  | 2.50±1.7 | 0.00±0.0 | 0.00±0.0 | 2.50±1.8 | 2.50±1.7 |  | 1.25±1.2 | 2.50±1.7 | 0.00±0.0 | 1.25±1.2 | 3.75±2.1 | |  | | 78 | 77 | 155 |
| **Subject 11** | 554.3±30.2 | 414.7±11.8 | 435.4±12.9 | 118.9±30.3 |  | 599.0±34.9 | 468.7±22.1 | 470.9±19.9 | 128.1±39.5 |  | 2.50±1.7 | 1.25±1.2 | 1.25±1.2 | 1.25±2.2 | 3.75±2.1 |  | 8.75±3.2 | 5.00±2.4 | 2.50±1.7 | 6.25±3.7 | 15.00±4.0 | |  | | 77 | 68 | 145 |
| **Subject 12** | 682.1±38.2 | 512.5±21.3 | 542.8±16.0 | 139.3±41.9 |  | 792.3±35.0 | 645.6±32.4 | 594.0±26.1 | 198.3±40.6 |  | 2.50±1.7 | 2.50±1.7 | 0.00±0.0 | 2.50±1.8 | 5.00±2.4 |  | 10.00±3.4 | 6.25±2.7 | 3.75±2.1 | 6.25±4.1 | 16.25±4.1 | |  | | 76 | 67 | 143 |
| **Subject 13** | 486.1±21.0 | 427.2±11.8 | 425.1±14.7 | 61.0±26.5 |  | 588.4±28.2 | 463.1±17.4 | 436.1±17.8 | 152.4±32.1 |  | 0.00±0.0 | 2.50±1.7 | 2.50±1.7 | -2.50±1.8 | 5.00±2.4 |  | 3.75±2.1 | 2.50±1.7 | 2.50±1.7 | 1.25±2.2 | 6.25±2.7 | |  | | 76 | 75 | 151 |
| **Subject 14** | 790.8±40.5 | 725.3±42.4 | 707.3±38.9 | 83.4±57.3 |  | 917.3±45.4 | 666.5±33.2 | 605.9±28.4 | 311.4±52.5 |  | 5.00±2.4 | 1.25±1.2 | 3.75±2.1 | 1.25±3.3 | 8.75±3.2 |  | 7.50±2.9 | 1.25±1.2 | 6.25±2.7 | 1.25±4.2 | 15.00±4.0 | |  | | 73 | 68 | 141 |
| **Subject 15** | 548.4±21.6 | 464.1±14.9 | 492.4±15.2 | 56.1±27.6 |  | 611.9±28.9 | 524.0±18.7 | 485.3±16.8 | 126.5±35.4 |  | 1.25±1.2 | 0.00±0.0 | 0.00±0.0 | 1.25±1.2 | 1.25±1.2 |  | 5.00±2.4 | 3.75±2.1 | 1.25±1.2 | 3.75±2.8 | 10.00±3.4 | |  | | 79 | 72 | 151 |
| **Subject 16** | 629.6±36.0 | 475.6±26.0 | 453.4±14.6 | 176.2±36.5 |  | 703.0±31.6 | 460.5±21.3 | 477.0±22.0 | 226.0±34.5 |  | 2.50±1.7 | 5.00±2.4 | 2.50±1.7 | 0.00±2.5 | 10.00±3.4 |  | 7.50±2.9 | 3.75±2.1 | 3.75±2.1 | 3.75±3.7 | 13.75±3.9 | |  | | 72 | 69 | 141 |
| **Subject 17** | 619.0±22.2 | 533.1±21.1 | 532.6±22.5 | 86.4±28.0 |  | 825.0±38.9 | 586.4±32.5 | 526.3±23.8 | 298.7±46.2 |  | 2.50±1.7 | 3.75±2.1 | 1.25±1.2 | 1.25±1.2 | 6.25±2.7 |  | 6.25±2.7 | 3.75±2.1 | 2.50±1.7 | 3.75±3.3 | 11.25±3.5 | |  | | 75 | 71 | 146 |
| **Subject 18** | 730.5±34.6 | 575.4±27.2 | 561.9±31.1 | 168.5±44.3 |  | 896.1±40.8 | 645.7±33.3 | 524.9±22.5 | 371.1±45.1 |  | 1.25±1.2 | 0.00±0.0 | 2.50±1.7 | -1.25±2.2 | 3.75±2.1 |  | 11.25±3.5 | 2.50±1.7 | 0.00±0.0 | 11.25±3.6 | 12.50±3.7 | |  | | 77 | 70 | 147 |
| **Subject 19** | 648.3±29.2 | 650.8±32.8 | 663.9±33.8 | -15.6±44.4 |  | 751.9±36.8 | 724.6±36.8 | 613.2±24.2 | 138.7±43.1 |  | 0.00±0.0 | 3.75±2.1 | 3.75±2.1 | -3.75±2.1 | 7.50±2.9 |  | 11.25±3.5 | 5.00±2.4 | 3.75±2.1 | 7.50±3.9 | 17.50±4.2 | |  | | 74 | 66 | 140 |
| **Subject 20** | 630.5±26.0 | 539.3±16.2 | 513.9±18.5 | 116.5±28.3 |  | 665.3±32.8 | 586.4±21.7 | 544.7±22.8 | 120.6±36.7 |  | 1.25±1.2 | 0.00±0.0 | 0.00±0.0 | 1.25±1.2 | 1.25±1.2 |  | 0.00±0.0 | 0.00±0.0 | 2.50±1.7 | -2.50±1.8 | 2.50±1.7 | |  | | 79 | 78 | 157 |
| **Mean** | 563.6± 6.2 | 497.0± 5.1 | 500.1± 4.8 | 63.5± 7.0 |  | 638.9± 7.6 | 537.3± 5.7 | 506.3± 5.0 | 132.6± 8.4 |  | 2.25±0.4 | 1.56±0.3 | 1.44±0.3 | 0.81±0.5 | 5.00±0.5 |  | 5.00±0.5 | 4.00±0.5 | 2.88±0.4 | 2.12±0.7 | 11.12±0.8 | |  | | 76 | 71.1 | 147 |

*Note.* Reaction times (RTs) and error rates (ERs), along with their respective standard errors (SEs), are listed for all participants in both cue and target trials. The “Target 1–3” columns indicate the first three targets following a cue. The “T1–T3” columns indicate increased RTs or ERs in the first target trial compared to the third, reflecting the restart cost at either the individual or group level. The “Cue” columns refer to the cue response rate, defined as the overall accuracy across three consecutive post-cue targets. The “Valid Cue Trial” column shows the number of correctly responded cue trials out of 80 ‘repeat’ and 80 ‘switch’ cue trials. The “Mean” row displays the average RTs and ERs across all corresponding trials.

**Supplementary Table 2**

*Cross-Reference Table of Brain Regions among Different Anatomical Atlases*

| Abbreviation | Desikan-Killiany Regions | AAL90 Regions | Talairach and Broadmann Areas |
| --- | --- | --- | --- |
| ACCc | Caudal Anterior Cingulate | Anterior Cingulate, Paracingulate Gyrus (caudal) | Limbic Lobe, Cingulate Gyrus,24 |
| ACCr | Rostral Anterior Cingulate | Anterior Cingulate, Paracingulate Gyrus (rostral) | Limbic Lobe, Anterior Cingulate, 32 |
| CAL | Pericalcarine | Calcarine Fissure, Surrounding Cortex | Occipital Lobe, Cuneus, 17 |
| CUN | Cuneus | Cuneus | Occipital Lobe, Cuneus, 18 |
| EC | Entorhinal Cortex | Superior Temporal Pole | Limbic Lobe, Entorhinal Cortex, 28 |
| FFG | Fusiform Gyrus | Fusiform Gyrus | Fusiform Gyrus, 20 |
| HES | Transversetemporal | Heschl Gyrus | Temporal Lobe, Superior Temporal Gyrus,41&42 |
| IFGoperc | Parsopercularis | Inferior Frontal Gyrus (opercular) | Frontal Lobe, Inferior Frontal Gyrus, 44 |
| IFGorb | Parsorbitalis | Inferior Frontal Gyrus (orbital) | Frontal Lobe, Inferior Frontal Gyrus, 47 |
| IFGtri | Parstriangularis | Inferior Frontal Gyrus (triangular) | Frontal Lobe, Inferior Frontal Gyrus, 45 |
| INS | Insula | Insula | Sub-lobar, Insula, 13 |
| IPL | Inferior Parietal Lobule | Inferior Parietal Lobule | Inferior Parietal Lobule, 39 |
| ITG | Inferior Temporal Gyrus | Inferior Temporal Gyrus | Temporal Lobe, Inferior Temporal Gyrus, 20 |
| LING | Lingual Gyrus | Lingual Gyrus | Occipital Lobe, Lingual Gyrus, 18 |
| MCG | Posterior Cingulate Gyrus | Median Cingulate, Paracingulate Gyrus | Limbic Lobe, Cingulate Gyrus, 24 |
| MFGc | Caudal Middle Frontal Gyrus | Middle Frontal Gyrus (caudal) | Frontal Lobe, Middle Frontal Gyrus, 6 |
| MFGr | Rostral Middle Frontal Gyrus | Middle Frontal Gyrus (rostral) | Frontal Lobe, Middle Frontal Gyrus, 10 |
| MOG | Lateral Occipital Gyrus | Middle Occipital Gyrus | Occipital Lobe, Middle Occipital Gyrus, 18 |
| MTGv | Middle Temporal Gyrus | Middle Temporal Gyrus (ventral) | Temporal Lobe, Middle Temporal Gyrus, 21 |
| MTGd | Bankssts | Middle Temporal Gyrus (dorsal) | Temporal Lobe, Middle Temporal Gyrus, 22 |
| ORBsup | Lateral Orbito Frontal Gyrus | Superior Frontal Gyrus (orbital) | Frontal Lobe, Orbital Gyrus, 11 |
| ORBmid | Medial Orbito Frontal Gyrus | Middle Frontal Gyrus (orbital) | Frontal Lobe, Orbital Gyrus, 11 |
| PCG | Isthmus Cingulate Gyrus | Posterior Cingulate Gyrus | Limbic Lobe, Posterior Cingulate, 31 |
| PCL | Paracentral Lobule | Paracentral Lobule | Frontal Lobe, Paracentral Lobule, 6 |
| PCUN | Precuneus | Precuneus | Parietal Lobe, Precuneus, 7 |
| PHG | Parahippocampal Gyrus | Parahippocampal Gyrus | Limbic Lobe, Parahippocampal Gyrus, 36 |
| PoCG | Postcentral Gyrus | Postcentral Gyrus | Parietal Lobe, Postcentral Gyrus, 1 |
| PreCG | Precentral Gyrus | Precentral Gyrus | Frontal Lobe, Precentral Gyrus, 4 |
| SFGdor | Frontal Pole | Superior Frontal Gyrus (dorsolateral) | Frontal Lobe, Superior Frontal Gyrus, 10 |
| SFGmed | Superior Frontal Gyrus | Superior Frontal Gyrus (medial) | Frontal Lobe, Superior Frontal Gyrus, 8 |
| SMG | Supramarginal Gyrus | Supramarginal Gyrus | Parietal Lobe, Supramarginal Gyrus, 40 |
| SPL | Superior Parietal Lobule | Superior Parietal Lobule | Parietal Lobe, Superior Parietal Lobule, 7 |
| STG | Superior Temporal Gyrus | Superior Temporal Gyrus | Temporal Lobe, Superior Temporal Gyrus, 22 |
| TPO | Temporal Pole | Middle Temporal Pole | Temporal Lobe, Superior Temporal Gyrus, 38 |

*Note.* The abbreviations of the ROIs are based on the DK atlas. To ensure the comparability with other task-switching literature, these regions are additionally mapped onto the Anatomical Automatic Labeling (AAL) atlas (Rolls et al., 2020), the Talairach template (Lancaster et al., 2000), and Brodmann areas (Maldjian et al., 2003).

**Supplementary Table 3**

*Model Performance Comparison on the Pattern Recognition of Proactive Task-Switching*

|  | 500–800 ms Time Window | | | | |  | 900–1100 ms Time Window | | | | |
| --- | --- | --- | --- | --- | --- | --- | --- | --- | --- | --- | --- |
|  | Accuracy | AUROC | MCC | F1 | LOSS |  | Accuracy | AUROC | MCC | F1 | LOSS |
| Baseline |  |  |  |  |  |  |  |  |  |  |  |
| MAR(1) | .517(0.015)** | .529(0.013)** | .050(0.022)** | .524(0.016)** | 0.692(0.002)** |  | .514(0.012)** | .523(0.010)** | .045(0.019)** | .516(0.011)** | .693(0.001)** |
| ML |  |  |  |  |  |  |  |  |  |  |  |
| SVC | .555(0.042)** | .572(0.043)** | .120(0.087)** | .540(0.039)** | 0.687(0.005)** |  | .549(0.024)** | .575(0.020)** | .113(0.047)** | .532(0.025)** | .687(0.003)** |
| LightGBM | .577(0.026)** | .610(0.019)** | .164(0.048)** | .570(0.028)** | 0.679(0.006)** |  | .564(0.035)** | .591(0.038)** | .140(0.057)** | .560(0.031)** | .684(0.004)** |
| CNN |  |  |  |  |  |  |  |  |  |  |  |
| 1D-CNN | .661(0.037)** | .720(0.050)** | .332(0.075)** | .665(0.030)** | 0.632(0.021)** |  | .656(0.078)** | .708(0.093)** | .324(0.155)** | .659(0.074)** | .638(0.047)** |
| RNN |  |  |  |  |  |  |  |  |  |  |  |
| LSTM | .728(0.061)* | .797(0.067)* | .462(0.121)* | .732(0.058)* | 0.570(0.062)* |  | .724(0.066)* | .792(0.079)* | .451(0.132)* | .732(0.064)* | .569(0.059)* |
| GRU | .747(0.055)* | .818(0.066)* | .498(0.110)* | .755(0.053)* | 0.551(0.060)* |  | .712(0.081)* | .780(0.093)** | .429(0.162)* | .719(0.076)* | .582(0.080)** |
| CRNN |  |  |  |  |  |  |  |  |  |  |  |
| TPA-LSTM | .762(0.053)* | .835(0.056)* | .527(0.106)* | .770(0.054)* | 0.524(0.063)* |  | .744(0.063)* | .815(0.068)* | .495(0.125) | .748(0.062)* | .556(0.068)* |
| RGNN |  |  |  |  |  |  |  |  |  |  |  |
| EvolveGCN-O | .775(0.084) | .849(0.088) | .553(0.169) | .780(0.085) | 0.504(0.090)* |  | .770(0.067)* | .845(0.070)* | .543(0.133) | .778(0.068)* | .514(0.083) |
| DyGGNN | .780(0.065) | .850(0.065) | .563(0.129) | .786(0.066) | 0.491(0.081) |  | .769(0.062)* | .840(0.066)* | .542(0.124) | .776(0.065)* | .518(0.078) |
| EvoloGCN-H | .815(0.047) | .889(0.048) | .632(0.092) | .821(0.044) | 0.441(0.076) |  | .792(0.087) | .867(0.084) | .588(0.174) | .799(0.087) | .484(0.092) |
| T-GCN | .827(0.032) | .902(0.032) | .657(0.061) | .833(0.031) | 0.427(0.064) |  | .816(0.038) | .896(0.041) | .634(0.075) | .823(0.038) | .438(0.068) |

*Note.* For each evaluation metric, the mean (*SD*) across all validation sets in the cross-validation procedure is reported. Models are ranked in ascending order based on accuracy in the 500–800 ms window. */** indicate that a model’s performance on the corresponding metric is significantly worse than that of T-GCN under cross-validation, as determined by one-sided t-tests, with *p*s < .010 and .001, respectively.

**Supplementary Table 4**

*Common and Differential ACM-FCs during* *Proactive Task Switching*

| Duration | Common ACM-FC |  | Differential ACM-FC | |
| --- | --- | --- | --- | --- |
| Method |  |  | Switch＞Repeat | Repeat＞Switch |
| 500–800 ms |  |  |  |  |
| Coh | FFG.L-PHG.L * |  | MFGr.R-SFGmed.R | EC.L-MFGr.L * |
|  | STG.L-INS.L * |  | STG.L-INS.L |  |
| ImCoh | **ITG.L-PHG.L** |  | MFGr.R-SFGmed.R | EC.L-MFGr.L * |
|  | **PCL.L-PCUN.L** |  | **SFGdor.L-PCL.L** | EC.L-ORBsup.L * |
|  | STG.L-INS.L * |  |  |  |
| PPC | SFGmed.L-MCG.L * |  | **SFGdor.L-SFGdor.R** |  |
|  | **MTGv.L-INS.L** |  | IFGorb.L-INS.L |  |
|  | **IFGoperc.L-INS.L** |  | SFGdor.L-PreCG.L * |  |
|  | **ITG.L-INS.L** |  | **ITG.L-PHG.L** |  |
|  |  |  | **SFGdor.L-PCL.L** |  |
| PLV | **SMG.L-MTGv.L** |  | **SFGdor.L-SFGdor.R** |  |
|  | **SFGdor.L-SFGdor.R** |  | MTGd.L-TPO.L |  |
|  | **ITG.L-INS.L** |  | **ITG.L-PHG.L** |  |
|  | **MFGr.L-ACCc.L** |  | ORBsup.R-SFGdor.R |  |
|  | **MTGv.L-INS.L** |  |  |  |
| 900–1100 ms |  |  |  |  |
| Coh | **ITG.L-INS.L** |  | IFGtri.R-INS.R * | **IFGoperc.L-IFGtri.L** |
|  | MTGv.L-TPO.L * |  |  |  |
| ImCoh | MTGv.L-INS.L * |  | **IFGoperc.R-INS.R** | **IFGoperc.L-INS.L** |
|  | **ITG.L-PHG.L** |  | IFGtri.R-INS.R * | **IFGoperc.L-IFGtri.L** |
|  | **ITG.L-INS.L** |  |  |  |
|  | MTGv.L-TPO.L * |  |  |  |
| PCC | **ITG.L-PHG.L** |  |  | MFGc.L-SFGmed.L |
|  | **ITG.L-INS.L** |  |  | **IFGoperc.L-INS.L** |
|  | **ITG.L-TPO.L** |  |  | **IFGoperc.L-IFGtri.L** |
|  | **ITG.L-TPO.L** |  |  |  |
|  | **IFGoperc.L-INS.L** |  |  |  |
| PLV | **ITG.L-INS.L** |  |  | MFGc.L-SFGmed.L |
|  | **ITG.L-PHG.L** |  |  | **IFGoperc.L-IFGtri.L** |
|  | IFGtri.L-INS.L * |  |  | **IFGoperc.L-INS.L** |
|  | MTGv.L-INS.L * |  |  | MFGc.L-IFGtri.L * |
|  | **ITG.L-TPO.L** |  |  |  |

*Note.* The table shows Anatomical-Connectivity-Masked Functional Connections (ACM-FCs), which are FC metrics retained after being masked by a group-level structural template during the preparatory phase of proactive task switching. ACM-FCs in bold indicate those that also appear among the ACG-FCs of the same type within the same time window. An asterisk (*) denotes that at least one of the two regions involved in the ACM-FC is included among the regions implicated in the ACG-FCs of the same type within the same time window.
